# Evaluation of larvicidal potential against larvae of *Aedes aegypti* (Linnaeus, 1762) and of the antimicrobial activity of essential oil obtained from the leaves of *Origanum majorana* L.

**DOI:** 10.1371/journal.pone.0235740

**Published:** 2020-07-17

**Authors:** Renata do Socorro Barbosa Chaves, Rosany Lopes Martins, Alex Bruno Lobato Rodrigues, Érica de Menezes Rabelo, Ana Luzia Ferreira Farias, Lethicia Barreto Brandão, Lizandra Lima Santos, Allan Kardec Ribeiro Galardo, Sheylla Susan Moreira da Silva de Almeida

**Affiliations:** 1 Laboratory of Pharmacognosy and Phytochemistry, Federal University of Amapá, Macapá, Amapá, Brasil; 2 Laboratory of Entomology Medical of Institute of Scientific and Technological Research of the State of Amapá (IEPA), Macapá, Amapá, Brasil; University of British Columbia, CANADA

## Abstract

This study evaluated the larvicidal activity of *Origanum majorana* Linnaeus essential oil, identified the chemical composition, evaluated the antimicrobial, cytotoxic and antioxidant potential. The larvicidal activity was evaluated against larvae of the third stage of *Aedes aegypti* Linaeus, whereas the chemical composition was identified by gas chromatography coupled to mass spectrometer, the antimicrobial activity was carried out against the bacteria *Pseudomonas aeruginosa*, *Escherichia coli* and *Staphylococcus auereus*, the antioxidant activity was evaluated from of 2.2-diphenyl-1-picryl-hydrazila sequestration and *Artemia salina* Leach cytotoxicity. Regarding to the results, the larvicidal activity showed that *O*. *majorana* L. essential oil caused high mortality in *A*. *aegypti* L. larvae. In the chromatographic analysis, the main component found in *O*. *majorana* L. essential oil was pulegone (57.05%), followed by the other components verbenone (16.92%), trans-p-menthan-2-one (8.57%), iso-menthone (5.58%), piperitone (2.83%), 3-octanol (2.35%) and isopulegol (1.47%). The antimicrobial activity showed that *E*. *coli* and *P*. *aeruginosa* bacteria were more sensitive to oil than *S*. *aureus*, which was resistant at all concentrations. Essential oil did not present antioxidant activity, but it has high cytotoxic activity against *A*. *salina* L.

## Introduction

Dengue remains an important public health problem in Brazil, even after the introduction and recent dissemination of the Zika and chikungunya viruses [1,2]. The disease presents a great epidemic potential, affecting all regions of Brazil [3,4].

The *Aedes aegypti* Linnaeus [5] mosquito is a vector of viruses that cause diseases known as dengue, chikungunya, and zika [6]. It has holometabolic development, with egg, larva, pupa and adult phases. Because it is a mosquito highly adapted to the urban environment, its most common breeding sites are artificial containers that accumulate water, such as bottles, tires, cans, and pots [7].

Among the control policies adopted in Brazil, the mechanical control is carried out by ACE (Agents to Combat Endemics), with the participation of the population, aiming at the protection, destruction or adequate allocation of potent breeding sites. The intensive collaboration of the population is crucial to hinder the proliferation and installation of the mosquito. In addition, it reinforces the need for adequate sanitary conditions in the cities, eliminating stocks of water that allow eggs to hatch. An important strategy is the promotion of educational actions during home visits made regularly by the health agents [8].

The spread and flow of various serotypes of the dengue virus over the years also have a significant influence on epidemics, as well as an increase in cases diagnosed for the most severe form of the disease. These factors demonstrate the importance of introducing preventive measures in order to reduce dengue rates [9].

To combat these disease vectors, insecticides are the most used products, but they have several disadvantages, as they can be the source of several environmental problems [10, 11]. In fact, most of the chemical insecticides used cause a major problem, especially the development of mosquito resistance [12, 13].

In addition, the literature reports that *Origanum majorana* species have attracted consumers' attention due to their antimicrobial, antifungal, insecticidal and antioxidant effects on human health [14]. The crude extract of *O*. *majorana* and essential oil show significant results in inhibiting the growth of bacteria and fungi and the synthesis of microbial metabolites [15–17].

*Origanum majorana* Linnaeus [18] belongs to the Lamiaceae [19] family, and it contains several terpenoids, which are isolated from aerial parts of the *Origanum* plant and exhibit antimicrobial, antiviral and antioxidant properties, without toxic effects [20, 21].

Previous studies have reported the potential use of the *O*. *majorana* ethanolic extract as an anticancer agent [22, 23], while the tea extract has been shown to have immunostimulating, antigenotoxic and antimutagenic properties [24, 25]. These activities are attributed to the chemical composition, characterized as rich in flavonoids and terpenoids [26].

The antioxidant activity of essential oils through the elimination of free radicals and inhibition of oxidation of linoleic acid, can be useful for the food industry to prolong the stability of food storage [27].

Thus, the search for natural antimicrobial compounds has been of great interest at the scientific and clinical level, so that aromatic and medicinal plants play a central role in the search for biologically active molecules, against different microorganisms [28].

The antimicrobial and antioxidant properties of many spices and their essential oils have been known for a long time, but only in recent years have consumers given proper attention to the use of these substances [29]. Because many plants are toxic to mosquitoes, the mixture of essential oils may represent an efficient outlet for this problem, compared to the *A*. *aegypti* L. mosquito [30].

In the literature, there are no reports on larvicidal activity against *A*. *aegypti* L. and cytotoxicity against *A*. *salina* L. and few studies have been reported on the antioxidant and antimicrobial effects of the essential oils of this species.

The active effects of the *O*. *majorana* species are not as studied and especially the larvicidal effect. So far, we have not found such studies related to this species with unique chemical composition in the country. Thus, in this article a first study was carried out on the larvicidal activity of essential oil.

It is in this sense that the work was carried out for the first time in Macapá and aims to study the larvicidal activity of the essential oils of *O*. *majorana* (Lamiaceae), cultivated in the north of the state against the larvae of *A*. *aegypti*, the vector of the dengue virus, Zika, chikungunya and malaria. In addition, to determine the chemical composition, to evaluate the antimicrobial activity against *E*. *coli*, *P*. *aeruginosa* and *S*. *aureus* bacteria, to determine the antioxidant potential through the sequestration of DPPH and cytotoxicity against *A*. *salina* L. of *O*. *majorana* L. essential oil.

## Materials and methods

### Plant material

The leaves of *O*. *majorana* L. were collected in the district of Fazendinha (00 "36'955" S and 51 "11'03'77" W) in the Municipality of Macapá, Amapá. Five samples of the plant species were deposited at the Amapaense Herbarium (HAMAB) of the Institute of Scientific Research and Technology of Amapá (IEPA).

### Essential oil obtaining

The hydrodistillation process using the Clevenger type apparatus, 131 g of *O*. *majorana* L. dried leaves were dried at 45 °C for a period of 2 h [31] obtained the essential oil (EO). The EO was kept under refrigeration (4 °C).

### Identification of the chemical composition by gas chromatography coupled to mass spectrometer (GC-MS)

The EO analysis was performed by Gas Chromatography coupled to the Mass Spectrometer (GC-MS) of the Museu Paraense Emílio Goeldi. The Shimadzu equipment, model GCMS-QP 5000 A was used. A fused silica capillary column (OPTIMA^®^-5-0.25 μm) was used. It has 30 m of length and 0.25 mm of internal diameter and nitrogen as carrier gas. The operating conditions of the gas chromatograph were: internal column pressure 67.5 kPa, division ratio 1:20, gas flow at column 1.2 mL.min^-1^ (210 °C), injector temperature 260 °C, temperature detector or interface of 280 °C. The initial column temperature was 50 °C, followed by an increase from 6 °C.min^-1^ to 260 °C kept constant for 30 min. The mass spectrometer was programmed to perform readings at intervals of 29–400 Da, at intervals of 0.5 s with ionization energy of 70 eV. 1 μL of each sample with a concentration of 10.000 ppm dissolved in hexane was injected.

The identification of the chemical compounds present in the EO was made from the comparisons of the Indices of Retention (IR) and Kovats (IK) of the homologous series of n-alkanes (C8-C26) and the literature [32]. Identification was also made by combining the spectra obtained by the analysis performed on the Lab solutions GC-MS version 2.50 Sigma–Aldrich, St. Louis, MO, USA e software equipment of the mass spectra of the NIST05 and WILEY'S libraries.

### Larvicidal activity against *A*. *aegypti* L. larvae

The data from this study were interpreted to identify compounds that showed toxicity of the essential oil of *O*. *majorana*. The identification of the active substances against *A*. *aegypti* larvae helped to interpret the toxicity of the essential oil.

The *A*. *aegypti* L. larvae used in the bioassay came from the colony (strain Rockfeller) kept in the Medical Entomology Laboratory of the Institute of Scientific and Technological Research of the State of Amapá (IEPA). The methodology used followed the World Health Organization standard protocol [33] with adaptations.

The procedure started with the separation of 18 beakers of 50 mL and in each Becker, there were added 25 larvae of the third instar of *A*. *aegypti* L. Then they were reserved in a room with conditions of ambient temperature between 25 to 30 °C and photoperiod of 12 h.

Preparation of the samples started after 24 h. The stock solution was prepared with 4.5 mL of Tween 80, 85.5 mL of distilled water and 0.09 g of the EO sample of *O*. *majorana* L. The positive control was prepared with 17.5 mL of Tween 80 dissolved in 350 mL of distilled water, and the larvicidal esbiothrin as the positive control.

After the preliminary tests, the aqueous solution was diluted in the following concentrations: 100, 80, 60, 40, 20, 10, and 1 μg.mL^−1^. Each concentration was tested in triplicate, and 25 larvae of the *A*. *aegypti* L. mosquito in the 3rd young stage (L3) were used. They were pipetted into a 100 mL beaker containing distilled water, then they were transferred into the test vessels, minimizing the time between the preparation of the first and last samples. During the experiment, the average water temperature was 25 °C. After 24 and 48 h, the dead larvae were counted, being considered as such, all those unable to reach the surface.

#### Statistical analysis

The experiment was carried out in triplicate. The larval mortality efficiency data were calculated in percentages using the Abbott formula and later tabulated in Microsoft Excel (Version 2013 for Windows). Probit analysis was performed with determination of the LC_50_ (lethal concentration causing 50% mortality in the population) and the LC_90_ (lethal concentration causing 90% mortality in the population) which were analyzed with a 95% confidence interval using the Statgraphics software Centurion XV version 15.2.11. The results were shown in the table. Differences that presented probability levels p≤0.001 for 24 h and p≤0.013 were considered statistically significant.

### Antimicrobial activity

#### Microorganisms

The antimicrobial EO test obtained from *O*. *majorana* L. leaves was tested in vitro against two gram-negative bacteria (*P*. *aeruginosa* ATCC 25922 and *E*. *coli* ATCC 8789) and a gram-positive bacteria (*S*. *aureus* ATCC 25922).

For each microorganism, the stock culture was stored in BHI medium (Brain Heart Infusion) with 20% glycerol and stored at –80 °C. An aliquot of 50 μL of this culture was inoculated into 5 mL of sterile BHI broth medium and incubated for 24 h at 37 °C.

#### Determination of minimum inhibitory concentration (MIC) and minimum bactericidal concentration (MBC)

The MIC and MBC were determined using the microplate dilution technique (96 wells) according to the protocol established by Clinical and Laboratory Standards Institute [34], with adaptations.

Bacteria were initially reactivated from the stock cultures, kept in BHI broth, for 18 h at 37 °C. Subsequently, bacterial growth was prepared in 0.9% saline inoculum for each microorganism, adjusted to the McFarland 0.5 scale, then diluted in BHI and tested at 2 x 106 UFC.mL^-1^ concentration.

In determining the MIC, the EO was diluted in Dimethylsulfoxide (2% DMSO). Each well of the plate was initially filled with 0.1 mL of 0.9% NaCl, except for the first column, which was filled with 0.2 mL of the EO at the concentration of 2000 μg.mL^-1^. Subsequently, base two serial dilutions were performed in the ratio of 1:2 to 1:128 dilution in a final volume of 0.1 mL. After this process, 0.1 mL of cells (2 x 106 CFU mL^-1^) added in each well related to the second preceding item, resulting in a final volume of 0.2 mL. Control of culture medium, control of EO, and negative control (DMSO 2%) were performed. And for the positive control, amoxicillin (0.5 μg.mL^-1^) was used. After incubation of the microplates in an incubator at 37 °C for 24 hours, the plates were read in ELISA reader (OD 630nm).

The determination of MBC was performed based on the results obtained in the MIC test. Microplate wells were replicated in Müller-Hinton agar and incubated at 37 °C for 24 h. MBC was established as the lowest concentration of EO capable of completely inhibiting microbial growth.

#### Statistical analysis

All experiments were performed in triplicate with the respective results categorized in Microsoft Excel (Version 2013 for Windows) and later analyzed in GraphPad Prism software (Version 6.0 for Windows, San Diego California USA). Significant differences between the groups were verified using the One-way ANOVA test with Bonferroni post-test. The data were considered statistically significant when p <0.001.

### Antioxidant activity

The antioxidant quantitative test was based on the methodology recommended by Sousa et al. [35], Lopes-Lutz et al. [36] and Andrade et al. [37] by the use of 2.2-diphenyl-1-picryl-hydrazila (DPPH) with adaptations.

A methanolic solution of DPPH (stock solution) was prepared at the concentration of 40 μg.mL^-1^, which was kept under the light. The EOs were diluted in methanol at concentrations 7.81; 15.62; 31.25; 62.5; 125 and 250 μg.mL^-1^. For the evaluation of the test, 0.3 mL of the oil solution was added to a test tube, followed by the addition of 2.7 mL of the DPPH solution. White was prepared from a mixture with 2.7 mL of methanol and 0.3 mL of the methanol solution of each EO concentration as measured. After 30 min the readings were performed on a spectrophotometer (Biospectro SP-22) at a wavelength of 517 nm. The test was performed in triplicate and the calculation of the percentage of antioxidant activity (% AA) was calculated with the following Eq 1:

$$(\%AA)=100-\left\{\frac{\left[(Abs_{sample}-Abs_{white})\cdot 100\right]}{Abs_{control}}\right\}$$ (1)

AA%—Percentage of antioxidant activity

Abs_sample_—Sample absorbance

Abs_white_—White absorbance

Abs_control_—Control absorbance

### Cytotoxic activity against *A*. *salina* L.

The cytotoxicity assay against *A*. *salina* L. Leach was based on the technique of Araújo et al. [38] and Lôbo et al. [39] with adaptations. An aqueous solution of artificial sea salt was prepared (35 g.L^-1^) at pH 9.0 for incubation of 45 mg of *A*. *salina* L. eggs, which were placed in the dark for 24 h for the larvae to hatch (nauplii), then the nauplii were exposed to artificial light in 24 h, period to reach the stage methanuplii. The stock solution was prepared to contain 0.06 g of EO, 28.5 mL of solution of synthetic and 1.5 mL of Tween 80 to facilitate solubilization of the same. The test tubes were marked up to 5 mL. For the negative control, it was used respectively Tween 80 with solution saline (5%) and the (K_2_Cr_2_O_7_) Potassium dichromate (1%) as the positive control.

The methanauplia were selected and divided into 7 groups of 10 subjects in each test tube. Each group received aliquots of the stock solution 100, 75, 50, 25, and 2.5 μL, which were then filled to a volume of 5 mL with the sea salt solution to produce final solutions with the following concentrations 40, 30, 20, 10, and 1 μg.mL^-1^. The tests were performed in triplicates. For the test control, saline solution was used. After 24 h, the number of dead was counted.

#### Statistical analysis

The results obtained from the bioassays were expressed through Averages and Standard Deviation, categorized in Microsoft Excel (Version 2010 for Windows, Redmond, WA, USA). Significant differences between treatments were assessed using the ANOVA test One criterion and the Tukey test using the BioEstat program (Version 5.0 for Windows, Belem, BRA). The graphs were built on GraphPad Prism software (Version 6.0 for Windows, San Diego, CA, USA). The LC_50_ values were determined in the PROBIT regression, through the SPSS statistical program (version 21.0 for Windows, Chicago, IL, USA). Differences that presented probability levels less than or equal to 5% (p ≤ 0.05) were considered statistically significant.

## Results and discussion

### Identification of chemical compounds by GC-MS of the *O*. *majorana* L. EO

**Fig 1:**
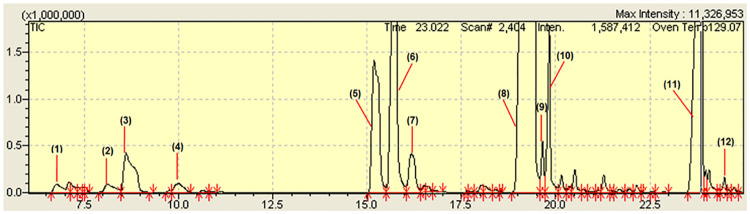
Obtaining gas chromatography of OE *O*. *majorana* L. gas trap: Helium (He); initial temperature 60 °C; initial time 1.0 min; the column temperature increased 3 °C/ min. at 240 °C, maintained at this temperature for 30 min.

This result corroborates with other studies that have shown that environmental factors may affect certain chemical compounds, while in others they have no influence on their production [40, 41]. The chemical composition was determined by GC-MS, where the chromatogram can be observed in Fig 1.

On the chemical composition of the EO of *O*. *majorana* L. (Table 1), 95.8% are oxygenated monoterpenes and only 1.38% are monoterpene hydrocarbons. The major component of EO is pulegone (57.05%), followed by other components verbenone (16.92%), trans-menthone (8.57%), cis-menthone (5.58%), piperitone (2.83%), 3-octanol %) and isopulegol (1.47%).

**Table 1:** Chemical composition of *O*. *majorana* L. essential oil.

N° *	IR	IK	Compounds	Relative Percentage (%)	Identification #
1	6.776	939	α-pinene	0.39	MS, IK
2	8.127	979	β-pinene	0.50	MS IK
**3**	**8.617**	**991**	**3-octanol**	**2.35**	**MS, IK**
4	9.995	1029	limonene	0.49	MS, IK
**5**	**15.185**	**1162**	**iso-menthone**	**5.58**	**MS, IK**
**6**	**15.688**	**1199**	**Trans-*p*-menthan-2-one**	**8.57**	**MS, IK**
**7**	**16.174**	**1149**	**isopulegol**	**1.47**	**MS, KI**
**8**	**19.448**	**1237**	**pulegone**	**57.05**	**MS, IK**
9	19.655	1165	lavandulol	0.77	MS, IK
**10**	**19.883**	**1252**	**piperitone**	**2.83**	**MS, IK**
**11**	**23.873**	**1205**	**verbenone**	**16.92**	**MS, IK**
12	24.482	1161	nonen-1-al-(2E)	0.26	MS, IK
			Total	97.18	

*The identification path of the compounds,

^#^the identification of the compounds was performed by the mass spectrum (GC-MS) of the Library softwary Labsolutions GC-MS solution version 2.50 SU1 (NIST05 and WILEY'S libraries of the 9th edition mass spectrum); Kovats Index (KI) [32].

Lima et al. [42], reports that piperitone has three organic functions in its chemical structure and it can be used for the synthesis of other compounds. Piperitone is derived from the metabolic pathway for the formation of piperitenone oxide, in which cis-pulegone is also, derived [43]. Macêdo et al. [44] observe that the variations of the active components of the plant are important parameters to correlate the activities, such as antibacterial and insecticide.

In addition, a number of biotic factors such as plant/ microorganism Stoppacher et al. [45], plants/insects Kessler and Baldwin [46] plant interactions, age and stage of development. As well as abiotic factors such as luminosity Takshak and Agrawal [47], temperature, precipitation, nutrition, time and harvest time Bitu et al. [48], they may present correlations with each other, acting together, and they may exert a joint influence on chemical variability and yield of essential oil [48].

### Larvicidal activity of EO of the *O*. *majorana* L. against *A*. *aegypti* L. larvae

The results of the larvicidal activity of this study show that *O*. *majorana* L. EO is active against *A*. *aegypti* L. larvae.

A fact that Komalamisra et al. [49], Magalhães et al. [50] and Dias et al. [51], classified with the values of the minimum lethal concentration that eliminates 50% of the population (LC_50_) as a criterion for the activity. Because if LC_50_ <50 μg.mL^-1^, the product is considered very active, if 50 <LC_50_ <100 μg.mL^-1^ the product is considered active, and when LC_50_> 750 μg.mL^-1^ the product is considered inactive.

The percentage of dead *A*. *aegypti* L. larvae is shown in Table 2, at different EO concentrations of *O*. *majorana* L. in the 24–48 h exposure period. There was no mortality in the control group. Through the probit test, LC_50_ = 62.81 μg.mL^-1^, determination coefficient (R^2^) = 78.64 and quantitative evaluation (*X*^*2*^) = 24.238 in 24 h. After 48 h at LC_50_ = 4.84 μg.mL^-1^, *X*^*2*^ = 10.3872 and R^2^ = 55.16.

**Table 2:** Percentage of dead larvae (%) of *A*. *aegypti* L. produced by different concentrations of *O*. *majorana* L. essential oil in 24–48 h.

Concentrations	Larvicidal Activity (%)	
(μg.mL^-1^)	24 h	48 h
Control (-)	0.0	0.0
20	16	57.33
40	40	76
60	40	77.33
80	65.32	82.66
100	78.62	94.66^a^
LC_50_ (positive control)	0.0034 μg.mL^-1^	0.0034 μg.mL^-1^

^a^ Statistically significant in relation to the positive control.

The probit analysis performed was not visible the slope values, but as the P of the model in Table 3 demonstrated a statistically significant relationship between the variables at the 95% confidence level in 24 h, thus, the adjusted percentage of 78,64% was more appropriate and more effective than in 48 h, allowing inferring that mortality was slower with increased concentration.

**Table 3:** Insecticide response-concentration on larvae of *A*. *aegypti*.

Time	CL_50_ (IC_95_) μg.mL^-1^	CL_90_ (IC_95_) μg.mL^-1^	GL	*X*^*2*^	R^2^
**24 h**	62.81 (50.87; 75.63)	124.17 (103.23; 171.63)	1	24.238	78.64
**48 h**	4.84 (NI; 27.95)	90.30 (70.16; 159.27)	1	10.3872	55.16

NI: not identified; CI confidence interval; X^2^; GL: Degree of Freedom; p <0.0001 for 24 h; p <0.0013 for 48 h.

There were no reports of studies on the larvicidal activity of *O*. *majorana* L. essential oil against *A*. *aegypti* L. larvae.

According to Cantrell et al. [52], larvicidal compounds act by absorption through the cuticle, via the respiratory tract, and/or enter by ingestion via the gastrointestinal tract. Once inside the larva, the substances may reach the site of action or may cause systemic effects by diffusion in different tissues [53].

Studies on the insecticidal effect of *Mentha* spp. reported that menthol, mentona, pulegone and carvone help to clarify the mechanisms of action on insects [54]. Previous studies indicate that limonene, camphene, and verbenone have insecticidal insect activity [55].

The effect of the tested essential oil of *O*. *majorana* L. can be explained by its chemical composition [56, 57]. The main components of essential oil, which belongs to the Lamiaceae family, are monoterpenes. In this context, the literature reports the larvicidal effect of monoterpene against mosquitoes [58, 59].

In the study by Pavela et al. [60] the essential oils of different species of *Mentha* L. and *Pulegium* showed varied chemical composition of monoterpenes, such as, for example, the major compound piperitone, which was effective and responsible for the insecticidal effects. Koliopoulos et al. [61] tested piperitenone oxide for larvicidal efficacy against the biotype *Culex pipiens* molestus and LC_50_ estimated at 9.95 mg/l.

In general terms, it can be considered that the main entrance to the channel in the larvae of mosquitoes exposed to larvicides is the cuticular. In this process, the polarity (expressed as an octanol-water partition coefficient) of the xenobiotic that entry through the integument plays an essential role in determining the rate of entry into the body [62]

The results found in the literature on the chemical composition of the essential oil of *O*. *majorana* L. differ with the results of the species of this study collected in Macapá. It is worth mentioning that the plant species have several very different types of chemo and, at times, the participation of the main constituent may be only minor in essential oil.

Recently, a study by El-Akhal et al. [63], demonstrated the insecticidal activity of *O*. *majorana* L. against *Culex pipiens*, the vector of the West Nile virus. LC_50_ and LC_90_. The obtained LC were 258.71μg/mL and 580.49 μg/mL, respectively.

The LC_50_ (107.13 μg/mL) and LC_90_ (365.90 μg/mL) of the essential oil of *O*. *majorana* L., found in the study by El-Kahal et al. [63] against *An*. *Labranchiae*, are significant when compared to those found in *T*. *vulgaris*. They are also very important compared to those found in *O*. *majorana* L. against *Culex pipiens* in research work carried out in the laboratory [59]. The non-significant difference between the essential oils tested: *T*. *vulgaris* and *O*. *majorana* L. (CI_95_ overlap) can be explained by their similar chemical composition [59, 63].

In fact, the main components of the two essential oils, which belong to the same family (Lamiaceae), are monoterpenes. In this context, the literature reports the larvicidal effect of monoterpene against mosquitoes [56].

Pavela and Sedlák [64] showed in their studies that insecticidal efficacy increased with temperature when OE was applied against *S*. *littoralis* larvae and revealed by a comparison of lethal doses, while LC_50_ and LC_90_ for *S*. *littoralis* larvae in 15° C were estimated at 52 and 84 μg/larva, respectively, LC_50_ and LC_90_ were significantly lower at 30° C (32 and 56 μg/larva, respectively).

Despite the insecticidal efficacy of OEs has been studied in many insect species, very little information is available on other limiting factors of the post-application configuration that can have a significant impact on the insecticidal efficacy of OE-based botanical insecticides. Understanding the relationship between post-application temperature and insecticidal efficacy of OE is important, particularly in terms of practical recommendations for applying botanical insecticides based on EOs [64].

That is why this study tests the insecticidal efficacy of an essential oil obtained from *Thymus vulgaris* L. (Lamiaceae), applied at different ambient temperatures. *T*. *vulgaris* E was chosen intentionally because, compared to other OEs, it provides significantly better insecticidal efficacy and, therefore, was considered a highly promising active ingredient for some potential and already manufactured BIs [65].

However, when applied in water against *C*. *quinquefasciatus* larvae, the opposite effect was found. The water at the lowest temperature reached the highest mortality rate of *C*. *quinquefasciatus* and LC_50_ (90) larvae were estimated at 21.3 (29.3) μg.L^-1^ at 15 °C, while LC_50_ (90) estimated 21.4 (33.1) μg.L^−1^ were obtained for the highest temperature tested (30° C). This suggests that the influence of post-application temperature on insect mortality varies not only by the method of application, but also on the chemical composition of the OE [64].

Some EOs are known to cause dissuasive or anti-eating behavior in insects suggesting a neurotoxic action Satyan et al. [66], while some act as growth-regulating insects through analogous effects or antagonistic endogenous hormones. In the present study, it was found that even short-term exposure of larvae to lethal doses can dramatically increase their mortality over time and thereby reduce the total number of viable adults, leading to a possible reduction in total populations [67].

### Microbiological activity of *O*. *majorana* L. EO

In relation to the microbiological activity, it was possible to verify that gram-negative bacteria were more sensitive to *O*. *majorana* L. EO, than gram-positive bacteria.

**Fig 2:**
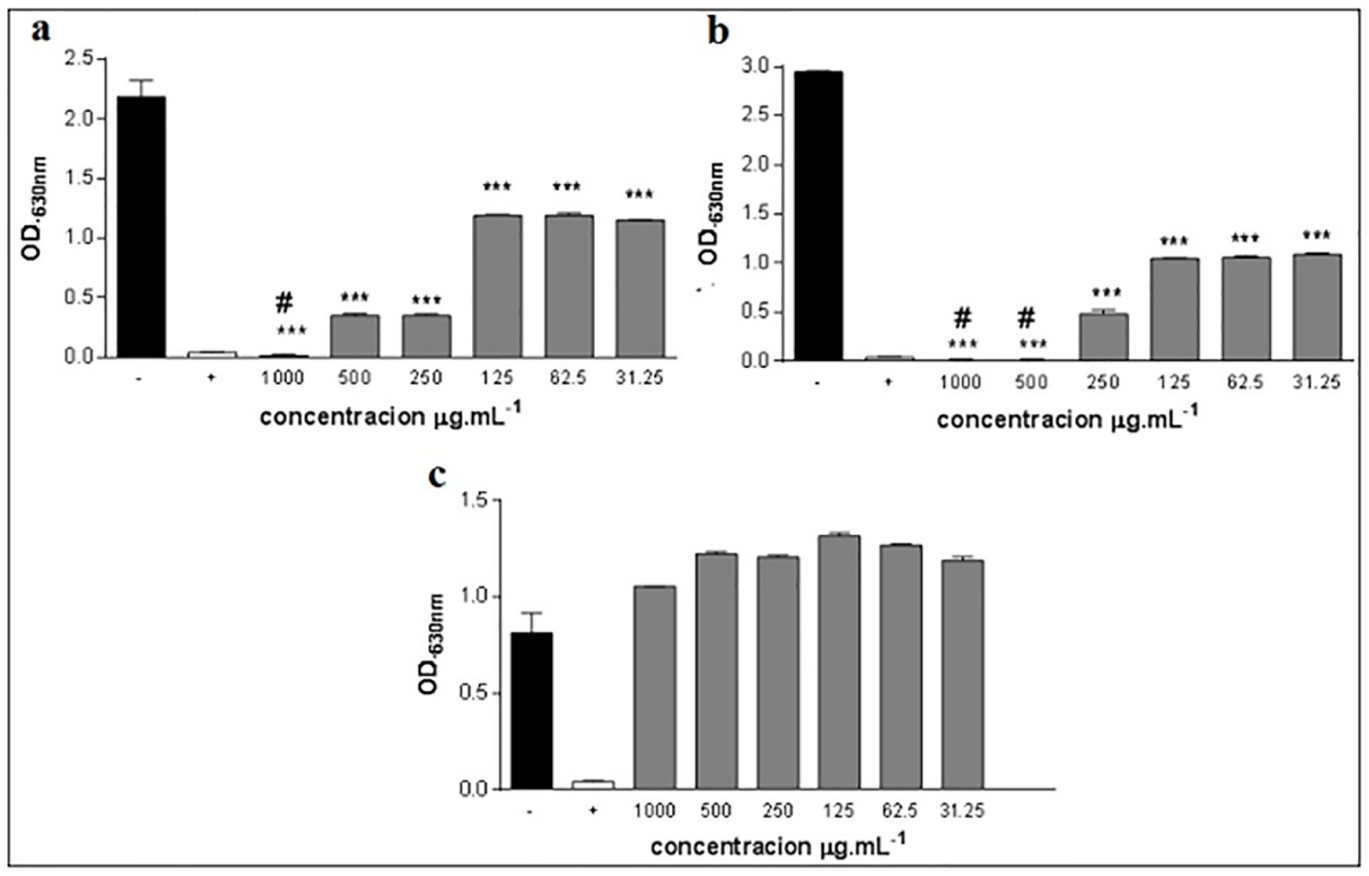
This is the figure of Minimum Inhibitory Concentration (MIC) and Minimum Bactericidal Concentration (MBC) of *O*. *majorana* L. essential oil. a) EO *O*. *majorana* L. against *P*. *aeruginosa* ATCC 25922. b) EO *O*. *majorana* L. against *E*. *coli* ATCC 8789. c) EO *O*. *majorana* L. against *S*. *aureus* ATCC 25922. Test substance of essential oil of the *O*. *majorana* L. (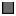), BHI with 2% of DMSO (■) and Amoxiline (□); ***P <0.001 statistically significant in relation to the negative control; # p <0.001 statistically significant in relation to the positive control.

The minimum inhibitory concentration (MIC) and minimum bactericidal concentration (MBC) that were identified for *O*. *majorana* L. EO can be verified in Fig 2. The results show that gram-negative bacteria were more sensitive to EO presenting MIC = 31.25 μg.mL^-1^, compared to the negative control. The MBC for *E*. *coli* was at the concentration of 500 μg.mL^-1^ and for *P*. *aeruginona* was at the concentration of 1000 μg.mL^-1^ in relation to the negative control (amoxicillin). While the *S*. *aureus* bacterium did not present MIC, neither MBC.

According to Rosato et al. [68], the antibacterial activity in gram-negative bacteria occurs due to the high percentage of oxygenated monoterpenes present in the EO and consequently the synergism between these components. On the other hand, bacteria can also respond to adverse conditions in a transient way, through so-called stress tolerance responses. Bacterial stress tolerance responses include structural and physiological modifications in the cell, and complex genetic regulatory machines mediate them [69].

In the study by Duru et al. [70] pulegone showed high antimicrobial activity, particularly against *Candida albicans* and *Salmonella typhimurium*. Pulegone is classified as a monoterpene, in the same way as carvone. It can be obtained from a variety of plants [71, 72]. Menthone is a common volatile compound in Lamiaceae, which may also be active against a large number of bacteria, such as *E*. *coli* and *Enterococcus faecalis* [73, 74]. Some studies have argued that monoterpenes can cross cell membranes and interact with intracellular sites critical for antibacterial activity [75].

However, reports of non-adaptation or cross-adaptation of bacteria to sub lethal concentrations of major constituents of essential oils has also been reported [76]. Cross-resistance can occur when different antimicrobial agents attack the same target in the cell, reach common route of access to the respective targets or initiate a common pathway for cell death, the resistance mechanism is the same for more than one antibacterial agent [77].

### Essential antioxidant activity of EO of *O*. *majorana* L. by DPPH radical capture method

The concentrations of EO obtained IC_50_ = 16.83 μg.mL^-1^ according to Table 4 and was compared with ascorbic acid (vitamin C) in Table 5 which showed IC_50_ = 16.71 μg.mL^-1^. The absorbance of EO was Y = 0.0196 x 17.0078. coefficient (R^2^) = 0.9600.

**Table 4:** Shows the percentage of the antioxidant activity of *O*. *majorana* L. EO.

Concentrations (μg.mL^-1^)	AA (%)
7.81	16.95^a a^
15.62	17.15^b^
31.25	17.48^b^
62.5	18.9^c^
125	19.28^d^
250	21.85^e^

Different letters indicate that there was significant difference of Tunkey test (p≤0.05).

**Table 5:** Shows the percentage of antioxidant activity of ascorbic acid (vitamin C) in different concentrations.

Concentrations (μg.mL^-1^)	AA (%)
7.81	18.57%^a^
15.62	30%^b^
31.25	99.93%^c^
62.5	99.99%^d^
125	99.99%^d^
250	99.99%^d^

Different letters indicate that there was a significant difference (p≤0.05).

Many antioxidants derived from natural products demonstrate neuroprotective activity in vitro and/or in vivo models such as flavonoid phenolic compounds [78].

The percentage of antioxidant activity of the essential oil showed a high IC_50_ = 16.83 μg.mL^-1^, whereas ascorbic acid presented 16.71 μg.mL^-1^ [79]. According to Rodrigues [80] the higher the consumption of DPPH for a smaller sample will be its IC_50_ and the greater its antioxidant capacity.

According to Beatović et al. [81], the antioxidant capacity of EO is related to its main compounds. However, this study did not present antioxidant activity. The importance concerning the performance of antioxidants depends on the factors types of free radicals formed; where and how these radicals are generated; analysis and methods for identifying damage, and ideal doses for protection [82].

### Toxicity to *A*. *salina* L. EO of *O*. *majorana* L.

*A*. *salina* L. is a microcrustacean used in fish feed, and it is widely used in toxicological studies because of the low cost and easy cultivation. Several studies have attempted to correlate toxicity on *A*. *salina* L. with antifungal, virucidal, antimicrobial, trypanosomicidal and parasiticidal activities. Lethality assays are performed in toxicological tests and the median lethal concentration (LC_50_), which indicates death in half of a sample, can be obtained [83].

Table 6 shows the percentage of cytotoxic activity of *O*. *majorana* L. EO and the mean mortality readings after the 24 h period are expressed. The oil concentrations presented LC_50_ of 172.6 μg.mL^-1^, the coefficient of determination (R^2^) of 0.883 and *X*^*2*^ of 1.915.

**Table 6:** Shows the percentage of cytotoxic activity of *O*. *majorana* L. essential oil at different concentrations.

Concentrations (μg.mL^-1^)	Mortality (%)
Control negative	0.0%a
50	9.37%b
100	48.48%c
250	67.39%d
500	75.40%e
750	80.26%f
1000	83.51%g
LC_50_ (K_2_Cr_2_O_7_)	172.60 μg.mL^-1^

Different letters indicate a significant difference between the concentrations (p <0.05).

According to Nguta et al. [84], both organic extracts and aqueous extracts with LC_50_ values of less than 100 μg.mL^-1^ show high toxicity, LC_50_ between 100 and 500 μg.mL^-1^ exhibited moderate toxicity, LC_50_ between 500 and 1000 μg.mL^-1^ presented low toxicity and LC_50_ above 1000 μg.mL^-1^ are considered to be non-toxic (non-toxic).

The lethal concentration of mortality against the *A*. *salina* L. larvae of this assay showed moderate cytotoxic activity. In order to evaluate the cytotoxicity of a given sample, it is possible to elucidate the cytotoxic effect of the cytotoxic mechanism and the mechanism of action of different compounds during their interaction with the tissues [85].

## Conclusions

The results of the present study demonstrated that OE obtained from dry leaves of *O*. *majorana* L. showed good larvicidal activity against *A*. *aegypti* L. larvae with mortality from the concentration of 20 μg.mL^-1^ in 48 h. In relation to the chemical analysis, it presented a mixture of monoterpenes, with the major constituent being pulegone (57.05%), followed by the other constituents verbenone (16.92%), trans-menthone (8.57%), cis-menthone), piperitone (2.83%), 3-octanol (2.35%) and isopulegol (1.47%). The oil showed satisfactory antimicrobial activity against *P*. *aeruginosa* and *E*. *coli* bacteria. In addition, despite the lack of antioxidant activity by the DPPH radical capture method, the oil showed moderate cytotoxic activity against *A*. *salina* L. In summary, these results provide subsidies for future EO *O*. *majorana* L. studies in order to enhance the use of organic compounds with larvicidal activity against the *A*. *aegypti* L. mosquito, as well as the importance of the study of bioactive plant products that do not pollute the environment and that do not cause harm to human health.

## Supporting information

S1 File Mass spectrum of *O*. *majorana* essential oil, obtained by GC-MS as compared to the spectrum of the equipment library NIST05 e WILEY'S and Adams (2017). (DOCX)

## References

[1] paho.org [internet] World Health Organization: Neglected, tropical and vector borne disease—dengue. c2017 [cited 2018 dec 28]. http://www.paho.org/hq/index.php?option=com_topics&view=article&id=1&Itemid=40734.

[2] Bhatt S, Gething PW, Brady OL, Messina JP, Farlow AW, Moyes CL, et al The global distribution and burden of dengue. Nature. 25 of 4 of 2013; 496 (7446): 504–7. 10.1038/nature12060 23563266 PMC3651993

[3] Gonçalves NVS, Rebêlo JMM. [Epidemiological aspects of dengue in the municipality of São Luís, Maranhão, Brazil, 1997–2002]. Cadernos de Saúde Pública. 2004; 20: 1424–1431. Português. 15486688 10.1590/s0102-311x2004000500039

[4] Barbosa IR, Araújo LF, Carlota FC, Araújo RS, Maciel IJ. [Epidemiology of dengue fever in the State of Rio Grande do Norte, Brazil, 2000 to 2009]. Epidemiol Serv Saúde. 2012; 21: 149–57. Português.

[5] Linnaeus C. Reise nach Palästina in den Jahren van. Salvii, Stockholm 1762 1–619.

[6] Vasconcelos PFC. Doença pelo vírus Zika: um novo problema emergente nas Américas? Rev. PanAmaz. Saúde. 2015; 6: 9–10. Português.

[7] Lima-Camara T. N. [Emerging arboviroses and new challenges for public health in Brazil]. Ver Saúde Pública. 7 of 3 of 2016; 50 (36): 1–7. 10.1590/S1518-8787.2016050006791 PMC4936892 27355468

[8] Zara A. L.; dos Santos S. M.; Oliveira-Fernandes E. S.; Carvalho R. G.; Coelho G. E. [Strategies for controlling Aedes aegypti: a review]. Epidemiol Serv Saúde. 2016; 25 (2): 391–404. Português. 27869956 10.5123/S1679-49742016000200017

[9] Araújo VEM, Bezerra JMT, Frederico FA, Passos VMA, Carneiro M. [Increased burden of dengue in Brazil and federated units, 2000 and 2015: analysis of the *Global Burden of Disease Study* 2015]. Revista Brasileira de Epidemiologia. 2017; 20: 205–216. Português. 10.1590/1980-5497201700050017 28658384

[10] Wang X, Li JL, Xing HJ, Xu SW. Review of the toxicology of atrazine and chlorpyrifos onfish. Journal Northeast Agric Univ, 2011, (18): 88–92.

[11] Mohammed MP, Penmethsa KK. Assessment of pesticide residues in surface waters of Godavari delta, India. Journal Mater Environ Sci, 2014, (5): 33–6.

[12] Djogbénou L. Vector controls methods against malaria and vector resistance to insecticides in Africa. Med Trop, 2009, (69): 160–4. 19545042

[13] El Ouali Lalami A, El-Akhal F, El Amri N, Maniar S, Faraj C. State resistance of the mosquito *Culex pipiens* towards temephos *central Morocco*. Bull Soc Pathol Exot Ses Fil, 2014, (107): 194–8. 10.1007/s13149-014-0361-x 24827876

[14] Azizi A, Yan F, Honermeier B. Herbage yield, essential oil content and composition of three oregano (*Origanum vulgare* L.) populations as affected by soil moisture regimes and nitrogen supply. Ind Crops Prod 2009, (29): 554–61.

[15] Abdel-Massih R. M. and Abraham A., “Extracts of *Rosmarinus officinalis*, *Rheum rhaponticum*, and *Origanum majorana* exhibit significant anti-staphylococcal activity,” *International Journal of Pharmaceutical Sciences and Research*, vol. 5, pp. 819–828, 2014.

[16] Busatta C., Vidal R. S., Popiolski A. S., et al, “Application of *Origanum majorana* L. essential oil as an antimicrobial agent in sausage,” *Food Microbiology*, vol. 25, no. 1, pp. 207–211, 2008. 17993397 10.1016/j.fm.2007.07.003

[17] Prakash B., Singh P., Kedia A., and Dubey N. K., “Assessment of some essential oils as food preservatives based on antifungal, antiaflatoxin, antioxidant activities and *in vivo* efficacy in food system,” *Food Research International*, vol. 49, no. 1, pp. 201–208, 2012.

[18] Linnaeus C. Species Plantarum. 1753. 590 p.

[19] MARTINOV II. Tekhno-Botanicheskīĭ Slovarʹ: na latinskom i rossīĭskom iazykakh. Sanktpeterburgie. 1820.

[20] Mitropoulou G, Fitsiou E, Stavropoulou E, Papavassilopoulou E, Vamvakias M, Pappa A, et al Composition, antimicrobial, antioxidant, and antiproliferative activity of *Origanum dictamnus* (dittany) essential oil. Microbial Ecology in Health and Disease. 6 of 5 of 2015; 26: (1–9): 26543. 25952773 10.3402/mehd.v26.26543 PMC4424236

[21] Bina F, Rahimi R. Sweet Marjoram: A Review of Ethnopharmacology, Phytochemistry, and Biological Activities. J Evid Based Int Med. 2016; 22 (1): 175–185. 10.1177/2156587216650793 . 27231340 PMC5871212

[22] Al Dhaheri Y., Attoub S., Arafat K., et al, “Anti-metastatic and anti-tumor growth effects of *Origanum majorana* on highly metastatic human breast cancer cells: inhibition of NFκB signaling and reduction of nitric oxide production,” *PLoS ONE*, vol. 8, no. 7, Article IDe68808, 2013. 10.1371/journal.pone.0068808 PMC3707896 23874773

[23] Rao S., Timsina B., and Nadumane V. K., “Evaluation of the anticancer potentials of *Origanum marjorana* on fibrosarcoma (HT-1080) cell line,” *Asian Pacific Journal of Tropical Disease*, vol. 4, no. 1, pp. S389–S394, 2014.

[24] Ramadan G., El-Beih N.M., and Zahra M. M., “Egyptian sweet marjoram leaves protect against genotoxicity, immunosuppression and other complications induced by cyclophosphamide in albino rats,” *British Journal of Nutrition*, vol. 108, no. 6, pp. 1059–1068, 2012. 22172207 10.1017/S0007114511006210

[25] Khan J.A., Jalal J. A., Ioanndes C., and Moselhy S. S., “Impact of aqueous doash extract on urinary mutagenicity in rats exposed to heterocyclic amines,” *Toxicology and Industrial Health*, vol. 29, no. 2, pp. 142–148, 2013. 22173956 10.1177/0748233711427053

[26] Vági E., Simándi B., Suhajda A., and Hethelyi E., “Essential oil composition and antimicrobial activity of *Origanum majorana* L. extracts obtained with ethyl alcohol and supercritical carbon dioxide,” *Food Research International*, vol. 38, no. 1, pp. 51–57, 2005.

[27] Ebrahimi M., Khosravi-Darani K., 2013 Essential oils as natural food preservatives: antimicrobial and antioxidant applications In: Doughari James (Ed.), Antimicrobials from Nature: Effective Control Agents for Drug Resistant Pathogens. Transworld Research Network, Kerala, India.

[28] Bassalé J.R.N., Juliani H.R., (2012). Essencial oils in combination and their antimicrobial properties. Molecules, 3989–4006. 22469594 10.3390/molecules17043989 PMC6268925

[29] Dussault D, Vu KD, Lacroix M. *In vitro* evaluation of antimicrobial activities of various comercial essential oils, oleoresin and pure compounds against food pathogens and application in ham. Meat Science. 2014; 96 (1): 514–520. 10.1016/j.meatsci.2013.08.015 ;. 24012976

[30] Pereira ÁIS, Pereira AGS, Sobrinho OPL, Cantanhede EKP, Siqueira LFS. [Antimicrobial activity in the control of larvae of the mosquito *Aedes aegypti*: Homogenization of the essential oils of linalool and eugenol]. Educ. quím. 2014; 25 (4): 446–449. 10.1016/S0187-893X(14)70065-5 Português.

[31] Anvisa. National Health Surveillance Agency. [Brazilian Pharmacopoeia], 5a ed.; Fiocruz: Brasília, Brasil 2010, pp. 1–545. Português.

[32] Adams RP. Identification of Essential Oil Components by Gas Chromatography/ Mass Spectrometry, 4.1 ed. Biology Department: Baylor Universit, 2017.

[33] World Health Organization (WHO) [internet] Geneva: Guidelines for Laboratory and Field Testing of Mosquito Larvicides. 2005c –[cited 4 december 2018]. http://apps.who.int/iris/bitstream/10665/69101/1/WHO_CDS_WHOPES_GCDPP_2005.13.pdf.

[34] Clinical and Laboratory Standards Institute (CLSI) [internet] Pennsylvania: Standards for Antimicrobial Susceptibility Testing supplement M100. 2018 –[cited 5 january 2019]. https://clsi.org/media/1930/m100ed28_sample.pdf.

[35] Sousa CMM, Silva HR, Junior GMV, Ayres MCC, Costa CLS, Araújo DS, et al [Total phenols and antioxidant activity of five medicinal plants]. Química Nova. 2007; 30 (2): 351–355. Português.

[36] Lopes-Lutz D, Alviano DS, Alviano CS, Kolodziejczyk PP. Screening of chemical composition, antimicrobial and antioxidant activities of Artemisia essential oils. Phytochemistry. 2008; 69 (8): 1732–8. 10.1016/j.phytochem.2008.02.014 18417176

[37] Andrade MA, Cardoso GM, Batista RL, Mallet TCA, Machado FMS. [Essential Oils of *Cymbopogon Nardus*, *Cinnamomum Zeylanicum* and *Zingiber Officinale*: composition, antioxidant and antibacterial activities]. J Agron Sci. 2012; 43 (2): 399–408. Português.

[38] Araújo MGF, Cunha RW, Veneziani RCS. Preliminary phytochemical study and toxicological bioassay against larvae of *Artemia salina* Leach. of extract obtained from fruits of *Solanum lycocarpum* A. St.-Hill (Solanaceae). J Basic Appl Pharm Sci [intenet]. 1 5 2010 [cited on 12 february 2019]; 31 (2): 205–209. https://repositorio.unesp.br/handle/11449/71676.

[39] Lôbo KMS, Athayde ACR, Silva AMA, Rodrigues FFG, Lôbo IS, Bezerra DAC, et al Evaluation of the antibacterial activity and phytochemical prospection of *Solanum paniculatum* Lam. And *Operculina hamiltonii* (G. Don) D. F. Austin e Staples, from the semi-arid region of Paraíba. Braz J Med Plants. 2010; 12 (2): 227–233.

[40] Araújo TAS, Castro VTNA, Solon LGS, Silva GA, Almeida MG, Costa JGM, et al Does rainfall affect the antioxidant capacity and production of phenolic compounds of an important medicinal species?. Ind Crops Prod. 15 12 2015; 76: 550–556, 2015. 10.1016/j.indcrop.2015.07.008

[41] Estell RE, Fredrickson EL, James DK. Effect of light intensity and wavelength on concentration of plant secondary metabolites in the leaves of Flourensia cernua. Biochem Syst Ecol. 2016 4; 65: 108–114. 10.1016/j.bse.2016.02.019

[42] Lima TC, Silva TK, Silva FL, Barbosa JMJ, Marques MO, Santos RL, et al Atividade larvicida do óleo essencial de *Mentha* × *villosa* Hudson, rotundifolona e derivados. Chemosphere. 2014 6; 104: 37–43. 10.1016/j.chemosphere.2013.10.035 24275151

[43] Souza AVV, Santos US, Carvalho JRS, Barbosa BHS, Canuto KM, Rodrigues THS. Chemical Composition of Lippia schaueriana Essential Leaf Fight Mart. Collected in the Caatinga Area. Molecules. [preprint]. 2018. [posted 2018 september 27; accepted 2018 july 6; reiceved 2018 may 23]: [6p.] https://ainfo.cnptia.embrapa.br/digital/bitstream/item/183650/1/Lippia-schaueriana-molecules-23-02480-3.pdf. 10.3390/molecules23102480 PMC6222887 30262744

[44] Macêdo DG, Souza MA, Morais-Braga MFB, Coutinho HDM, Santos ATL, Cruz RP, et al Effect of seasonality on chemical profile and antifungal activity of essential oil isolated from leaves *Psidium salutare* (Kunth) O. Berg. Peer J. 1 11 2018; 6 (1–19). 10.7717/peerj.5476 30402343 PMC6215697

[45] Stoppacher N, Kluger B, Zeilinger S, Krska R, Schuhmacher R. Identification and profiling of volatile metabolites of the biocontrol fungus Trichoderma atroviride by HS-SPME-GC-MS. J Microbiol Methods. 2010; 81 (2): 187–193. 10.1016/j.mimet.2010.03.011 . 20302890

[46] Kessler A, Baldwin IT. Defensive function of herbivore-induced plant volatile emissions in nature. Science. 2001; 291 (5511): 2141–4. 10.1126/science.291.5511.2141 11251117

[47] Takshak S, Agrawal SB. The role of supplemental ultraviolet-B radiation in altering the metabolite profile, essential oil content and composition, and free radical scavenging activities of Coleus forskohlii, an indigenous medicinal plant. Environ Sci Pollut Res Inst. 2016; 23 (8): 7324–37. 10.1007/s11356-015-5965-6 26681329

[48] Bitu V, Costa J, Rogrigues F, Colares A, Coutinho H, Botelho M, et al Effect of collection time on composition of essential oil of *LippiagracilisSchauer* (Verbenaceae) growing in Northeast Brazil. Journal of Essential Oil Bearing Plants. [preprint]. 2015 [posted 2016 jul 09; received 2012 aug 08; cited 2019 jan 21]: [647–653 p.] https://www.tandfonline.com/doi/abs/10.1080/0972060X.2014.935043

[49] Komalamisra N, Trongtokit Y, Rongsriyam Y, Apiwathnasorn C. Screening for larvicidal activity in some Thai plants against four mosquito vector species. Southeast Asian J Trop Med Public Health. 2005; 36 (6): 1412–22. . 16610643

[50] Magalhães LAM, Lima MP, Marques MOM, Facanali R, Pinto ACS, Tadei WP. Chemical composition and larvicidal activity against *Aedes aegypti* larvae of essential oils from four Guarea species. Molecules. 2010; 15 (8): 5734–5741. 10.3390/molecules15085734 . 20724962 PMC6257719

[51] Dias CN, Moraes DFC. Essential oils and their compounds as *Aedes aegypti* L. (Diptera: Culicidae) larvicides: review. Parasitol Res. 2014; 113 (2): 565–92. 10.1007/s00436-013-3687-6 24265058

[52] Cantrell CL, Pridgeon JW, Fronczek FR, Becnel JJ. Structure activity relationship studies on derivatives of Eudesmanolides from Inula helenium as toxicants against *Aedes aegypti* larvae and adults. Chem Biodivers. 2010; 7 (7): 1681–97. 10.1002/cbdv.201000031 20658657

[53] Souza TM, Cunha AP, Farias DF, Machado LK, Morais SM, Ricardo NMPS, et al Insecticidal activity against *Aedes aegypti* of m-pentadecadienyl-phenol isolated from Myracrodruon urundeuva seeds. Pest Manag Sci. 2012; 68 (10): 1380–4. 10.1002/ps.3316 22689540

[54] Kumar P, Mishra S, Malik AS. Insecticidal properties of *Mentha* species: A review. Ind Crops Prod. 2011 7; 34: 802–817. 10.1016/j.indcrop.2011.02.019

[55] Omolo MO, Okinyo D, Ndiege IO, Lwande W, Hassanali A. Fumigant toxicity of the essential oils of some African plants against *Anopheles gambiae* sensu stricto. Phytomedicine. 2005; 12 (3):241–6. 10.1016/j.phymed.2003.10.004 15830848

[56] El-Akhal F, Greche H, Ouazzani CF, Guemmouh R, El Ouali Lalami A. Chemical composition and larvicidal activity of Culex pipiens essential oil of Thymus vulgaris grown in Morocco. JMater Environ Sci 2015;1:214–9.

[57] El-Akhal F, El Ouali Lalami A, Ez Zoubi Y, Greche H, Guemmouh R. Chemical composition and larvicidal activity of essential oil of Origanum majorana (Lamiaceae) cultivated in Morocco against Culex pipiens (Diptera: Culicidae). Asian Pac J Trop Biomed 2014;4:746–50.

[58] Szczepanik M, Zawitowska B, Szumny A. Insecticidal activities of Thymus vulgaris essential oil and its components (thymol and carvacrol) against larvae of lesser mealworm, Alphitobius diaperinus Panzer (Coleoptera Tenebrionidae). Allelopathy J 2012;30:129–42.

[59] Cavalcanti ESB, de Morais SM, Lima MAA, Santana EWP. Larvicidal activity of essential oils from Brazilian plants against Aedes aegypti L. Mem Inst Oswaldo Cruz 2004;99:541–4. 15543421 10.1590/s0074-02762004000500015

[60] Pavela R, Kaffková K, Kumsta M. Chemical composition and larvicidal activity of essential oils from different *Mentha* L. and *Pulegium* species against *Culex quinquefasciatus* Say (Diptera: Culicidae). Plant Protect. Sci. 2014; (50): 36–42.

[61] Koliopoulos G., Pitarokili D., Kioulos E., Michaelakis A., Tzakou O. (2010): Chemical composition and larvicidal evaluation of Mentha, Salvia and Melissa essential oils against the West Nile virus mosquito Culex pipiens. Parasitology Research, 107: 327–375. 20405142 10.1007/s00436-010-1865-3

[62] Lucia A, Zerba E, Masuh H. Knockdown and larvicidal activity of six monoterpenes against Aedes aegypti (Diptera: Culicidae) and their structure-activity relationships. Parasitol Research. 2013, (112): 4267–4272. 10.1007/s00436-013-3618-6 24100604

[63] El-Akhal F, Guemmouh R, Maniar S, Taghzouti K, Lalami A E O, Larvicidal Activity of Essential Oils of Thymus Vulgaris and Origanum Majorana (Lamiaceae) Against of the Malaria Vector Anopheles Labranchiae (Diptera: Culicidae). 2016, International Journal of Pharmacy and Pharmaceutical Sciences. (8); 372–376, issue 3.

[64] Pavela R, Sedlák P. Post-application temperature as a factor influencing the insecticidal activity of essential oil from Thymus vulgaris. Industrial Crops e Products. 2018, (113); 46–49.

[65] Pavela R. History, presence and perspective of using plant extracts as commercial botanical insecticides and farm products for protection against insects–a review. Plant Prot. Sci. 2016, (52): 229–241.

[66] Satyan RS, Malarvannan S, Eganathan P, Rajalakshmi S, Parida A. Growth inhibitory activity of fatty acid methyl esters in the whole seed oil of *Madagascar periwinkle* (Apocyanaceae) against *Helicoverpa armigera* (Lepidoptera: Noctuidae). J Econ Entomol. 2009; 102 (3): 1197–202. . 19610438 10.1603/029.102.0344

[67] Chere JMC, Dar MA, Pandit RS. Evaluation of Some Essential Oils against the Larvae of House Fly, Musca domestica by Using Residual Film Method. Biotechnol Microb. 16 4 2018; 9 (1): 555752.

[68] Rosato A, Carocci A, Catalano A, Clodoveo ML, Franchini C, Corbo F, et al Elucidation of the synergistic action of Mentha Piperita essential oil with common antimicrobials. PLoS One. 1 1 2018; 13 (8):e0200902 10.1371/journal.pone.0200902 . 30067803 PMC6070247

[69] Alvarez-Ordóñez A.; Broussolle V.; Colin P.; Nguyen-The C.; PRIETO M. The adaptive response of bacterial food-borne pathogens in the environment, host and food: implications for food safety. Int J Food Microbiol. 20 11 2015; 213: 99–109. 10.1016/j.ijfoodmicro.2015.06.004 26116419

[70] Duru ME, Ozturk M, Ugur AU, Ceylan O. The constituents of essential oil and in vitro antimicrobial activity of *Micromeria cilicica* from Turkey. J Ethnopharmacol. 94 (1): 43–48. 10.1016/j.jep.2004.03.053 15261961

[71] Siano F, Catalfamo M, Cautela D, Servillo L, Castaldo D. Analysis of pulegone and its enanthiomeric distribution in mint-flavoured food products. Food Addit Contam. 2005; 22 (3): 197–203. 10.1080/02652030500041581 16019787

[72] Burt S. Essential oils: their antibacterial properties and potential applications in foods-a review. Int J Food Microbiol. 1 8 2004; 94 (3): 223–253. 10.1016/j.ijfoodmicro.2004.03.022 15246235

[73] Singh R, Shushni MAM, Belkheir A. Antibacterial and antioxidant activities of *Mentha piperita* L. Arab J Chem. 5 2015; 8 (3): 322–328. 10.1016/j.arabjc.2011.01.019

[74] Thosar N, Basak S, Bahadure RN, Rajurkar M. Antimicrobial efficacy of five essential oils against oral pathogens: an in vitro study. Eur J Dent. 9 2013; 7 (1): 71–77. 10.4103/1305-7456.119078 24966732 PMC4054083

[75] Turchi B, Mancini S, Pistelli L, Najar B, Cerri D, Fratini F. Sub-inhibitory stress with essential oil affects enterotoxins production and essential oil susceptibility in Staphylococcus aureus. Nat Prod Res. Mach 2017; 32 (6): 682–688. 10.1080/14786419.2017.1338284 28595460

[76] Santos JMP. [Adaptation and Cross-Adaptation of Listeria Spp. Essential Oils from Condensed Plants and Acid Stress]. [dissertação]. Programa de Pós-Graduação em Plantas Medicinais, Aromáticas e Condimentares: Universidade de Lavras; 2018. Português.

[77] Chapman JS. Desinfectant resistence mechanisms, cross-resistance, and coresistance. International Int Biodeterior Biodegradation. 6 2003; 51 (1): 271–276. 10.1016/S0964-8305(03)00044-1

[78] Kelsey N. A.; Wilkins H. M.; Linseman D. Nutraceutical Antioxidants as Novel Neuroprotective Agents. Molecules. 3 11 2010; 15 (11): 7792–814. 10.3390/molecules15117792 . 21060289 PMC4697862

[79] Godói AA, Ishikawa BR, porto Ark, Roel RA, Xavier NCP, Yano M. [Evaluation of antioxidant, antibacterial and cytotoxic activity of *Urera aurantiaca*]. Rev Bras Farm [internet]. 8 2011 [cited on 22 january 2019]; 92 (3): [198–202 2011]. http://www.rbfarma.org.br/files/rbf-2011-92-3-19.pdf.

[80] Rodrigues JSQ. [Infusions based on leaves of passifloras of the cerrado: phenolic compounds, antioxidant activity in vitro and sensorial profile]. [dissertação] Universidade de Brasília. 2012. Português.

[81] Beatović D, Krstić-Milošević D, Trifunović S, Šiljegović J, Glamoćlija J, Ristić ME. Chemical composition, antioxidant and antimicrobial activities of the essential oils of twelve cultivars of *Ocimum basilicum* L. grown in Serbia. Rec Nat Prod. [received 2012 augu 24; revised 2013 octo 22; cited 22 january 2019]: [62–75]. https://www.acgpubs.org/doc/201808071238455-RNP-EO_1208-054.pdf.

[82] Singh B, Singh JP, Kaur A, Singh N. Phenolic compounds as beneficial phytochemicals in pomegranate (*Punica granatum* L.) peel: a review. Food Chemistry. 30 9 2018; 261: 75–86. 10.1016/j.foodchem.2018.04.039 29739608

[83] Bednarczuk VO, Verdam MCS, Miguel MD, Miguel OG. [*In vitro* and in vivo tests used in the toxicological screening of natural products]. Visão Acadêmica [preprint]. 2010 [cited 2019 january 23]. https://revistas.ufpr.br/academica/article/view/21366/14087 10.5380/acd.v11i2.21366. Português.

[84] Nguta JM, Mbaria JM, Gakuya DW, Gathumbi PK, Kabasa JD, KIAMA SG. Biological screening of kenya medicinal plants using *Artemia salina* L. (Artemiidae). Pharmacologyonline. [preprint] 2011 [cited 2019 january 23] http://erepository.uonbi.ac.ke/handle/11295/13906.

[85] Marreiro RO, Bandeira MFCL, Almeida MC, Coelho CN, Venâncio GN, Conde NCO. [Evaluation of the cytotoxicity of a buccal mouthwash containing Libidibia iron extract] Braz Res Pediatric Dent Int Clinic. [preprint]. 2014 [cited 2019 january 23] file:///C:/Users/usuario/Downloads/AvaliaodacitotoxicidadedeumenxaguatriobucalcontendoextratodeL.ferrea%20(1).pdf 10.4034/PBOCI.2014.14s3.04. Português.

