# Supplementary material for: Evaluation of larvicidal potential against larvae of Aedes aegypti (Linnaeus, 1762) and of the antimicrobial activity of essential oil obtained from the leaves of Origanum majorana L
Source: PLoS One. 2020 Jul 17;15(7):e0235740. doi: 10.1371/journal.pone.0235740 (PMC7367459; doi:10.1371/journal.pone.0235740)

**Supl 1**. Mass spectrum of *O. majorana* essential oil, obtained by GC-MS as compared to the spectrum of the equipment library NIST05 e WILEY'S and Adams (2017).

**Substance (1)**–α- thujene(tR = 6.658 min).


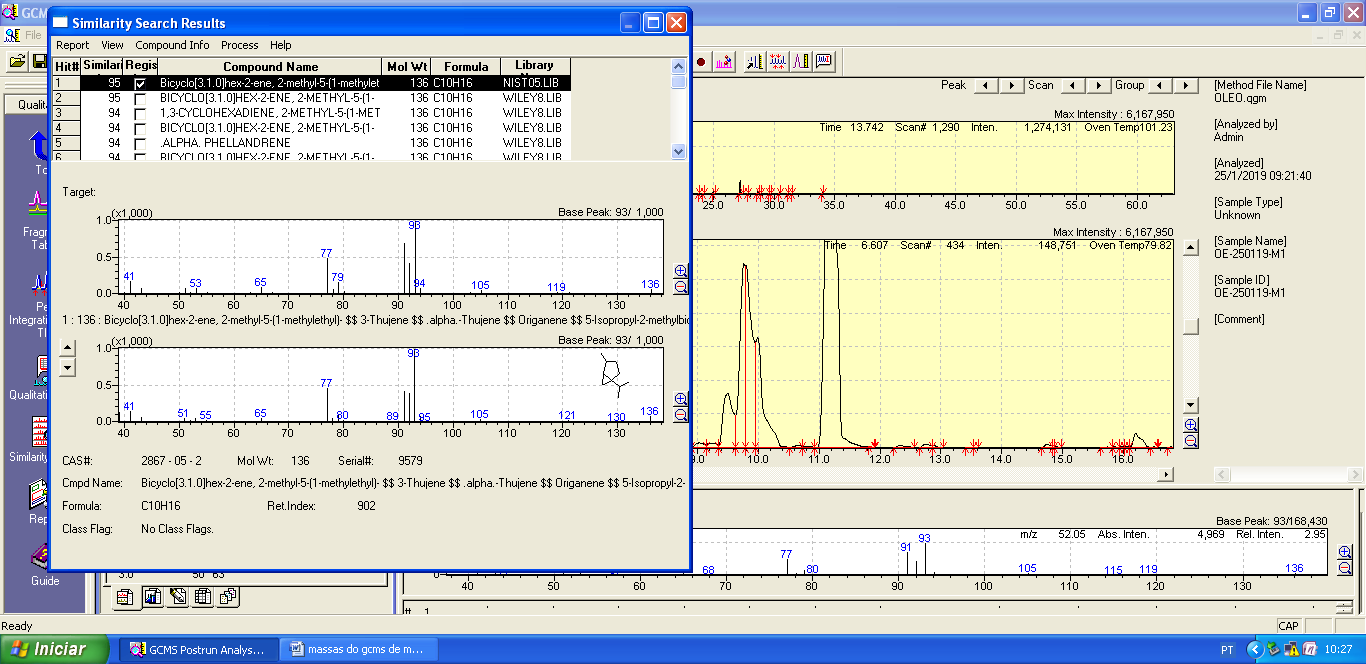


Mass spectrometer for libraries NIST05 e WILEY'S.


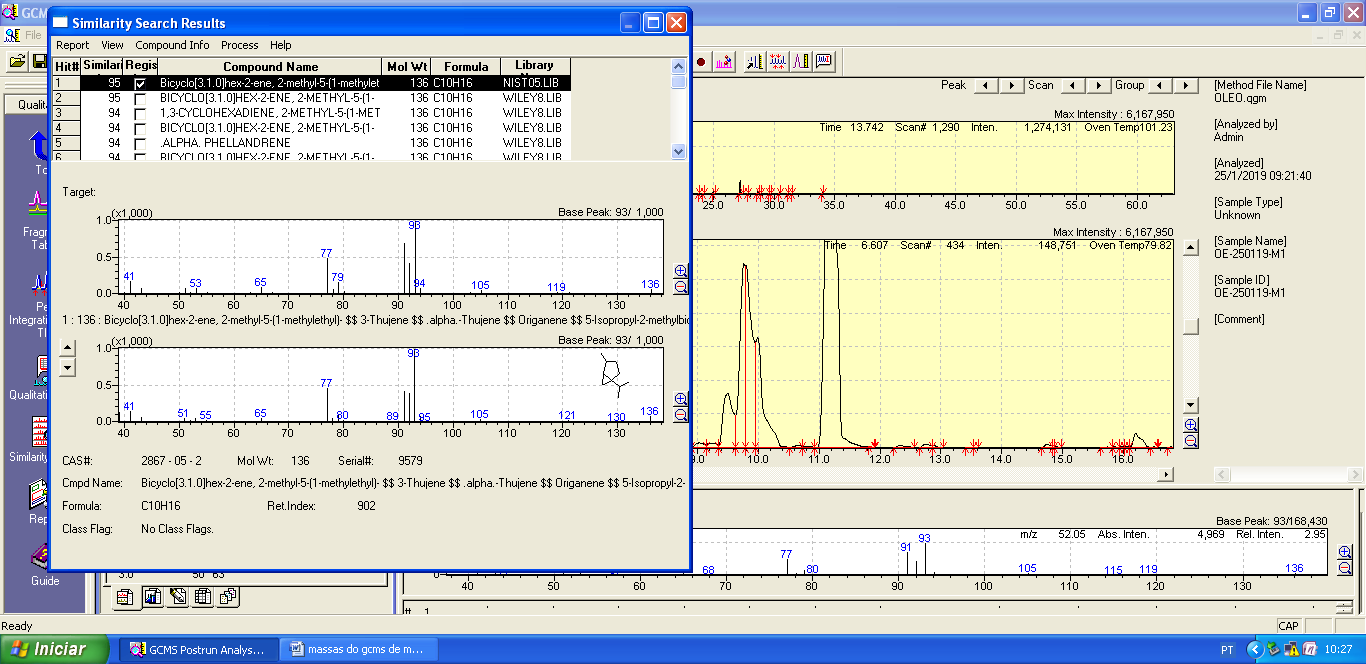


Mass spectrometer of Adams (2017).


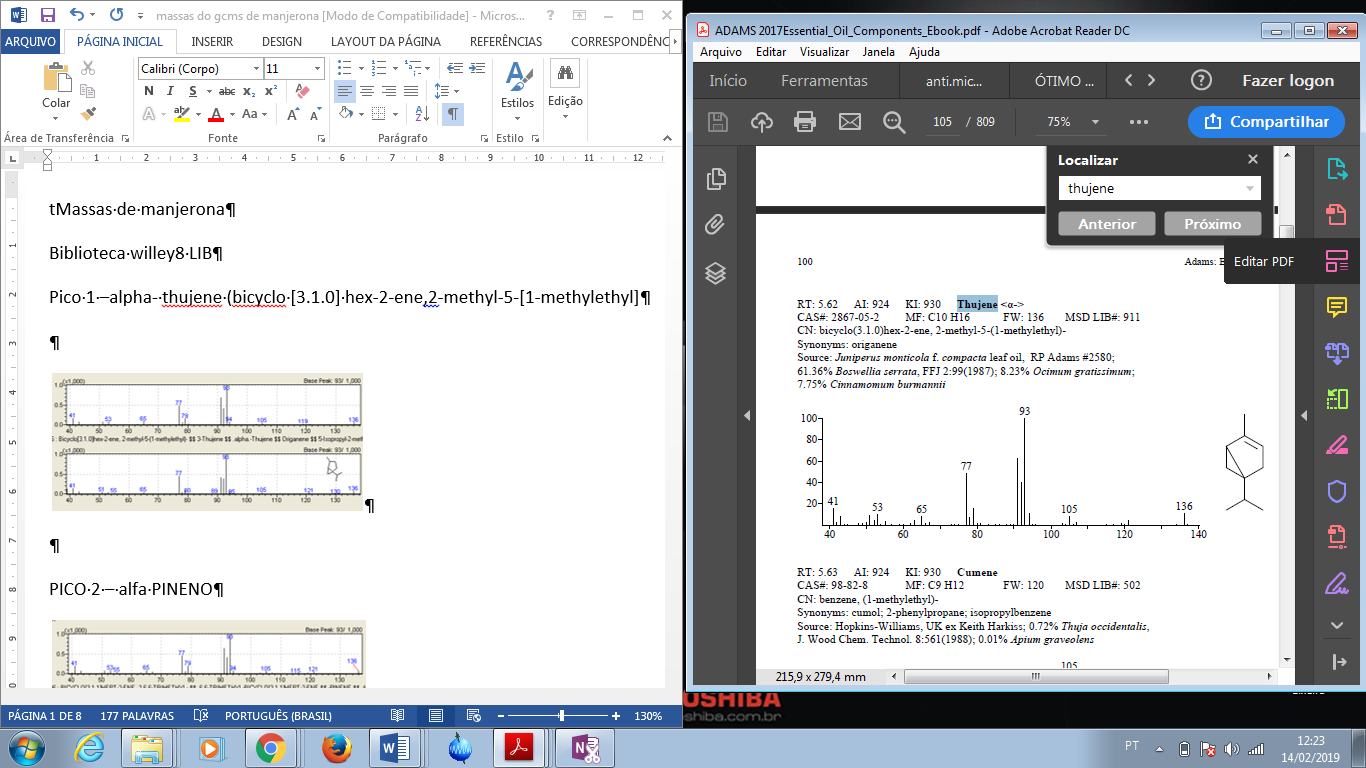


**Substance (2)** – α – pinene(tR = 8.091 min).


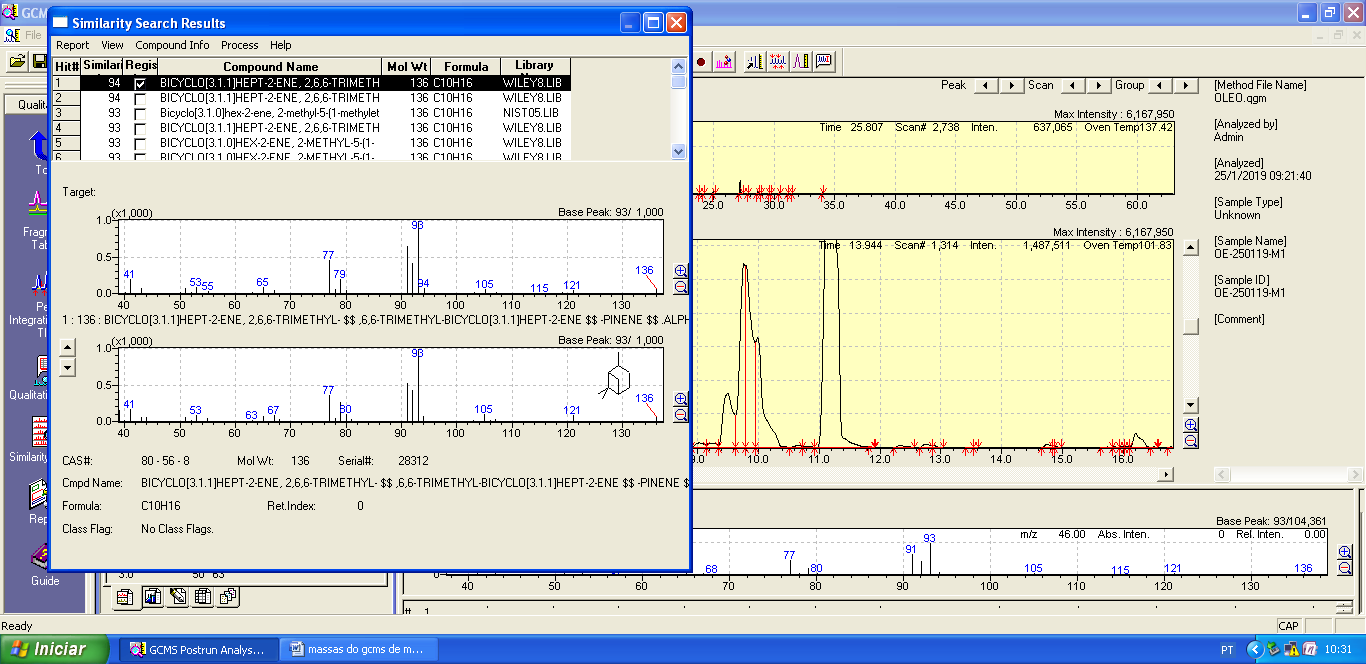


Mass spectrometer for libraries NIST05 e WILEY'S.


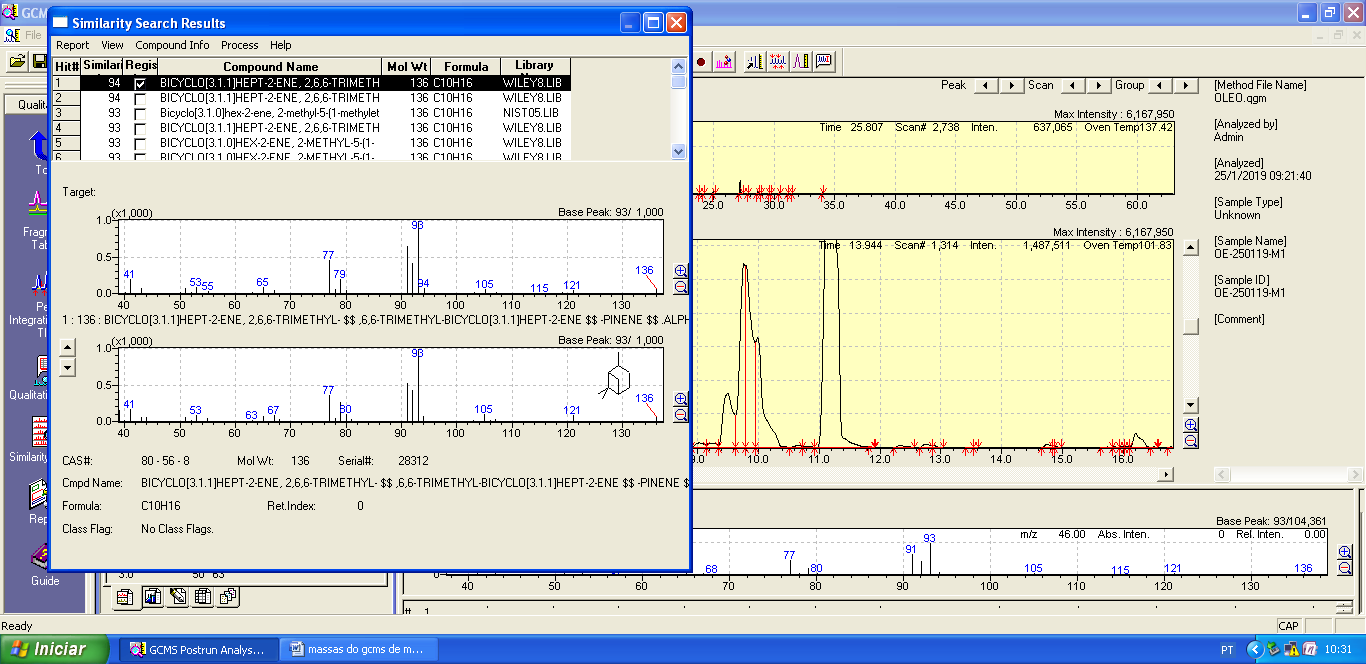


Mass spectrometer of Adams (2017).


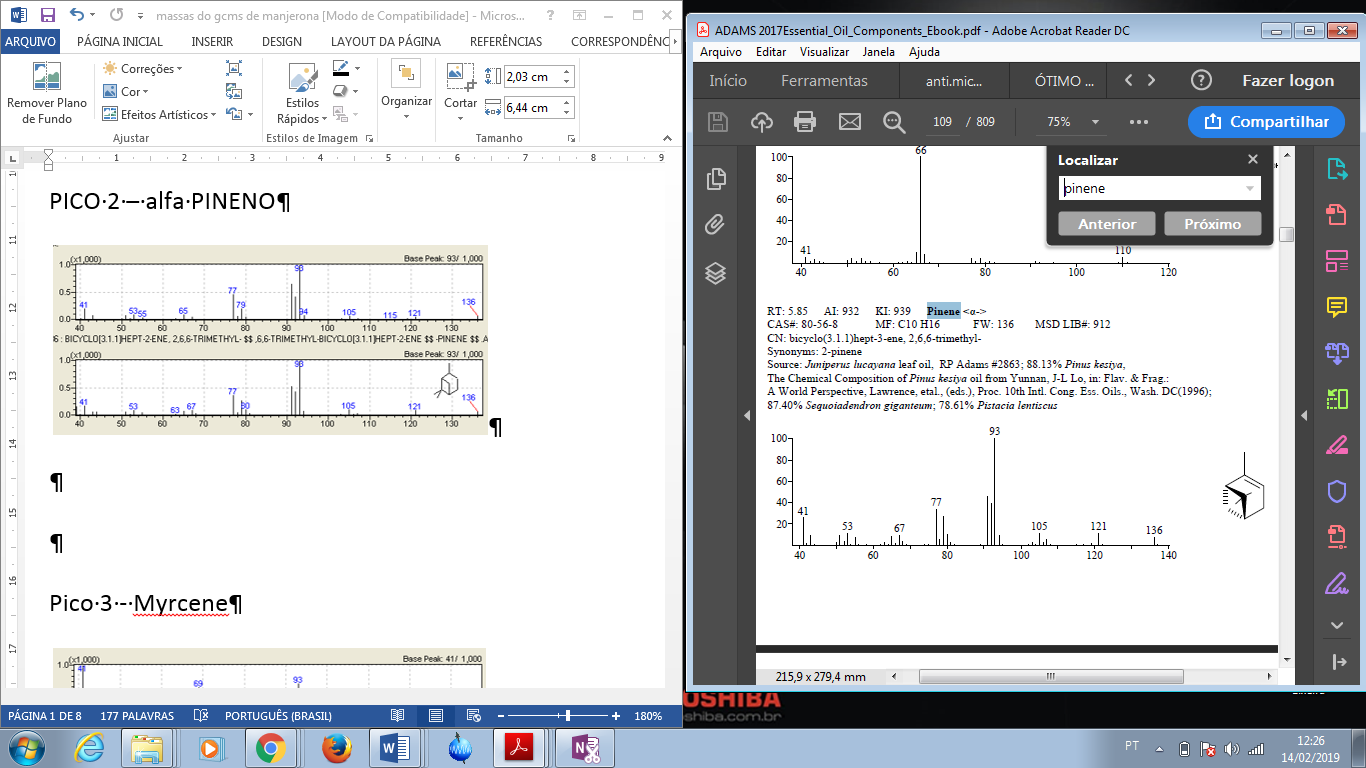


**Substance (3)**–Myrcene(tR = 8.515 min).


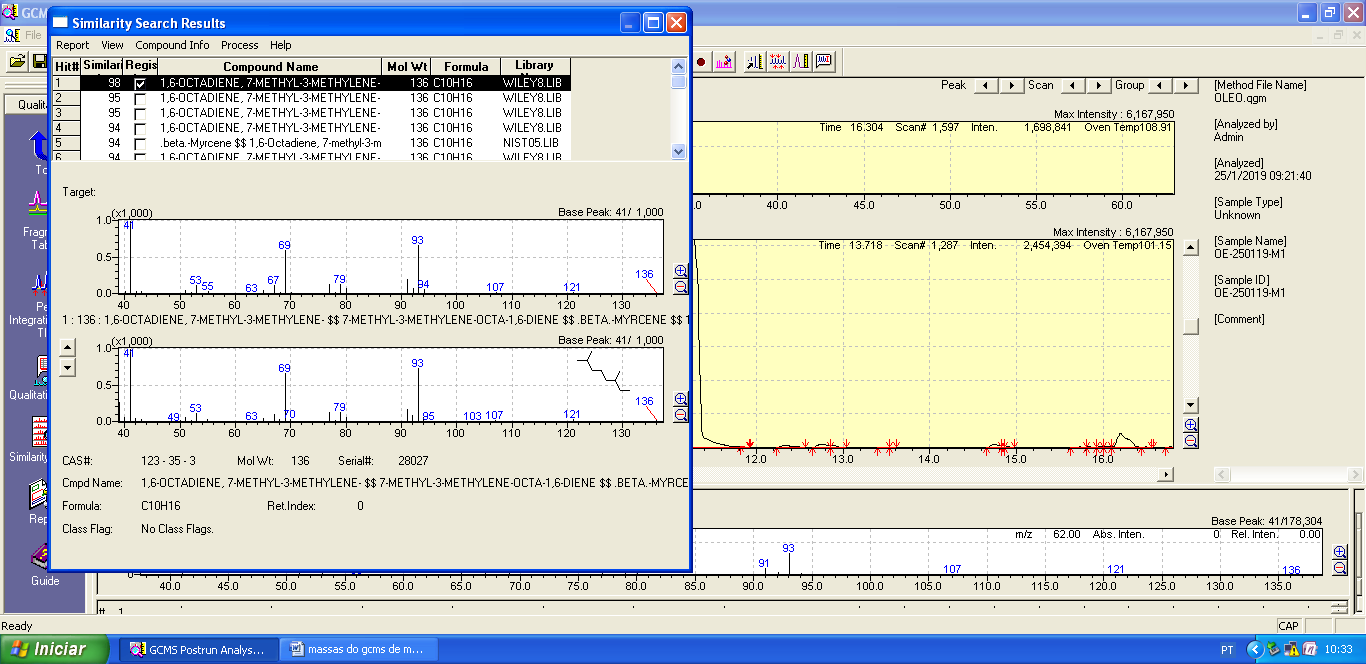


Mass spectrometer for libraries NIST05 e WILEY'S.


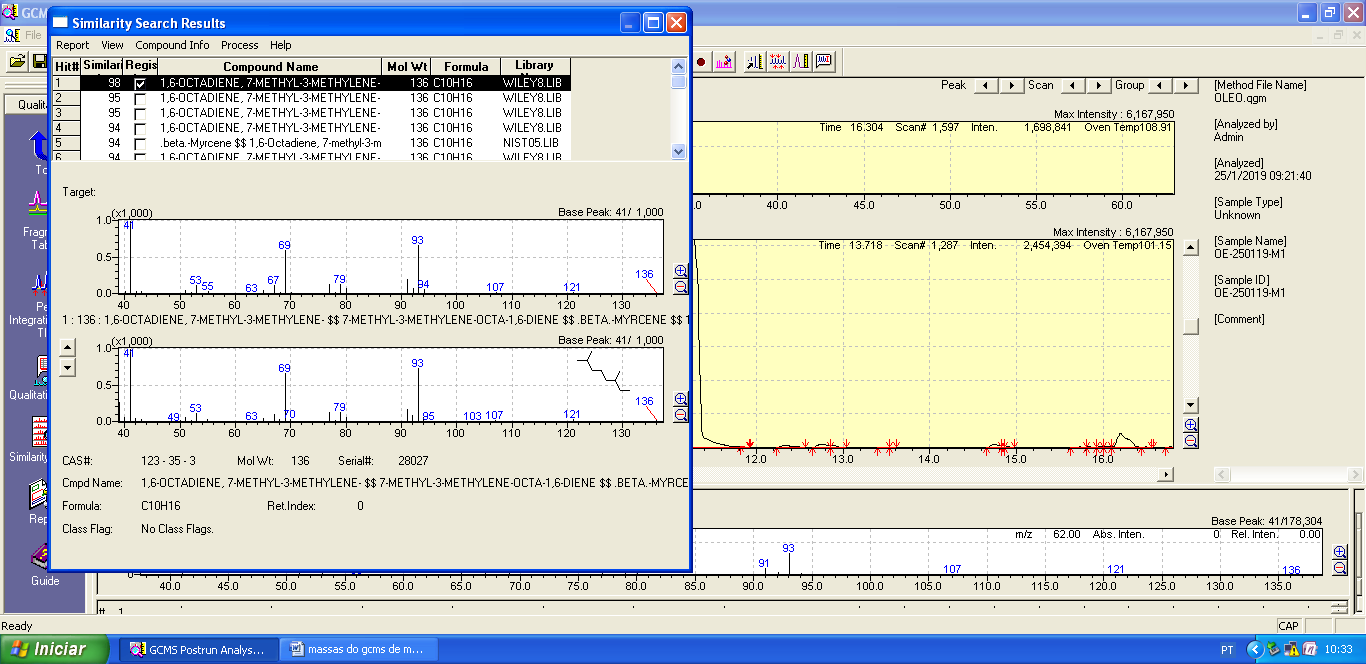


Mass spectrometer of Adams (2017).


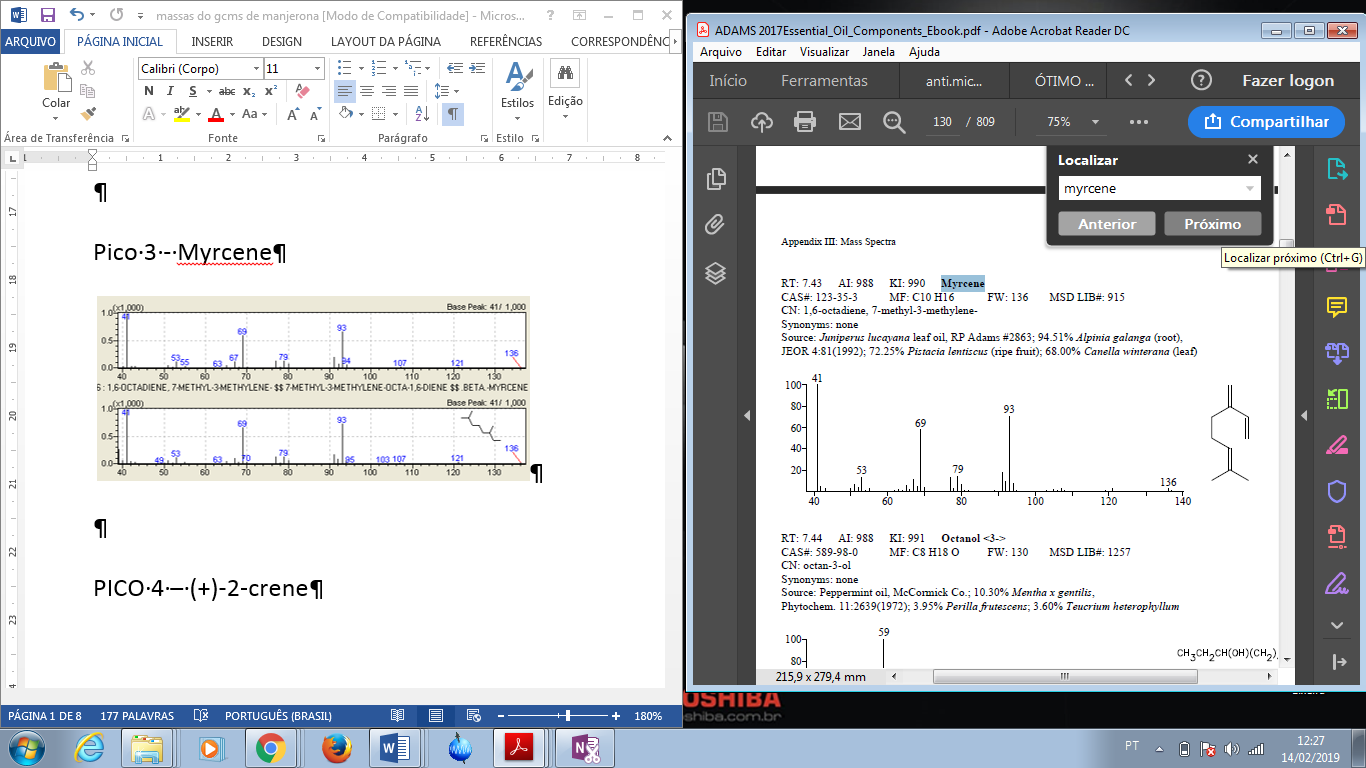


**Substance (4)**– δ-2-carene(tR = 9.500 min).


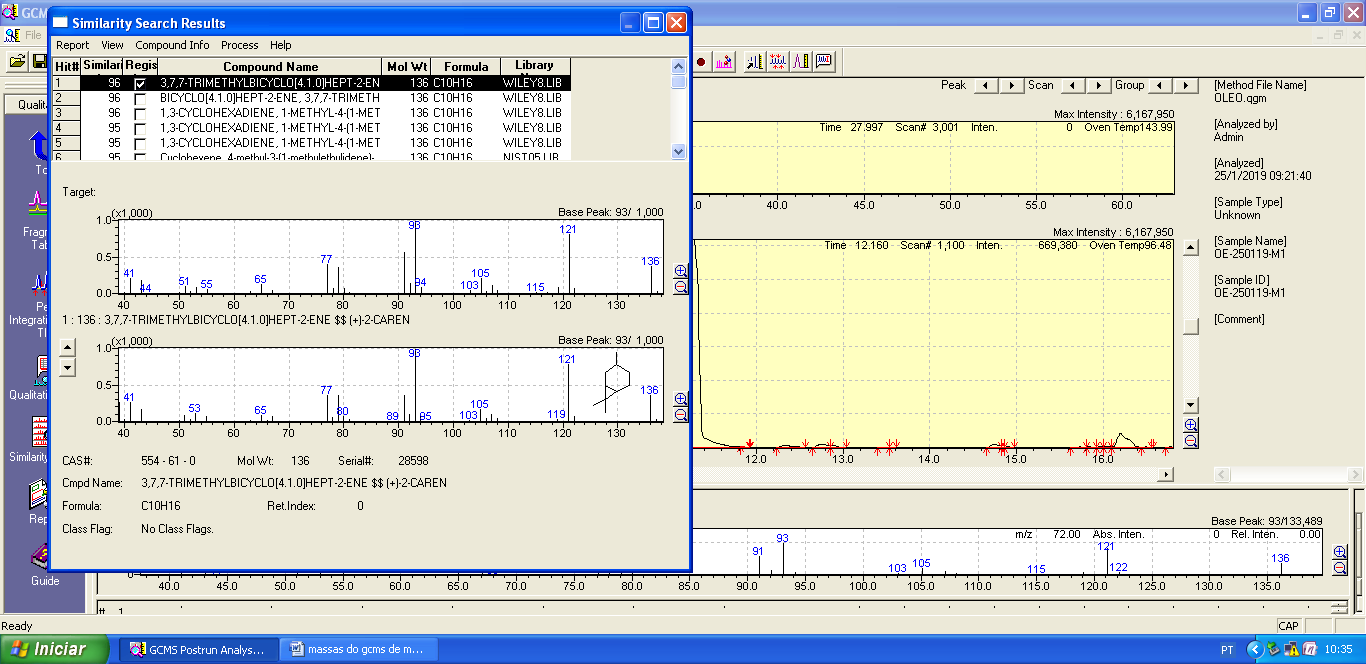


Mass spectrometer for libraries NIST05 e WILEY'S.


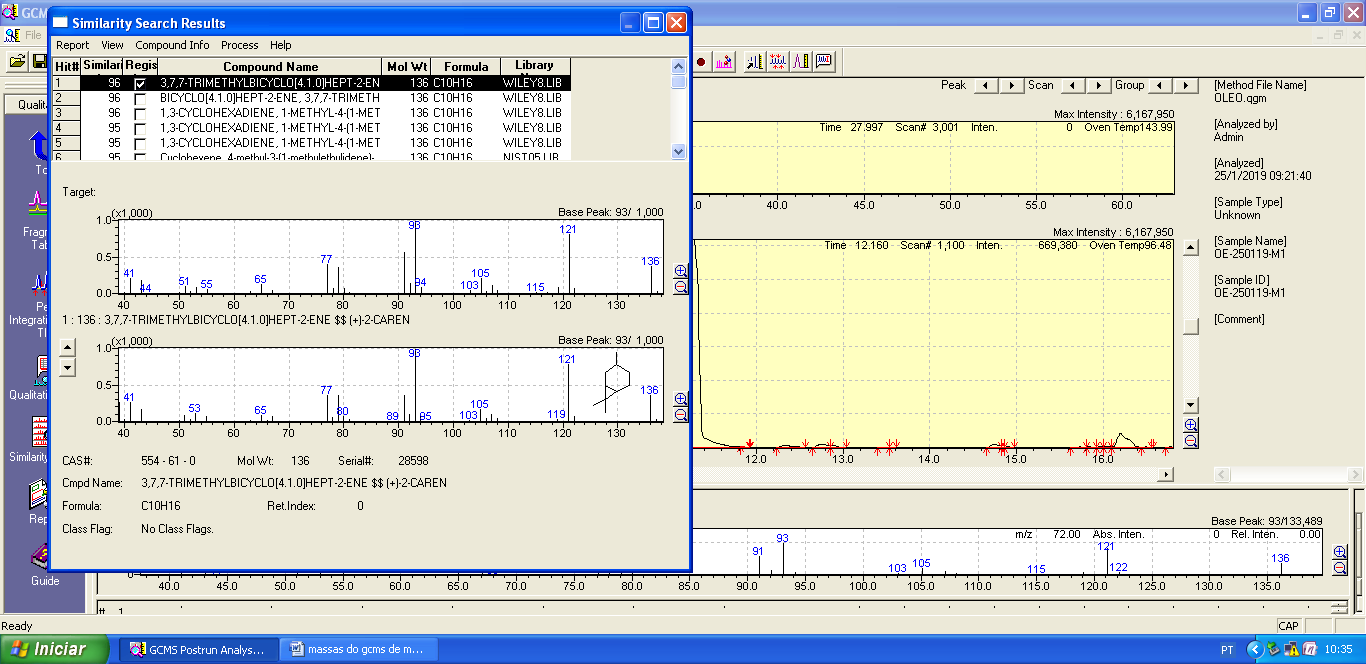


Mass spectrometer of Adams (2017).


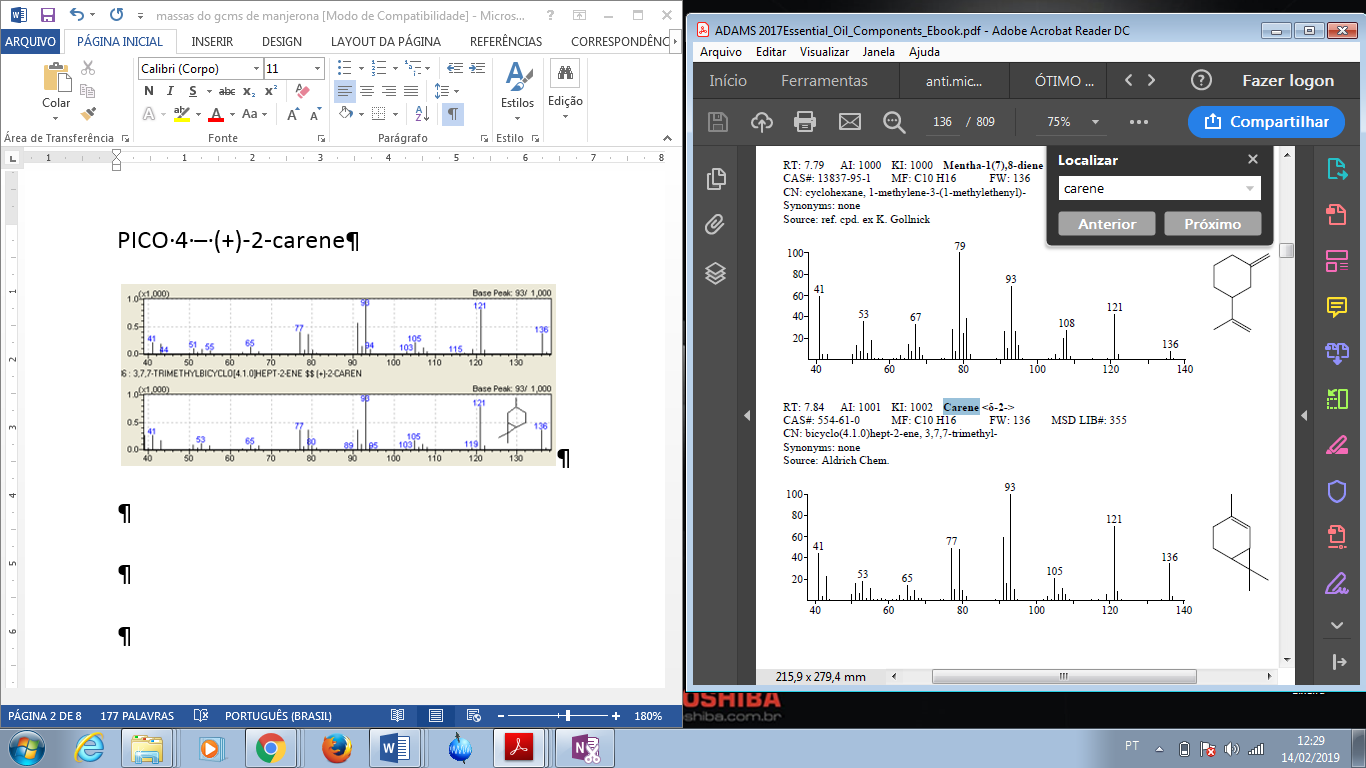


**Substance (5)** – o-cymene (tR = 9.768 min).


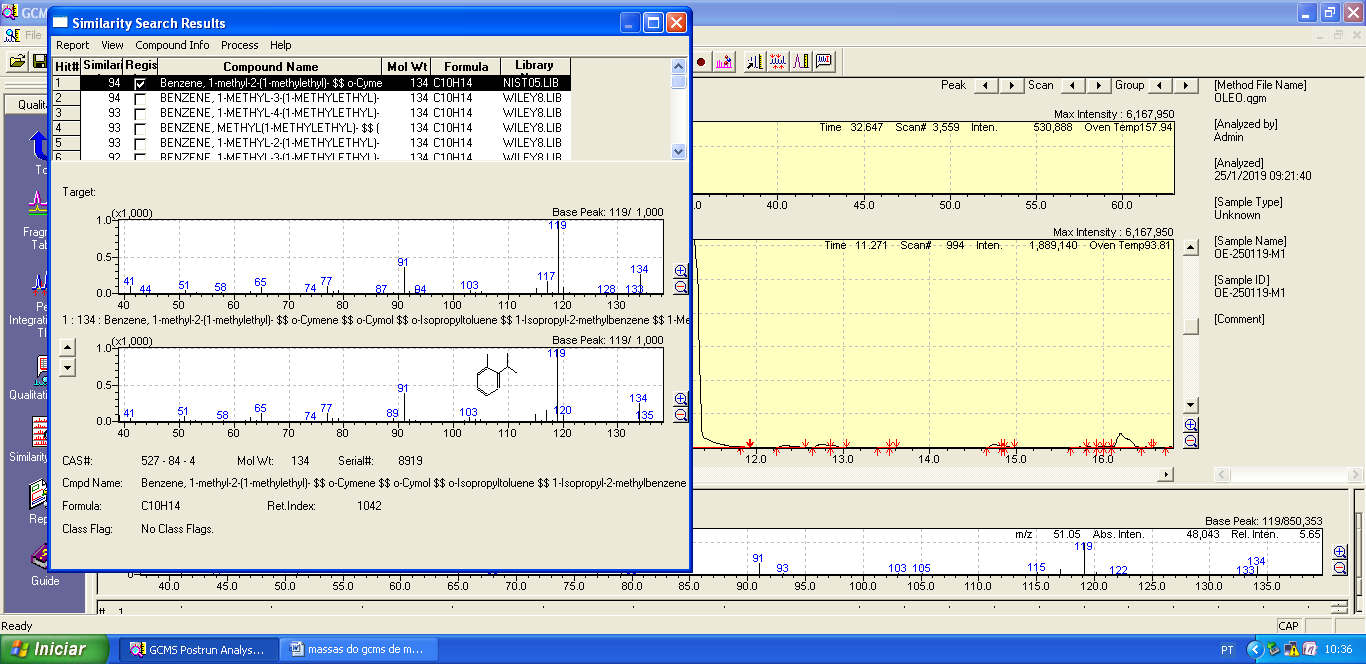


Mass spectrometer for libraries NIST05 e WILEY'S.


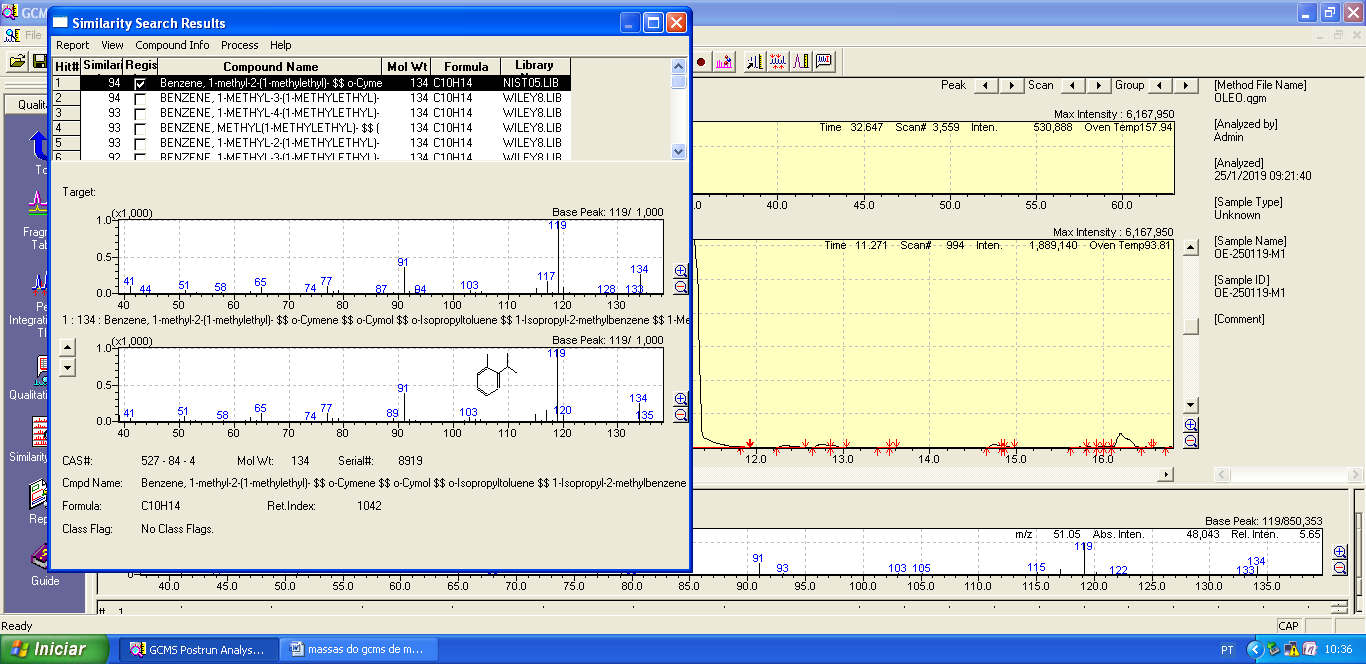


Mass spectrometer of Adams (2017).


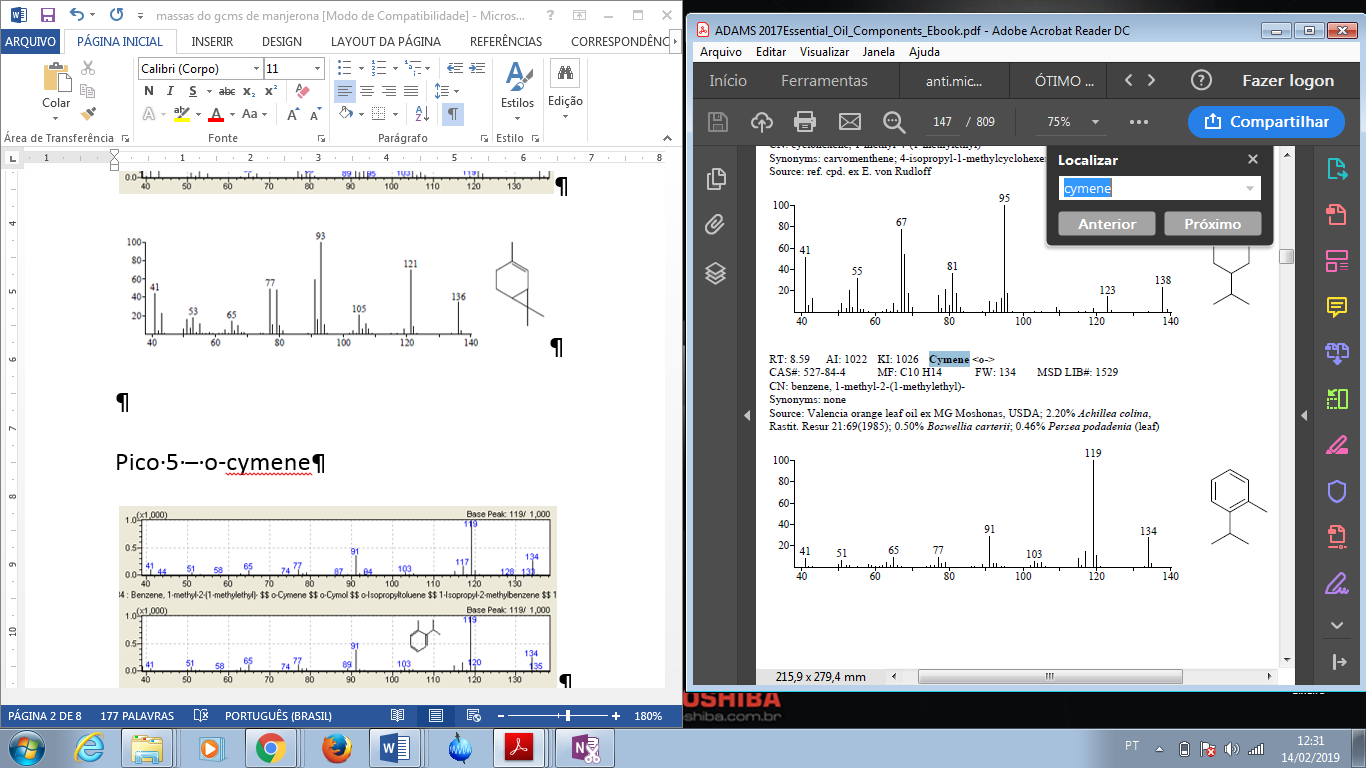


**Substance (6)**–p-cymene (tR = 9.991 min).


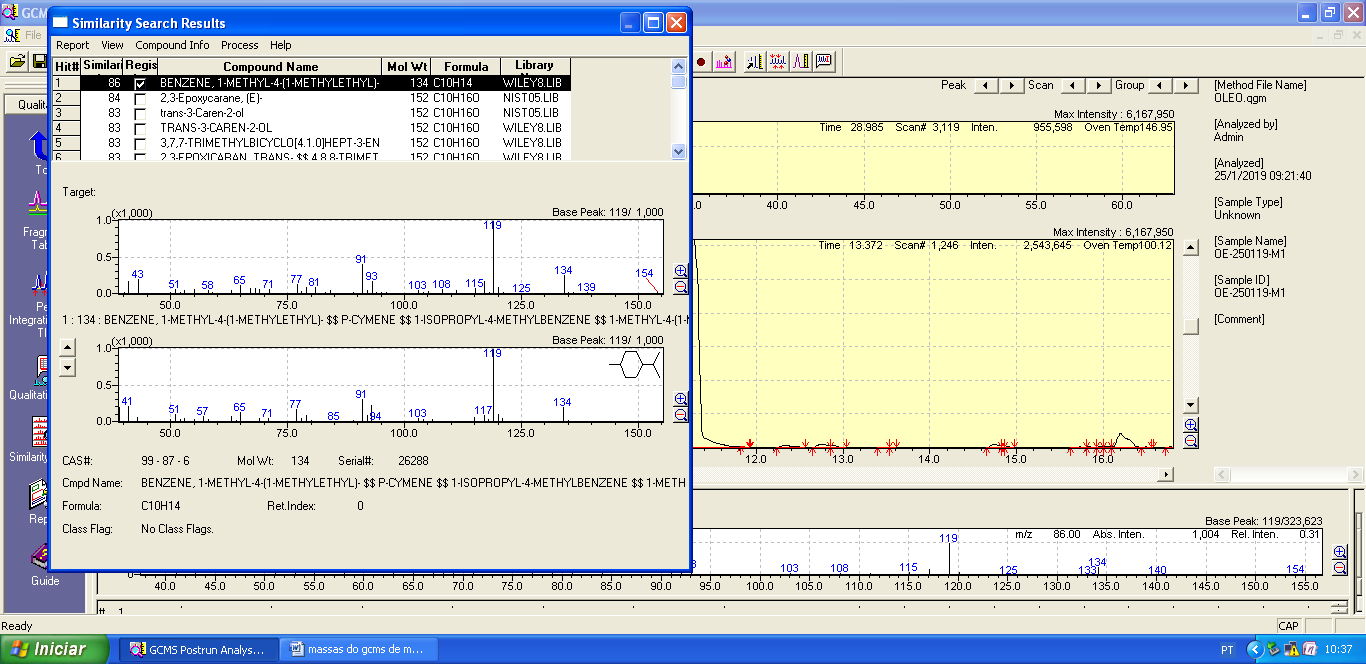


Mass spectrometer for libraries NIST05 e WILEY'S.


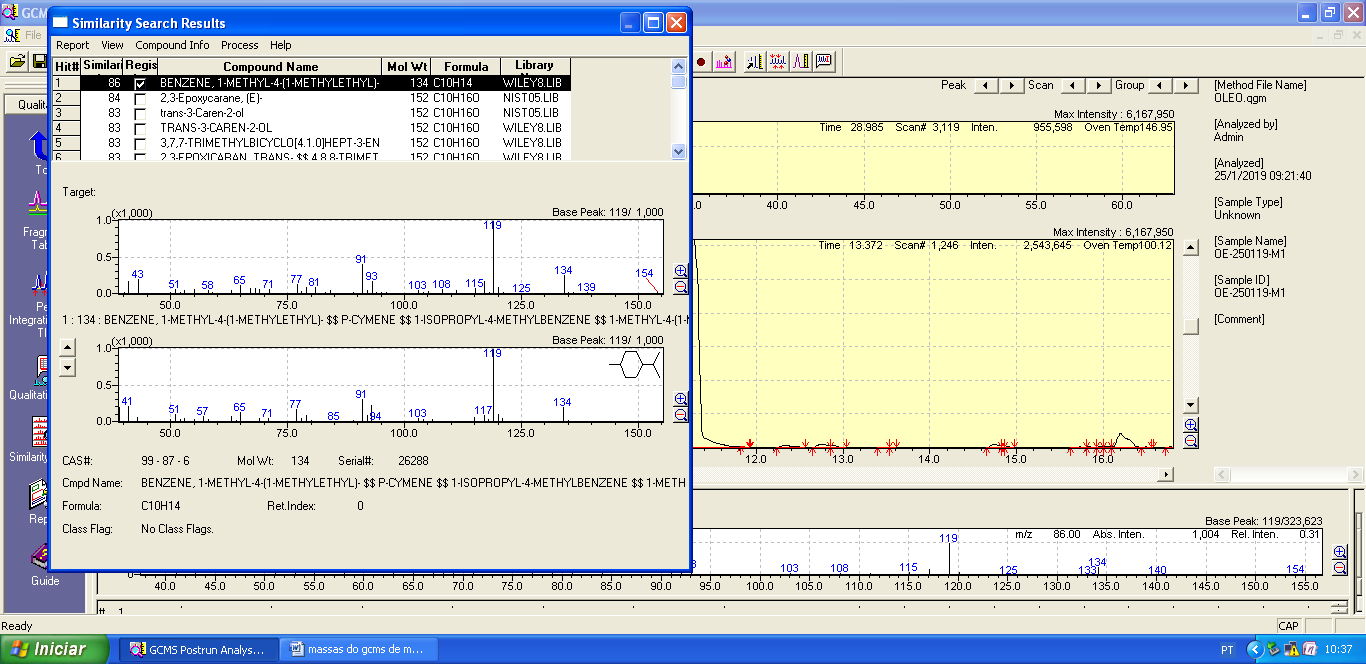


Mass spectrometer of Adams (2017).


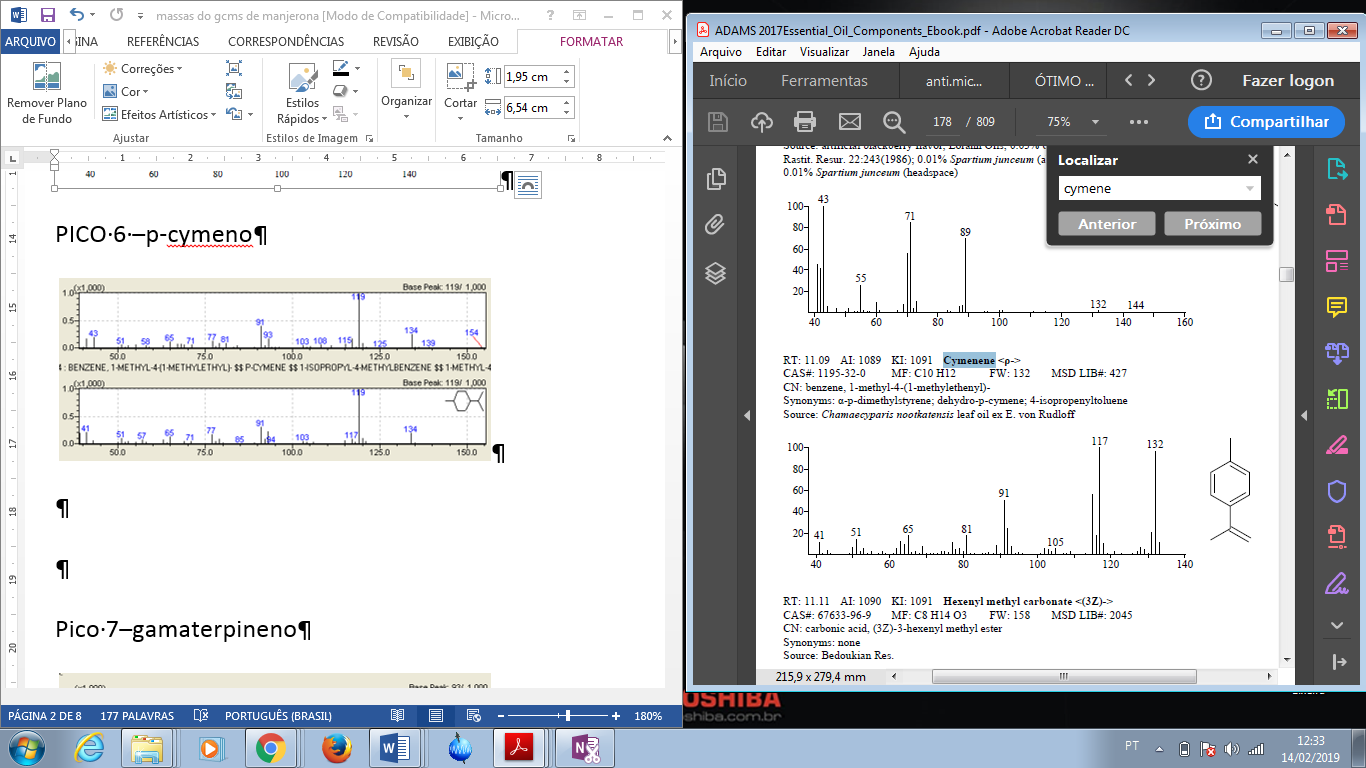


**Substance (7)**– γ- terpinene(tR = 11.195 min).


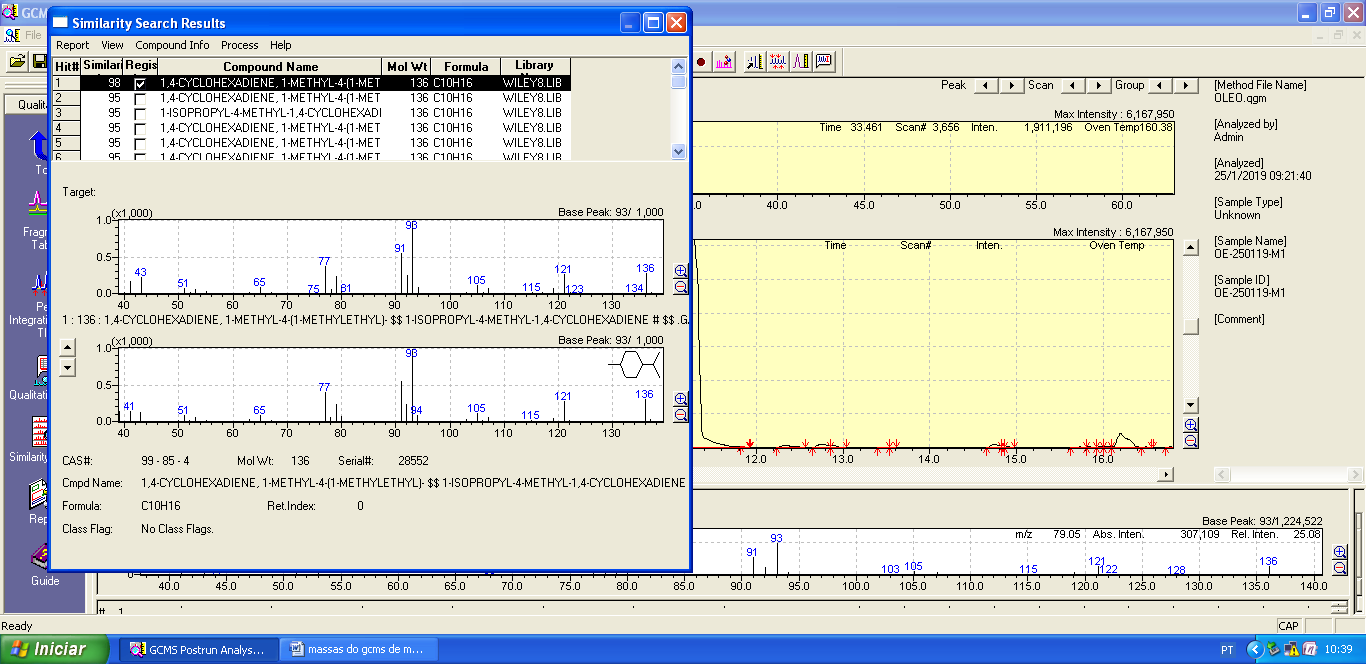


Mass spectrometer for libraries NIST05 e WILEY'S.


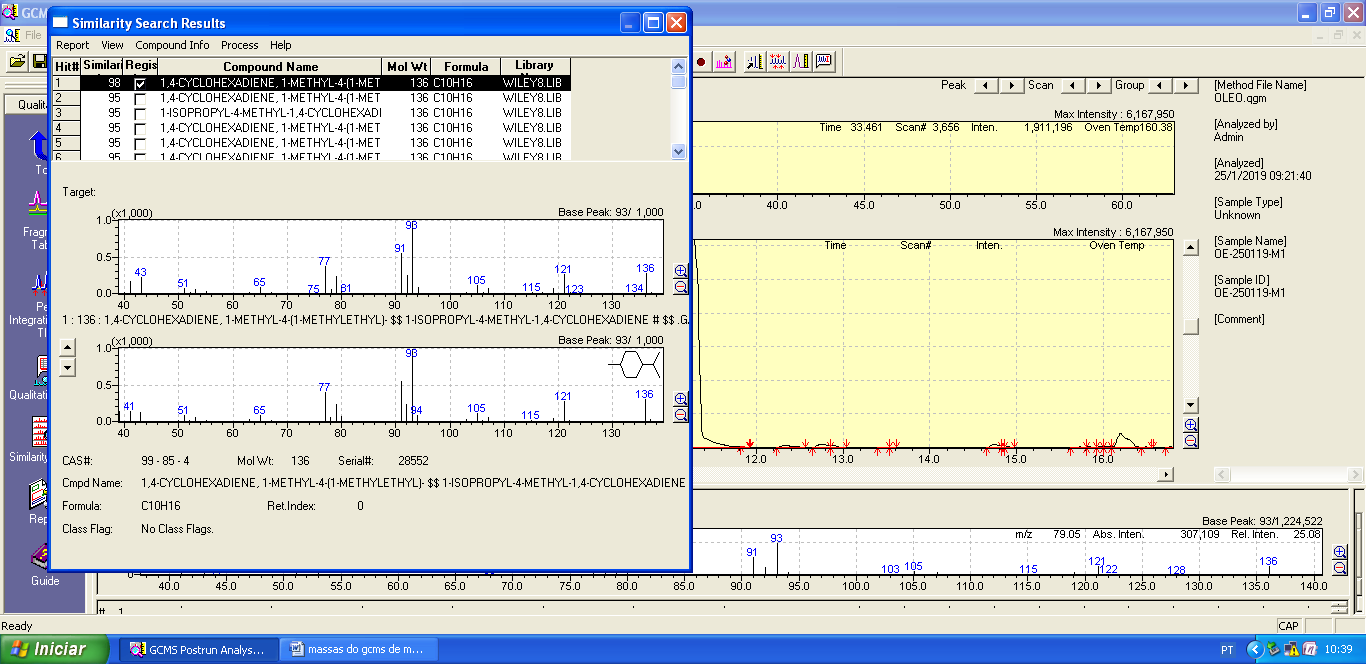


Mass spectrometer of Adams (2017).


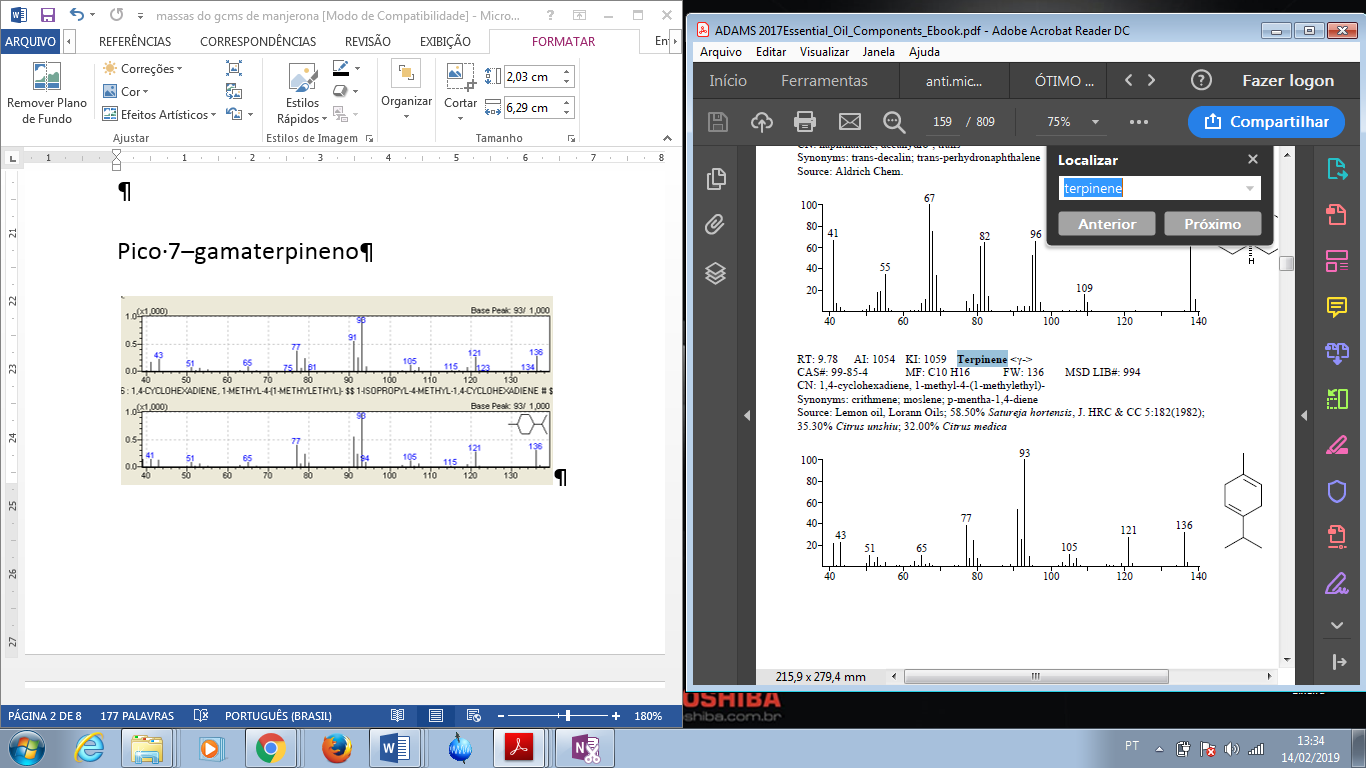


**Substance (8)**- α- terpinene(tR = 12.339 min).


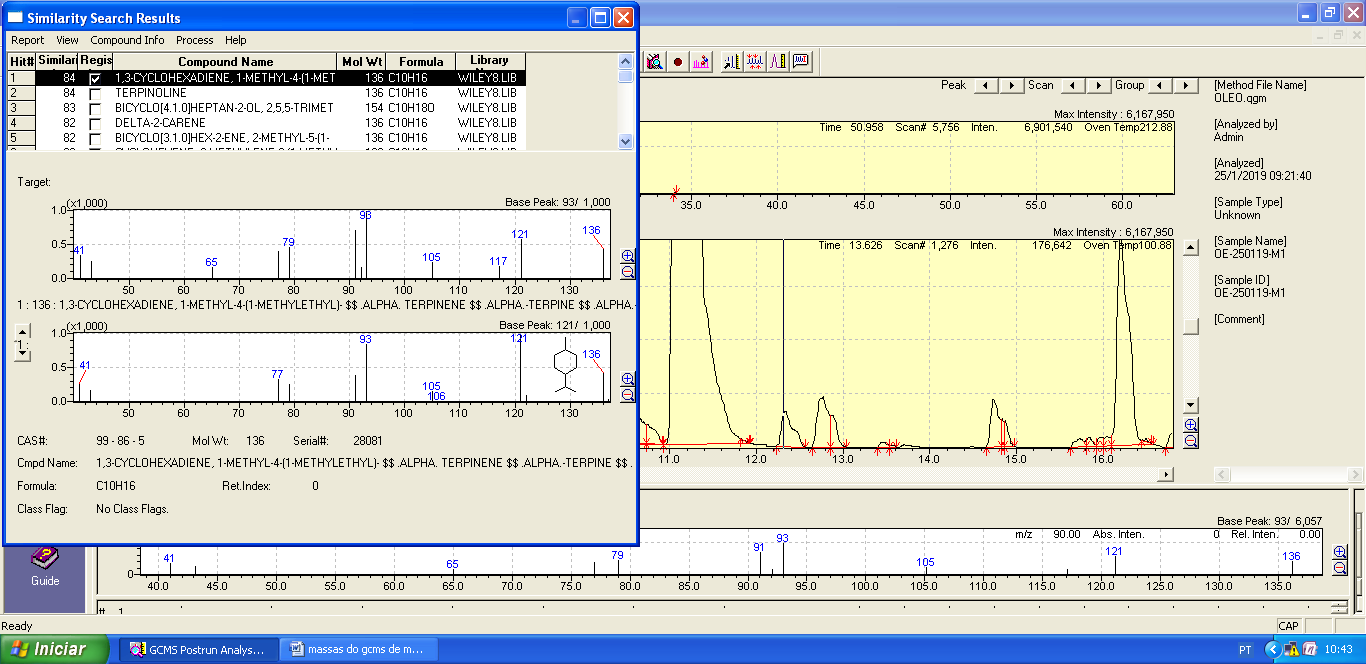


Mass spectrometer for libraries NIST05 e WILEY'S.


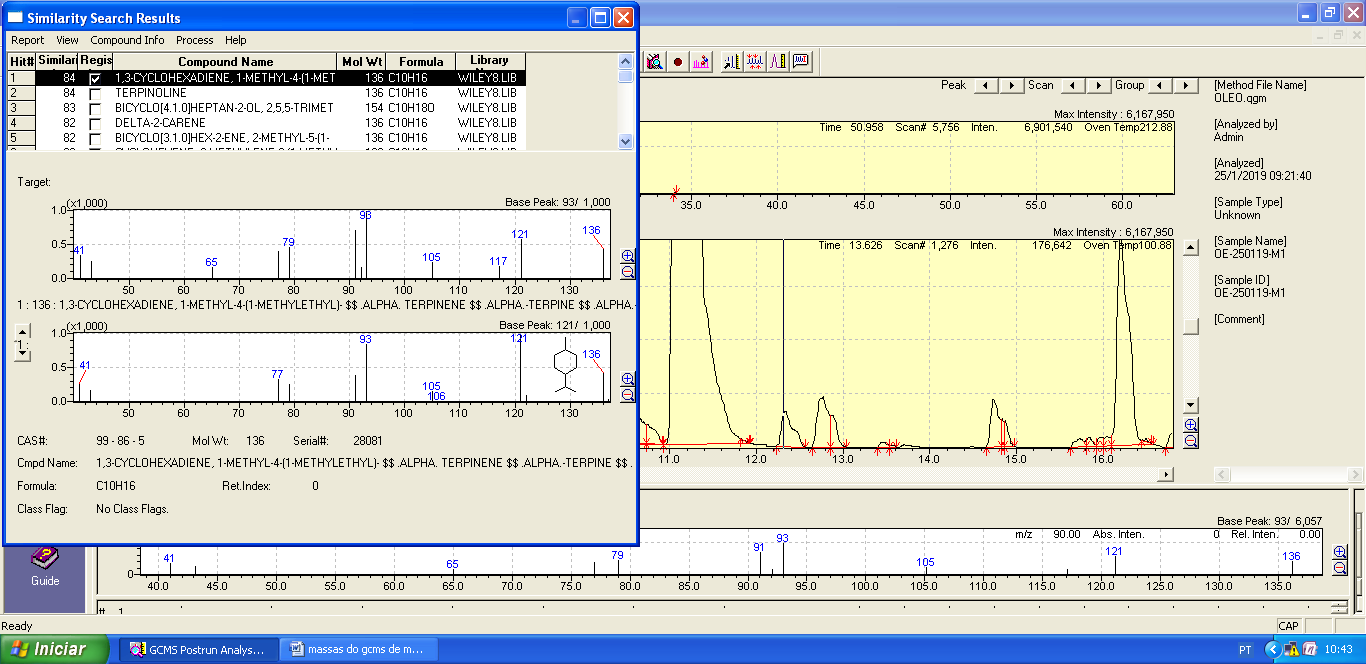


Mass spectrometer of o Adams (2017).


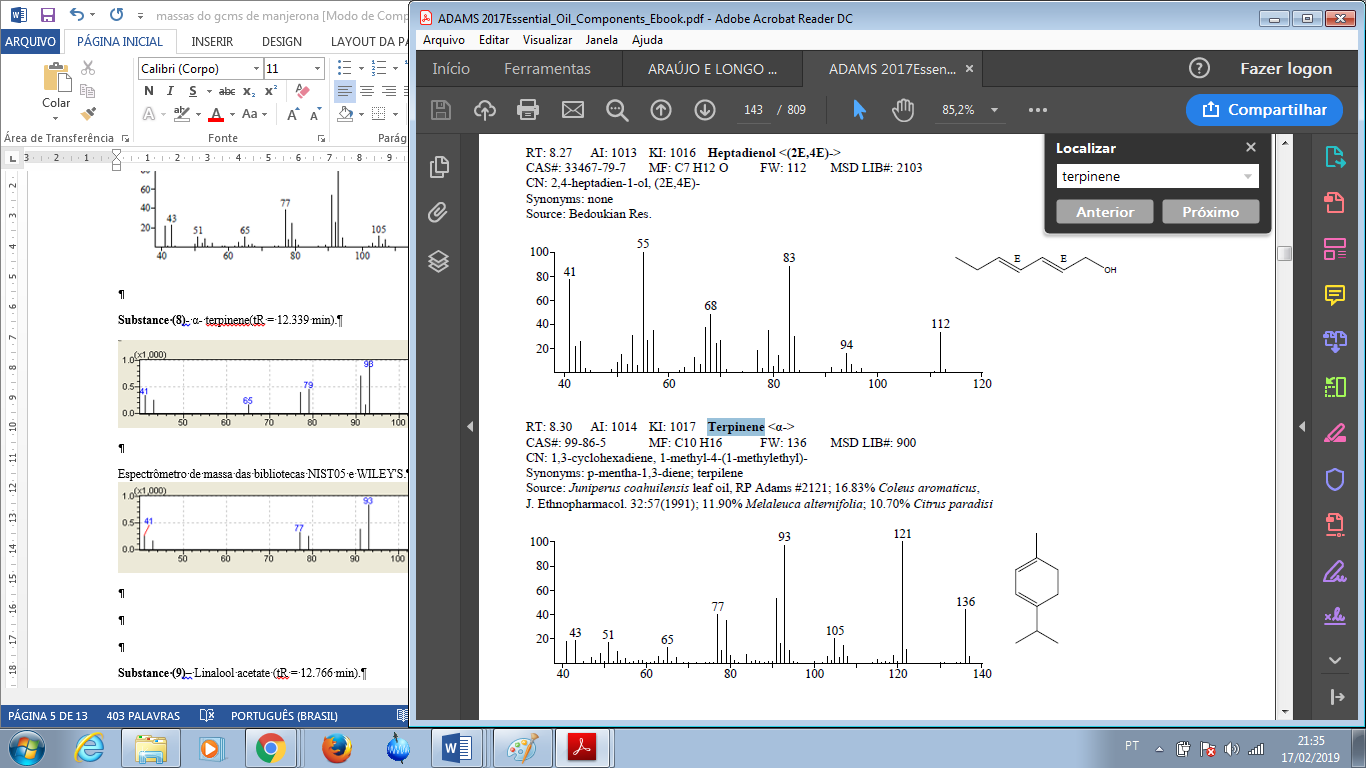


**Substance (9)** – Linalool acetate (tR = 12.766 min).


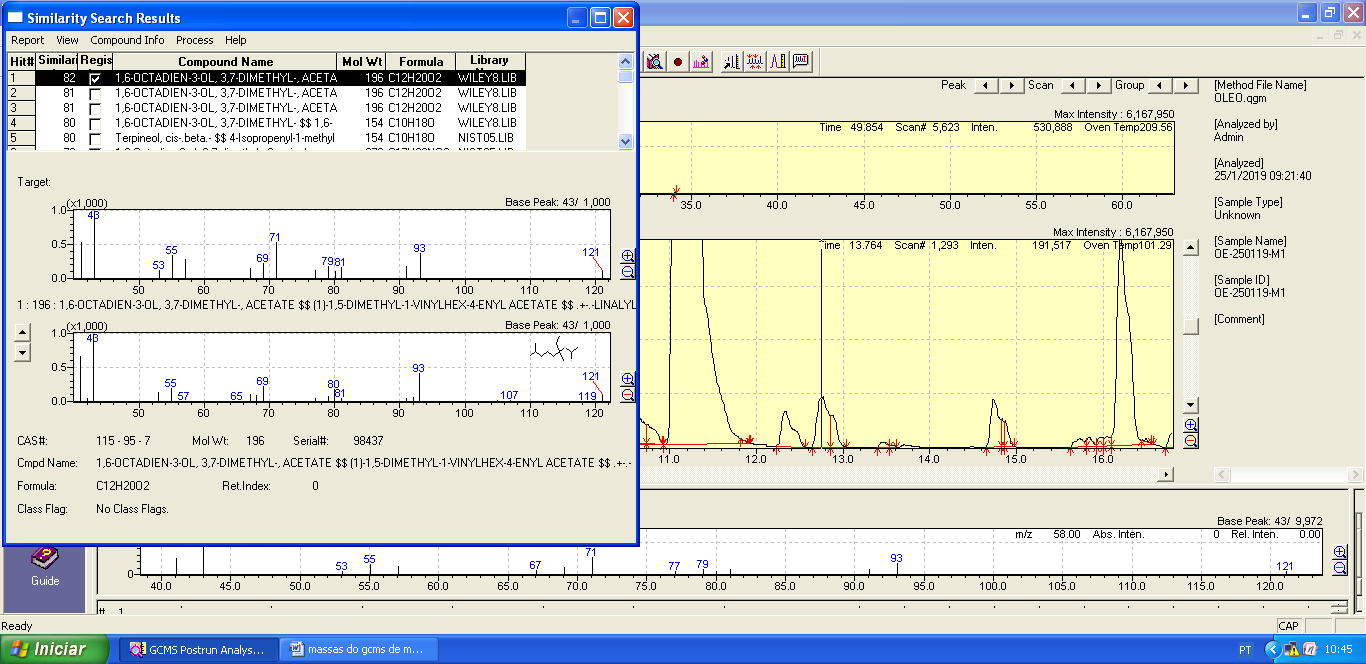


Espectrômetro de massa das bibliotecas NIST05 e WILEY'S.


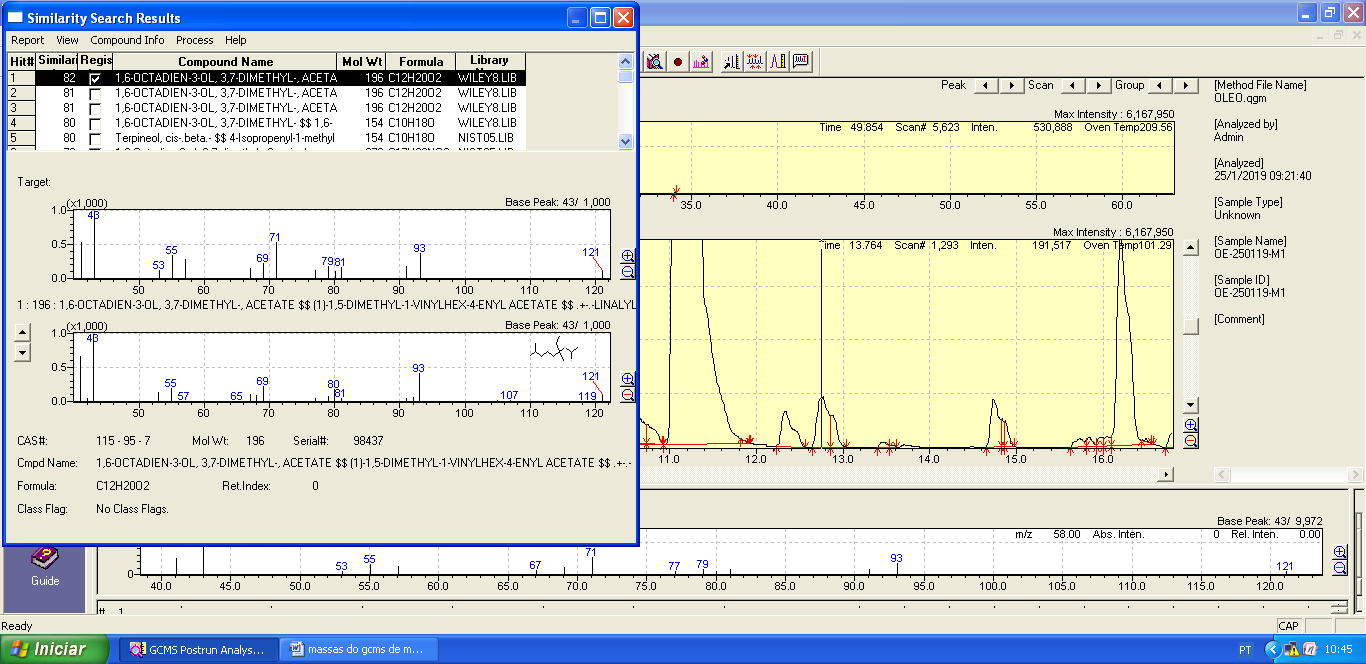


Mass spectrometer of Adams (2017).


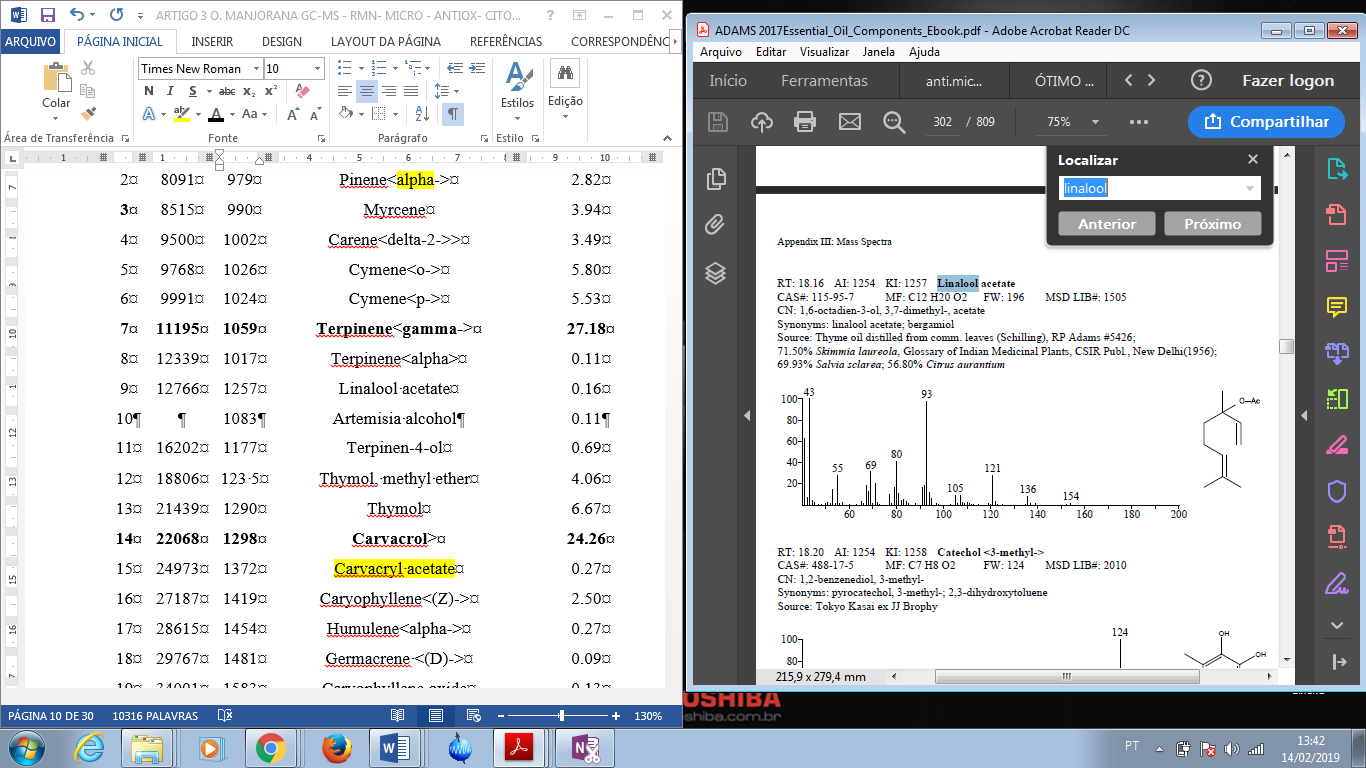


**Substance (10)** – ArtemisiaAlcohol(tR = 14.732 min).


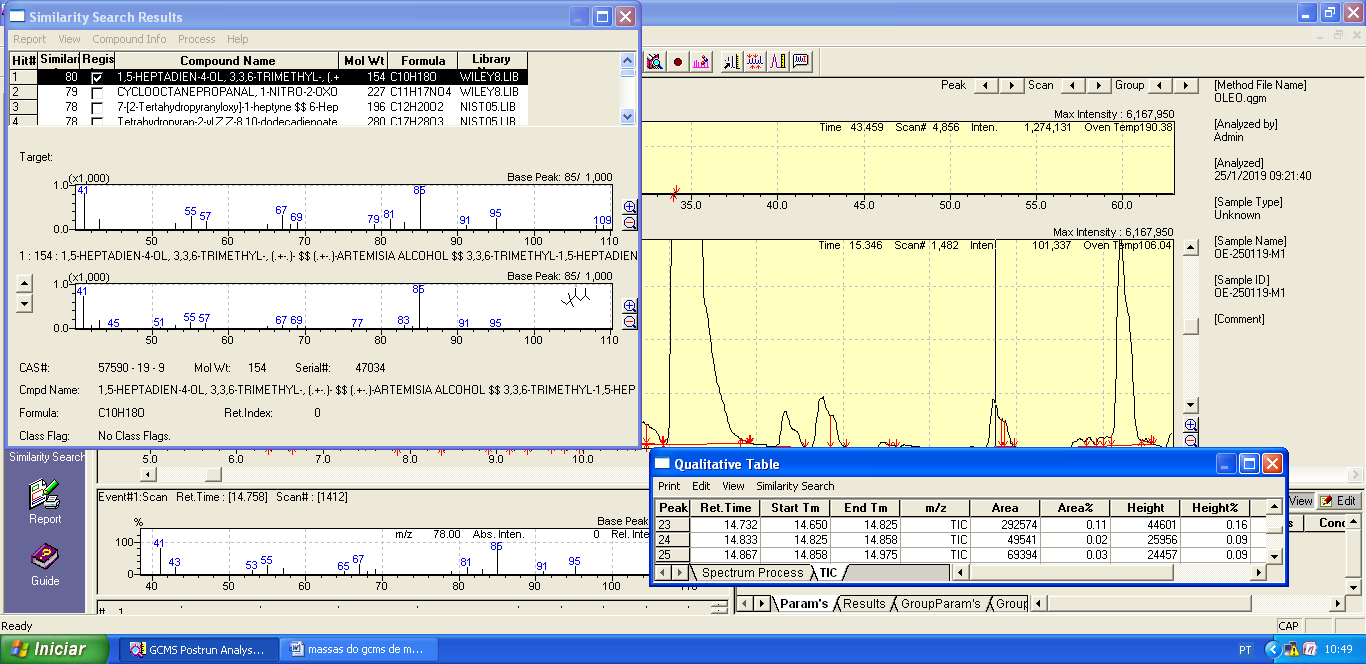


Mass spectrometer for libraries NIST05 e WILEY'S.


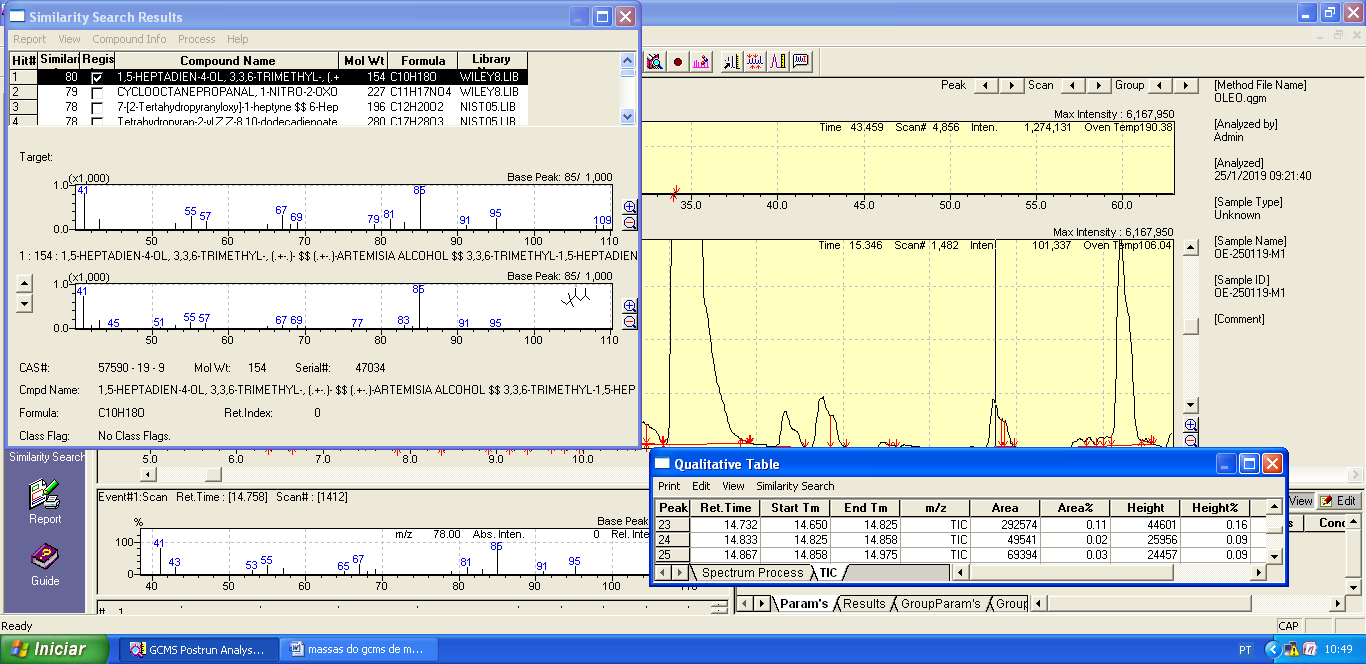


Mass spectrometer of Adams (2017).


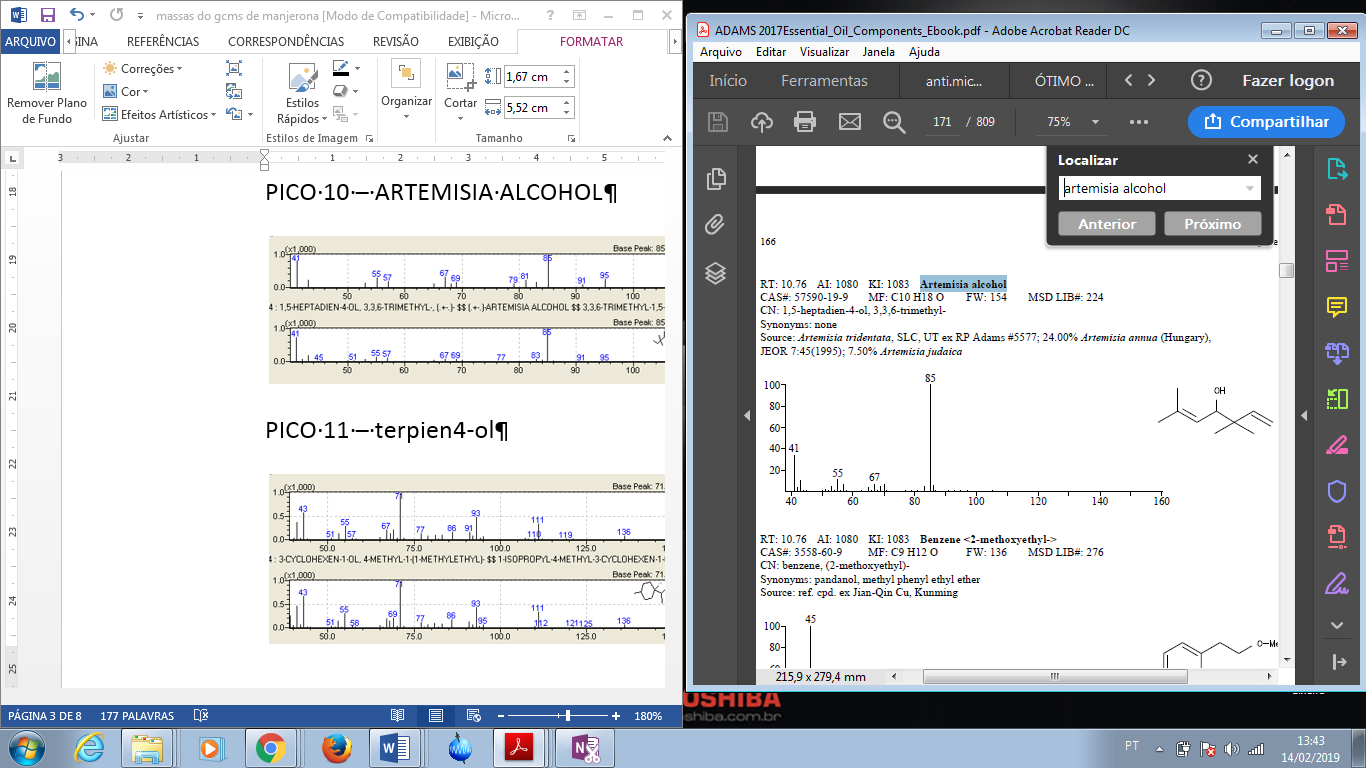


**Substance (11)** – terpinen-4-ol(tR = 16.202 min).


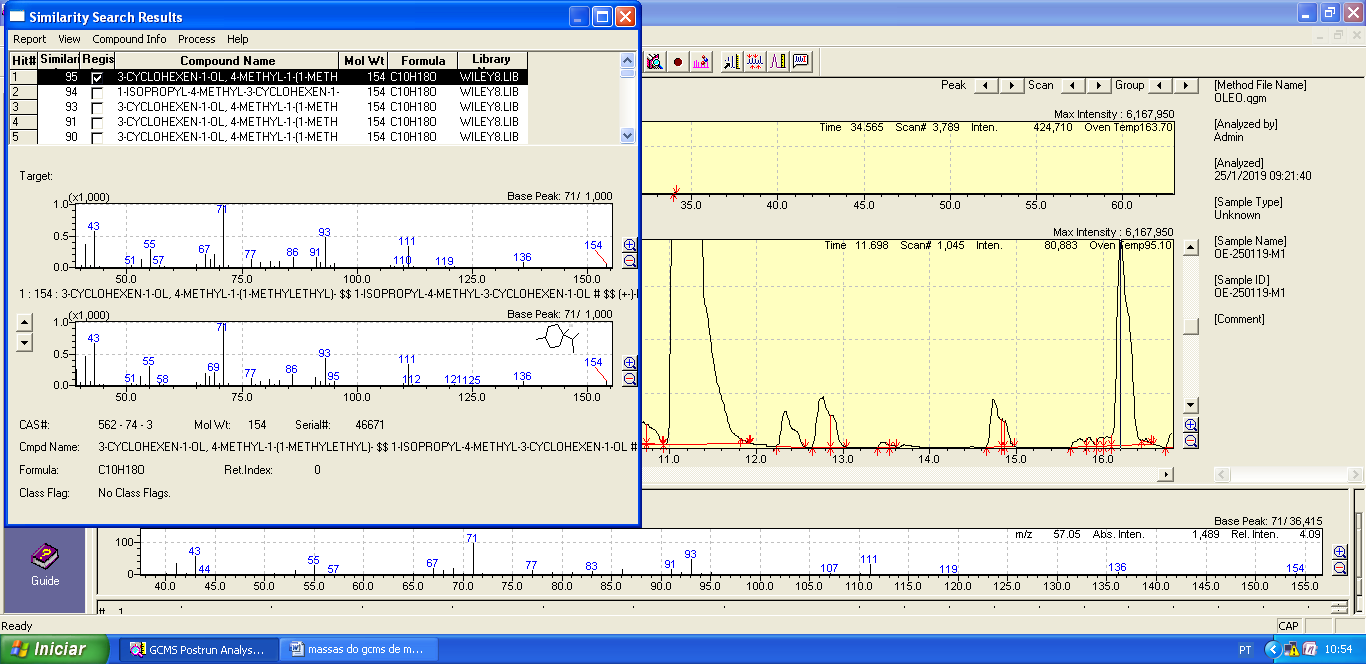


Mass spectrometer for libraries NIST05 e WILEY'S.


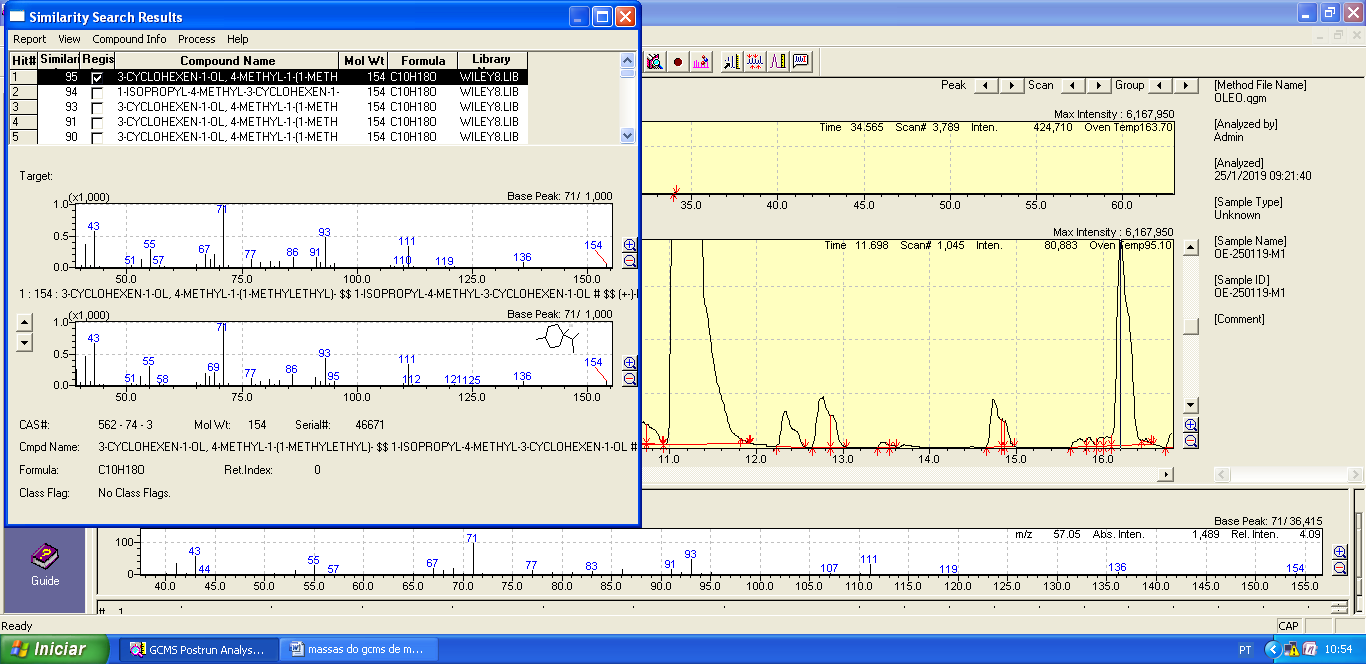


Mass spectrometer of Adams (2017).


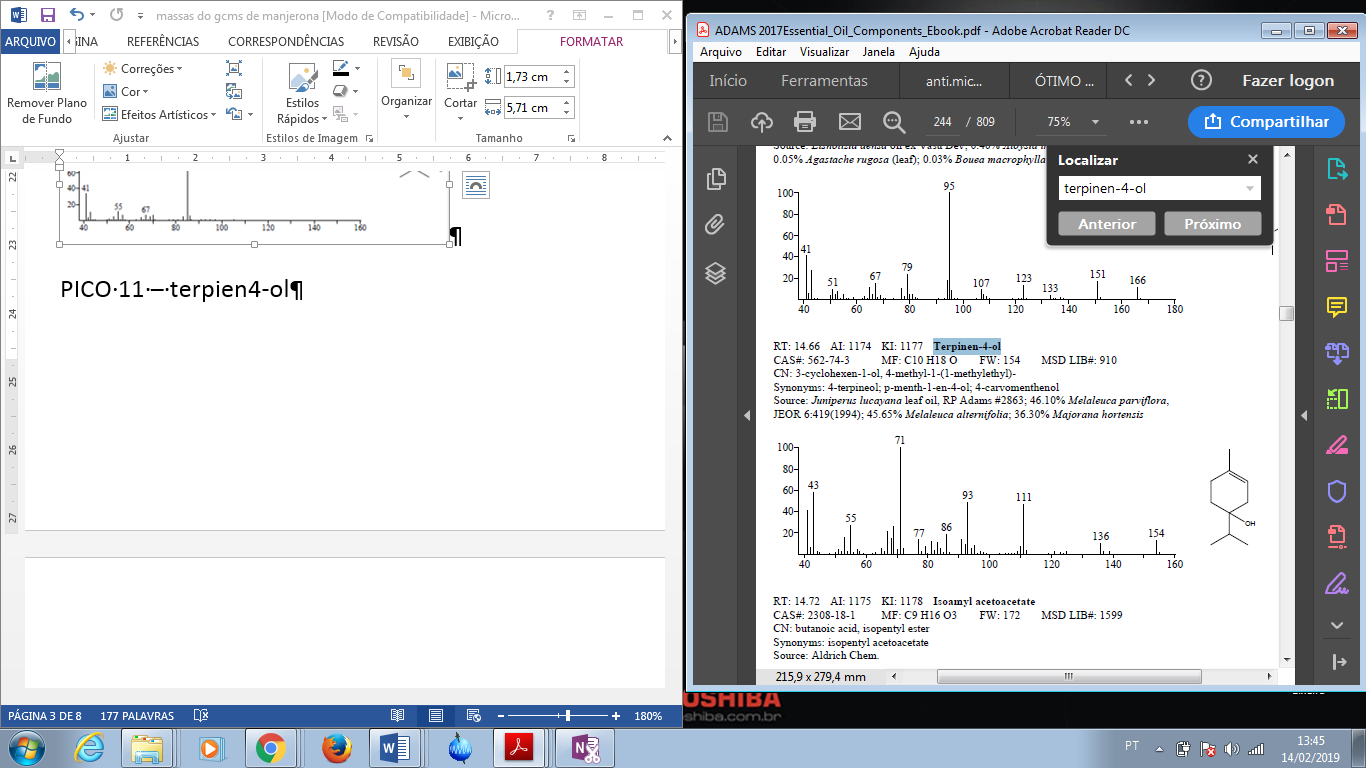


**Substance (12)** – Thymol, Methyl ether(tR = 18.806 min).


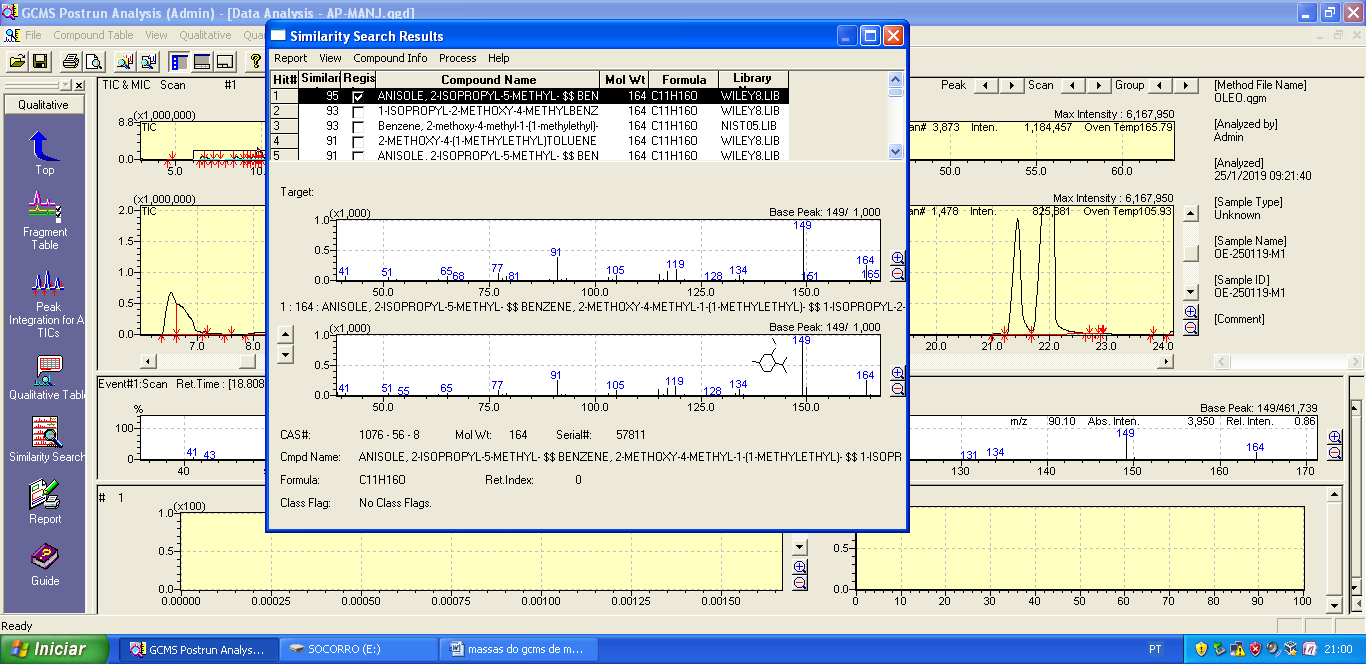


Mass spectrometer for libraries NIST05 e WILEY'S.


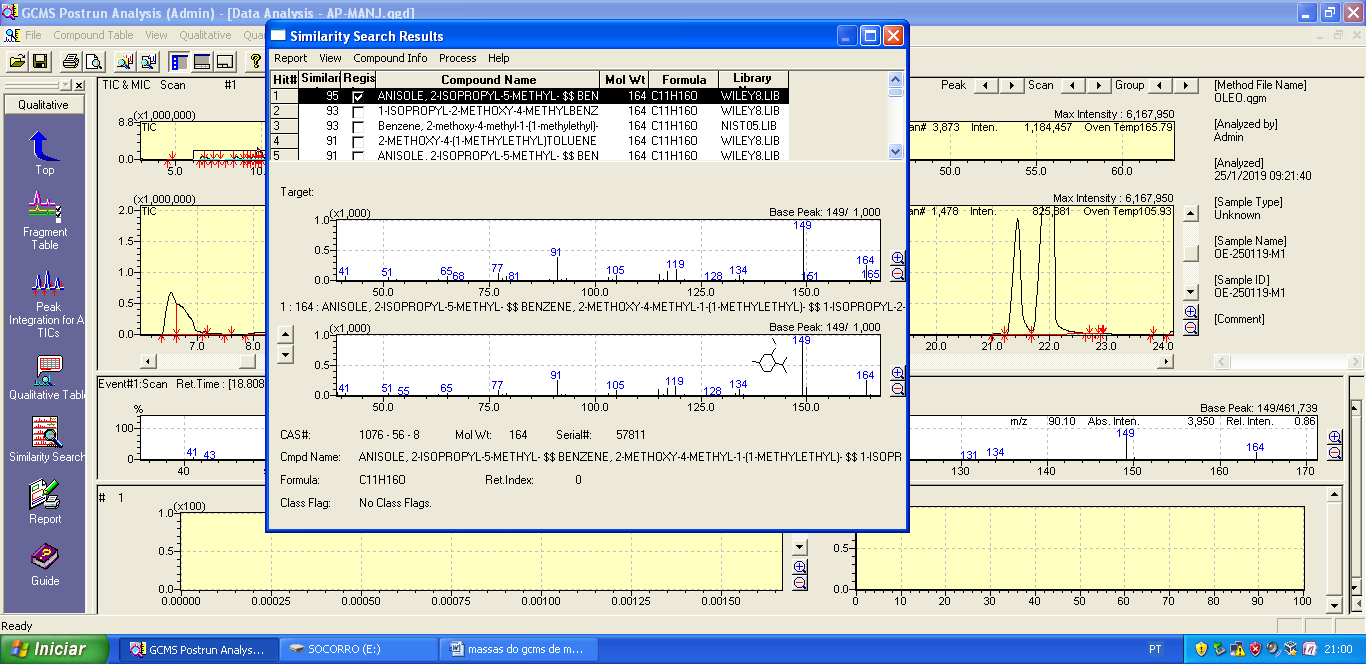


Mass spectrometer of Adams (2017).


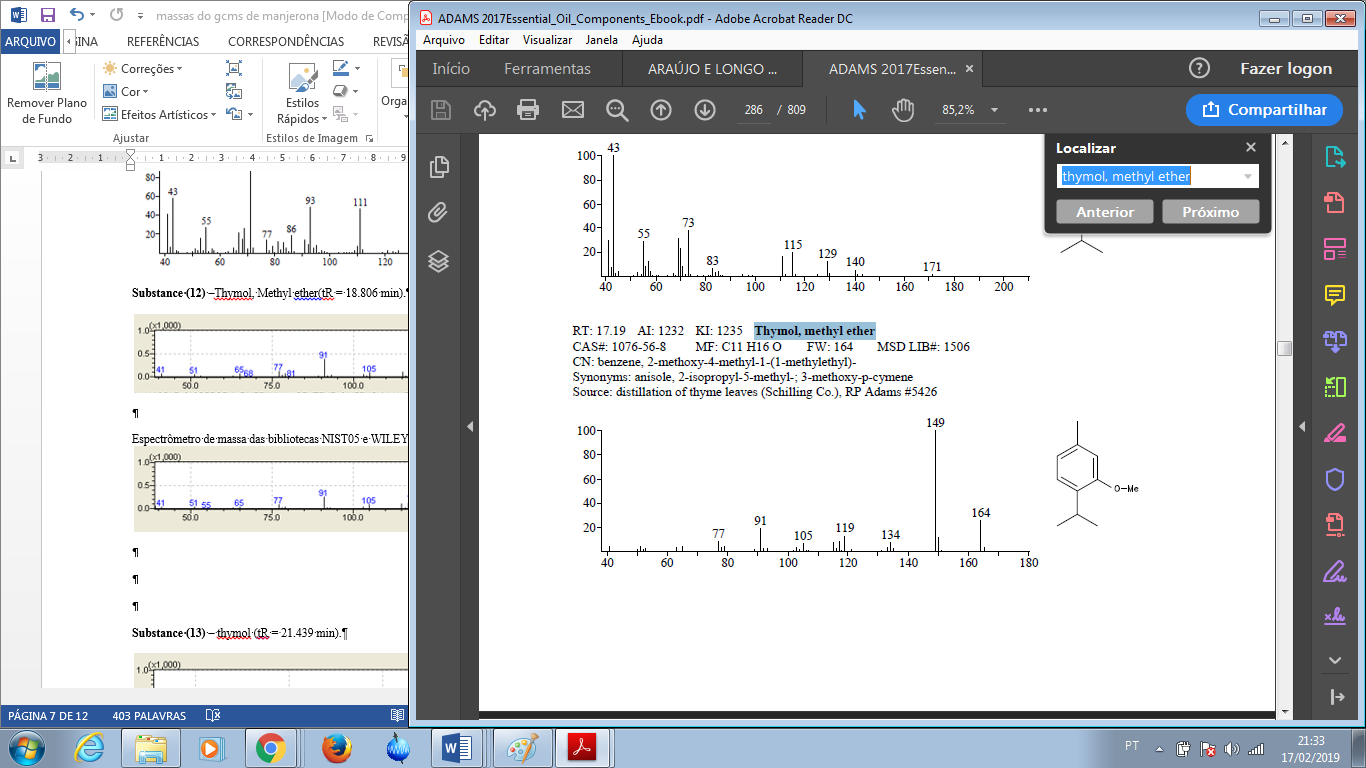


**Substance (13)** – thymol (tR = 21.439 min).


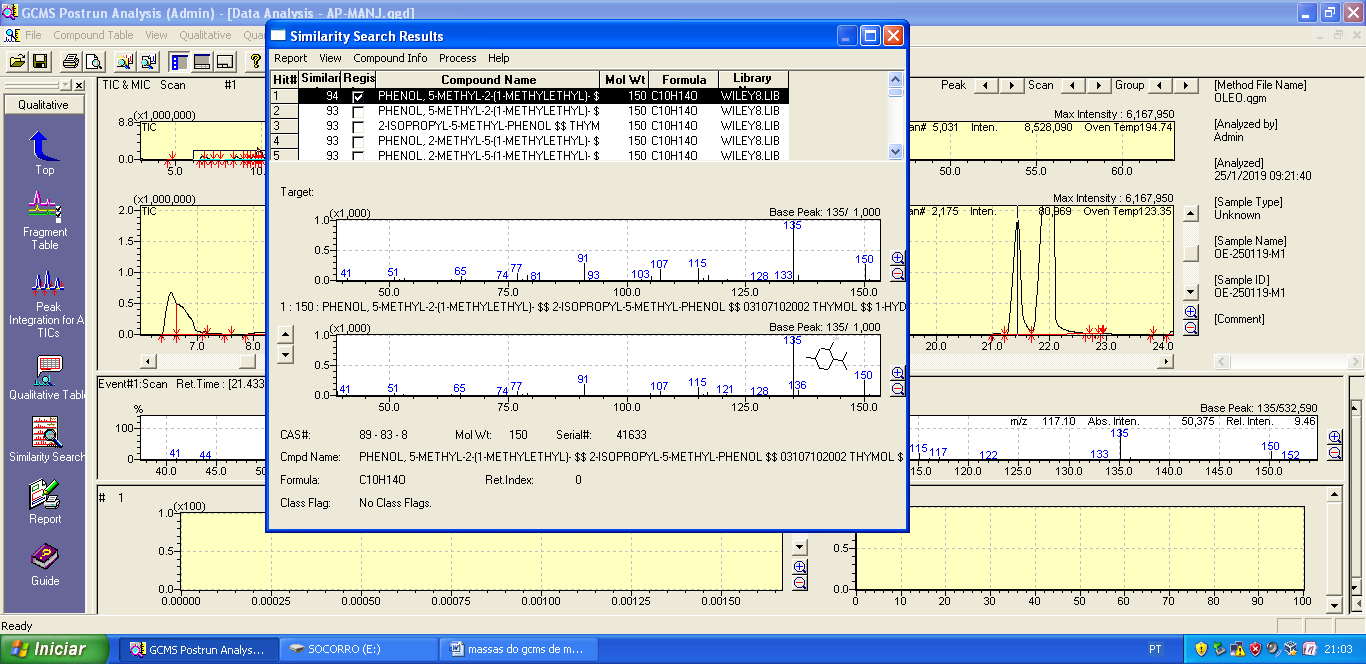


Mass spectrometer for libraries NIST05 e WILEY'S.


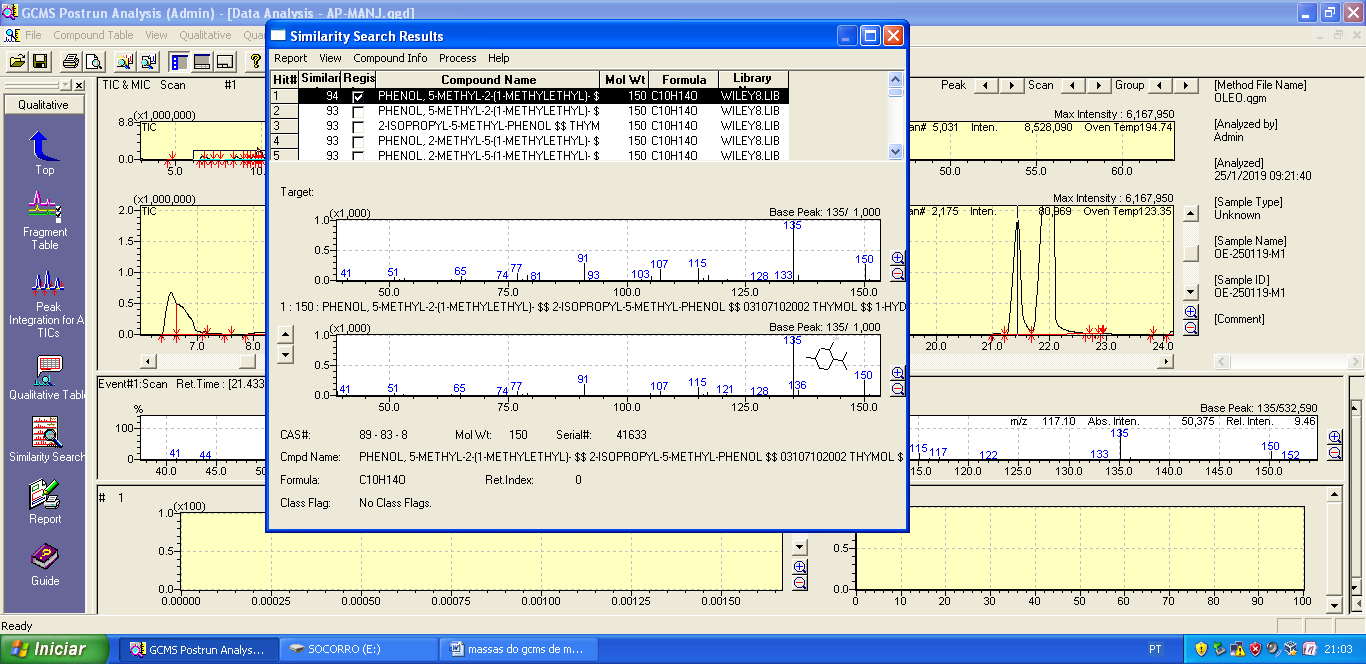


Mass spectrometer of Adams (2017).


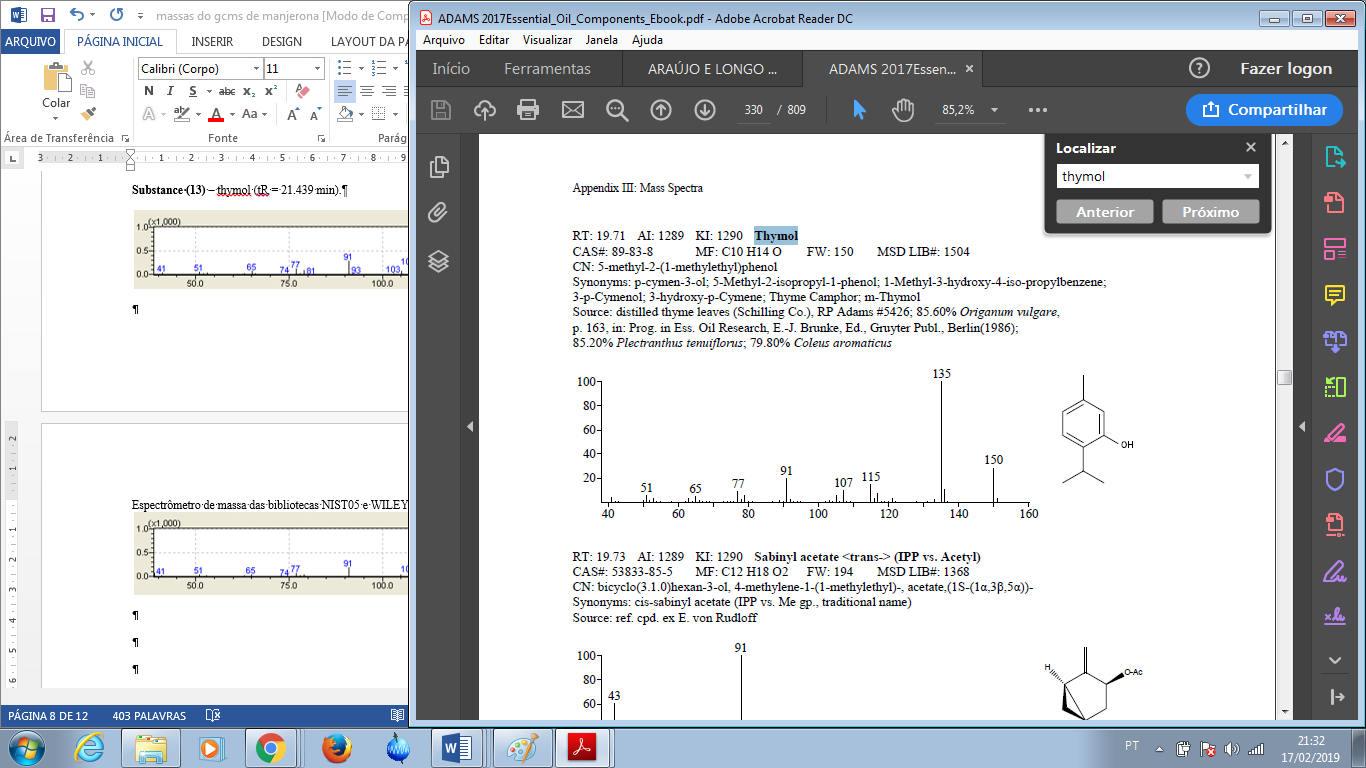


**Substance (14)** – Carvacrol (tR = 22.068 min).


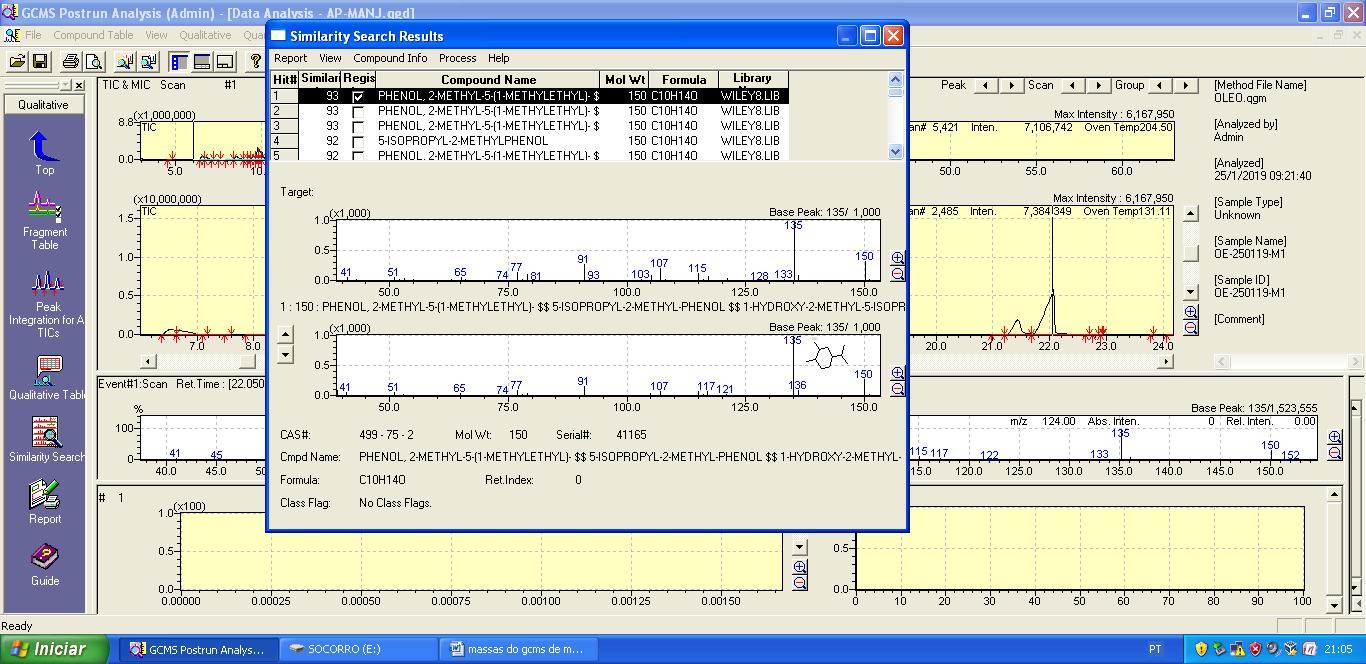


Mass spectrometer for libraries NIST05 e WILEY'S.


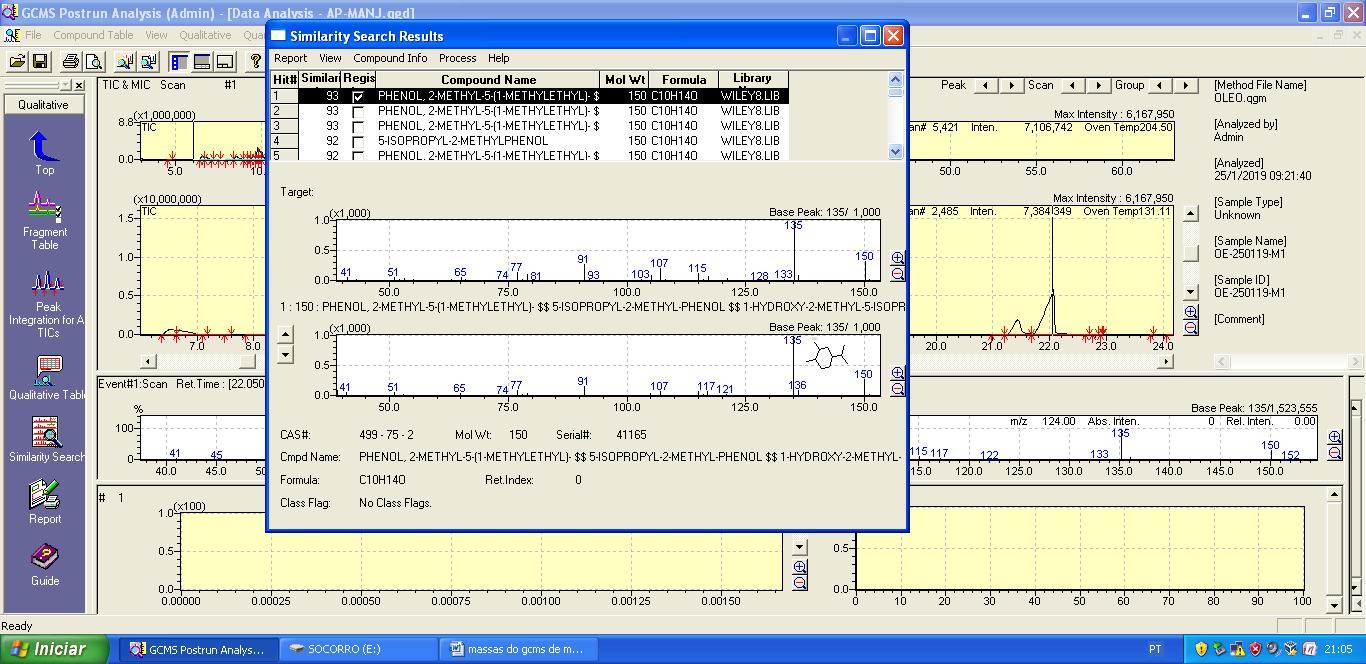


Mass spectrometer of Adams (2017).


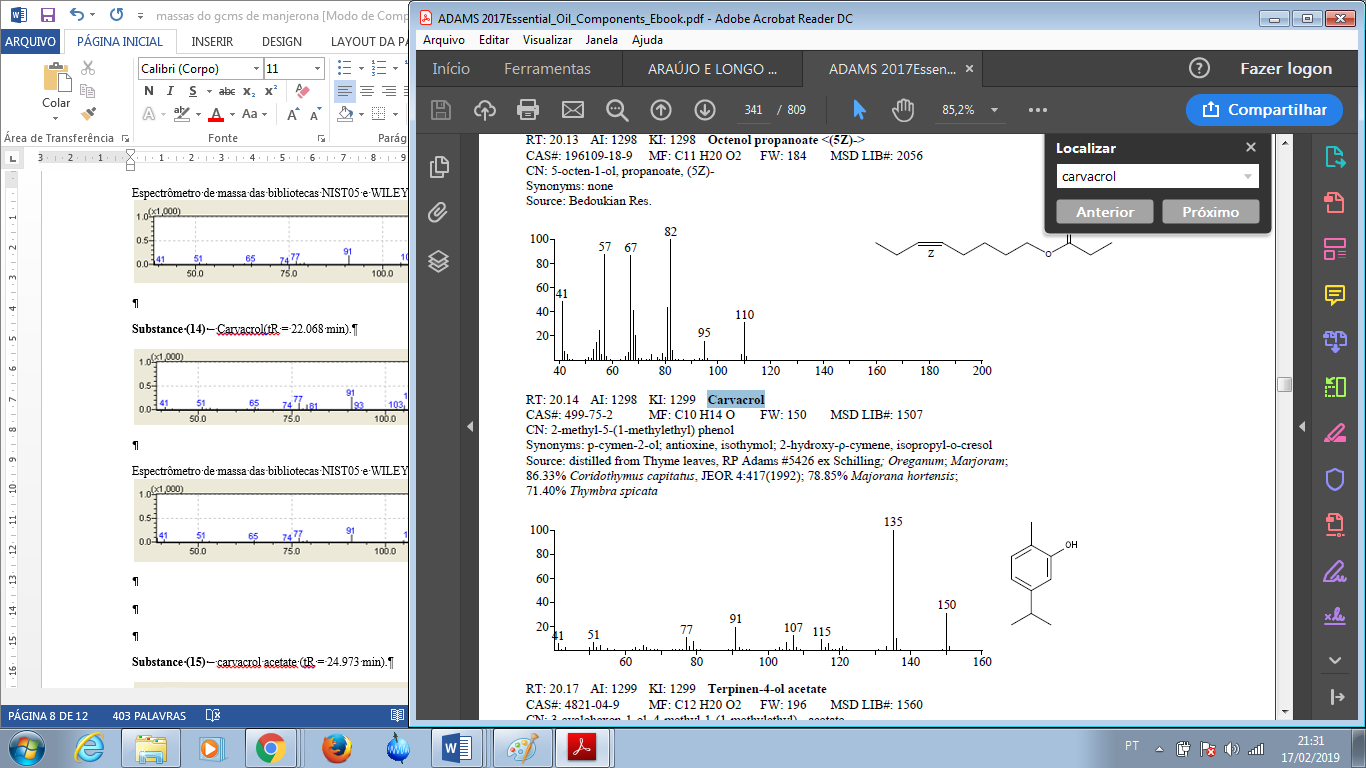


**Substance (15)** – carvacrol acetate (tR = 24.973 min).


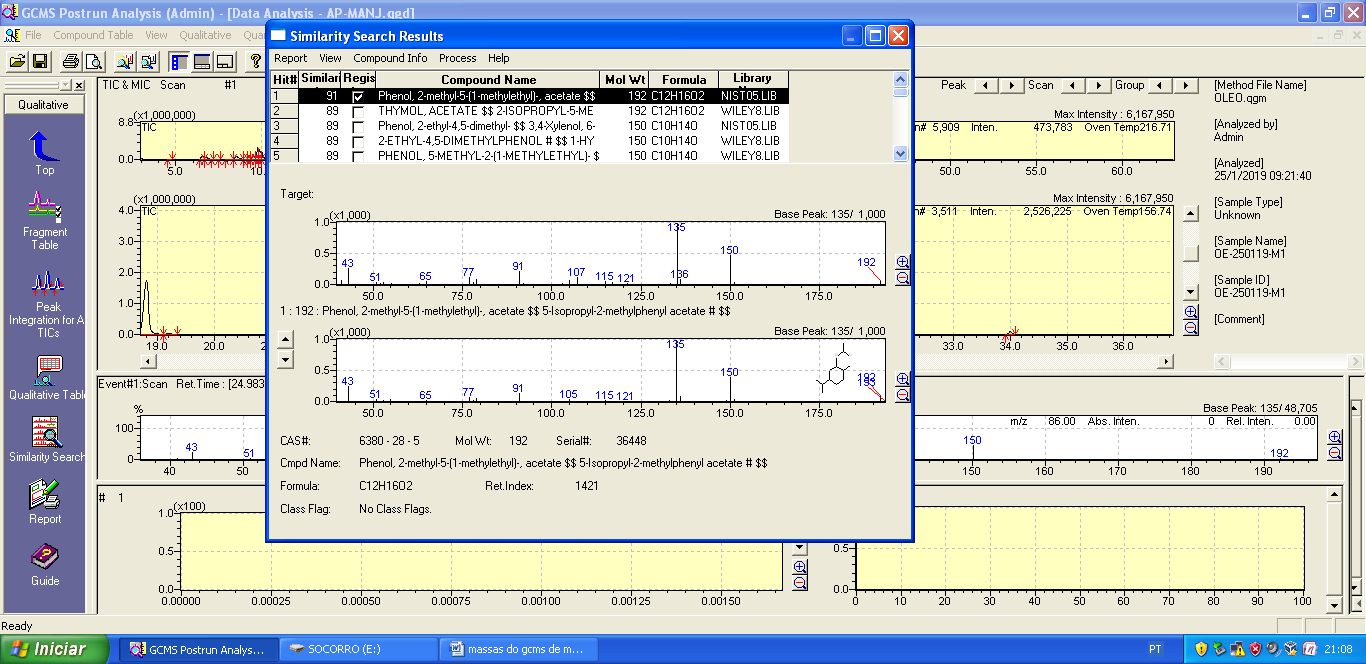


Mass spectrometer for libraries NIST05 e WILEY'S.


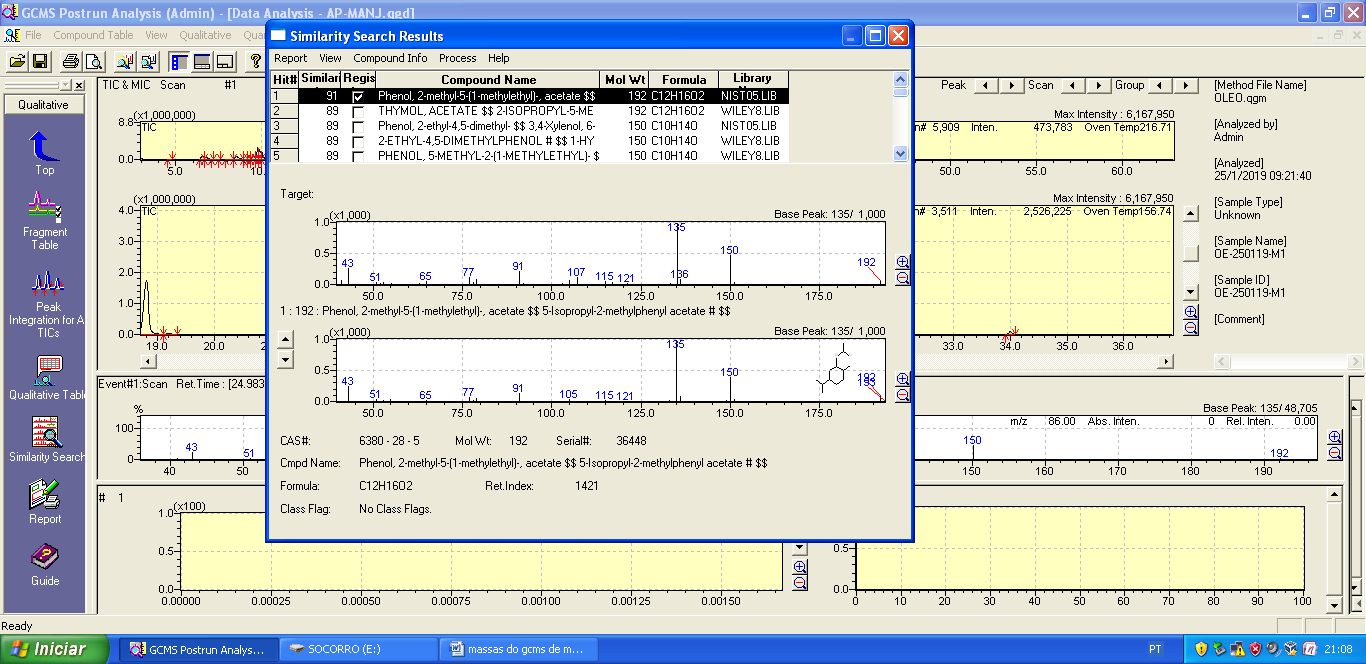


Mass spectrometer of Adams (2017).


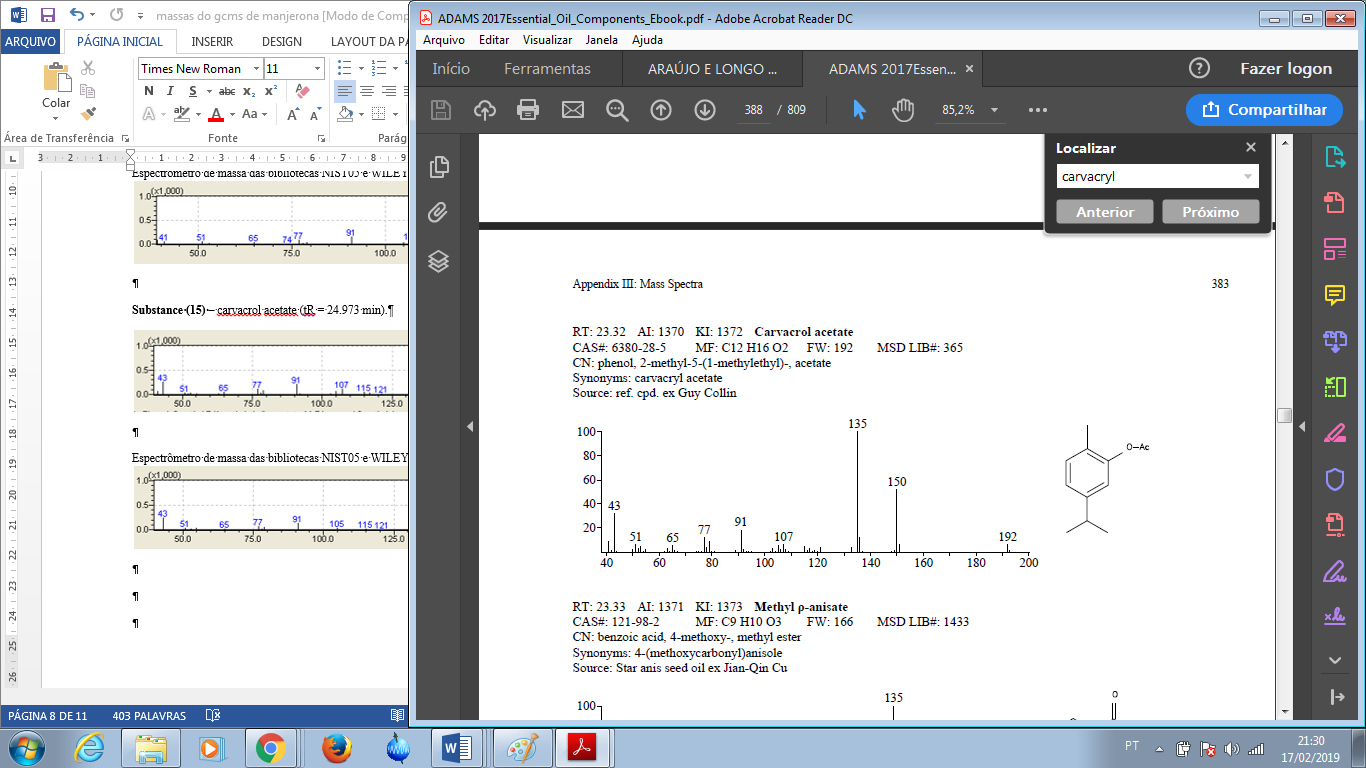


**Substance (16)**- Z-Caryophyllene(tR = 27.187 min).


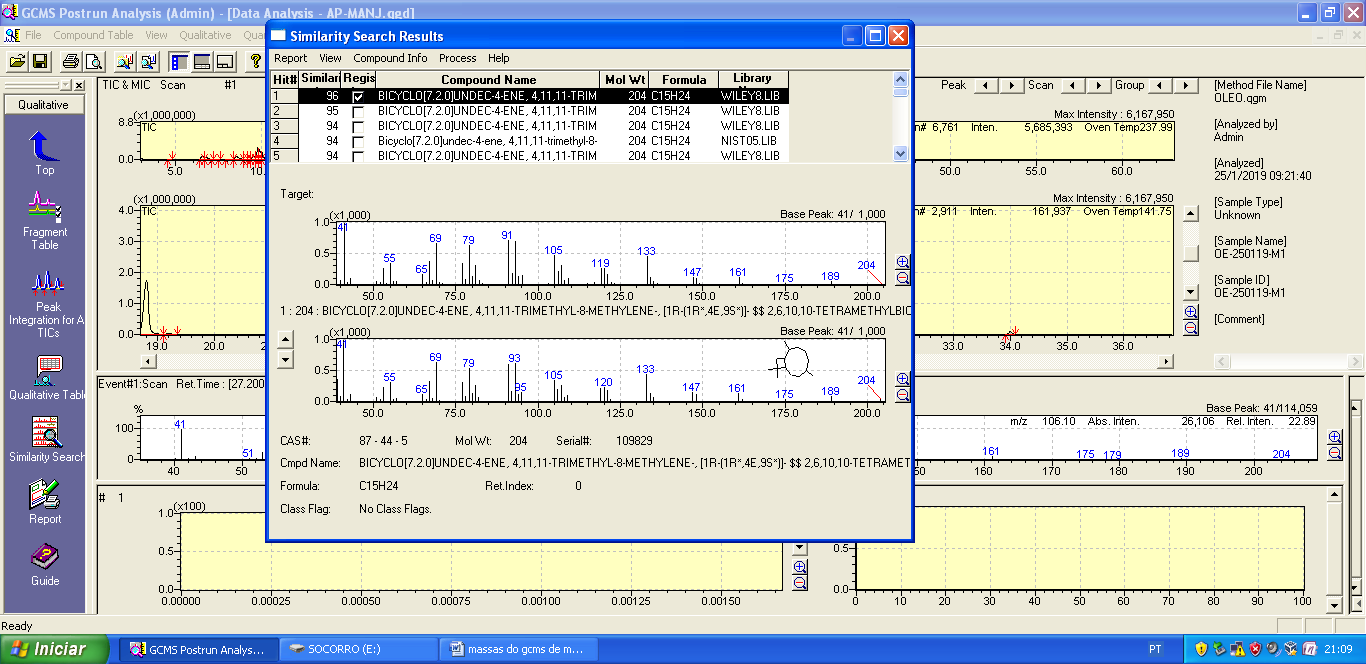


Mass spectrometer for libraries NIST05 e WILEY'S.


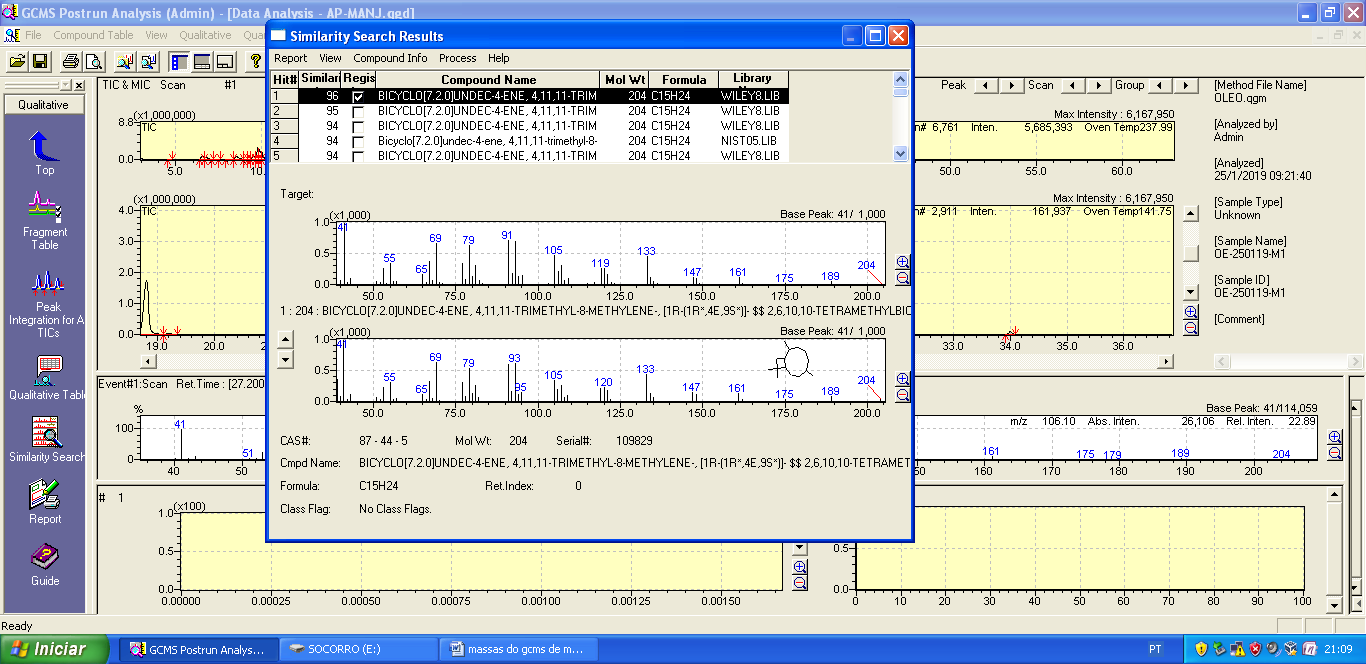


Mass spectrometer of Adams (2017).


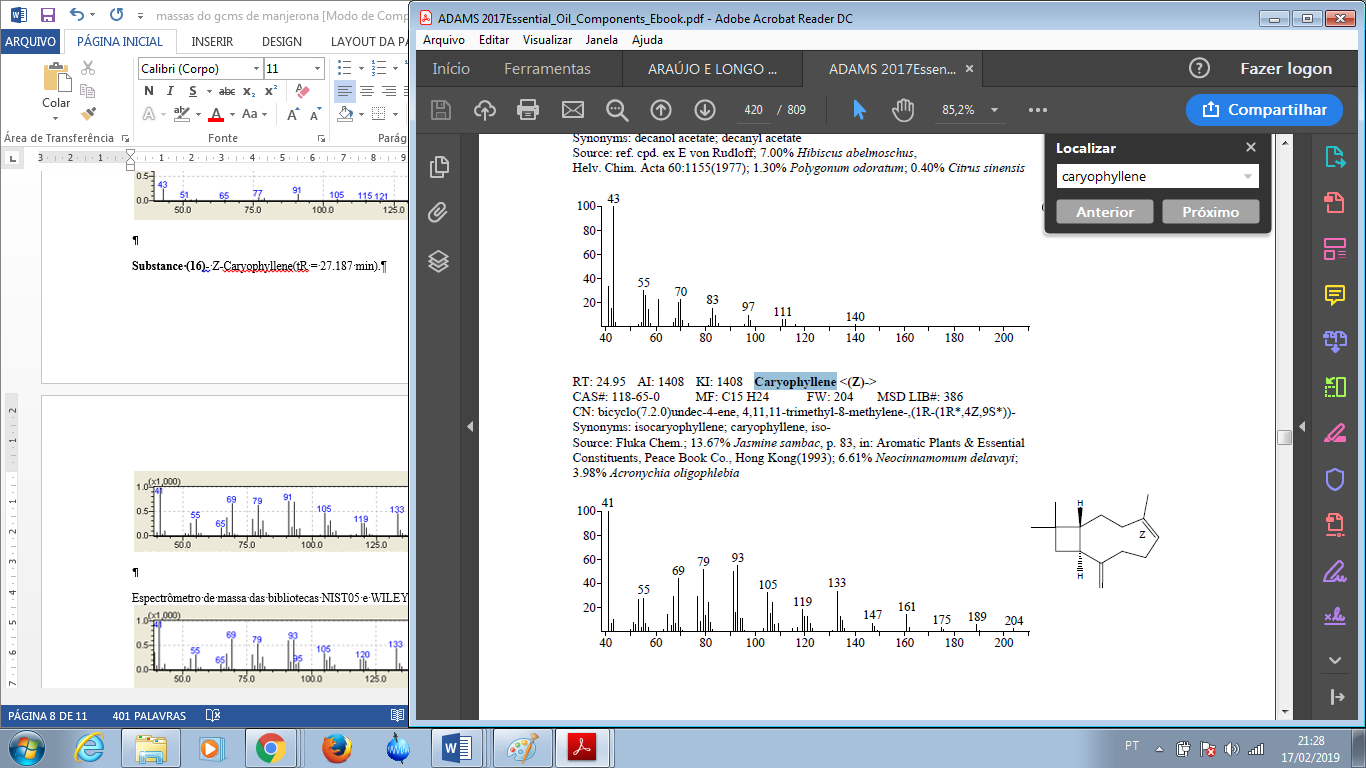


**Substance (17)**- α- Humulene(tR = 28.615 min).


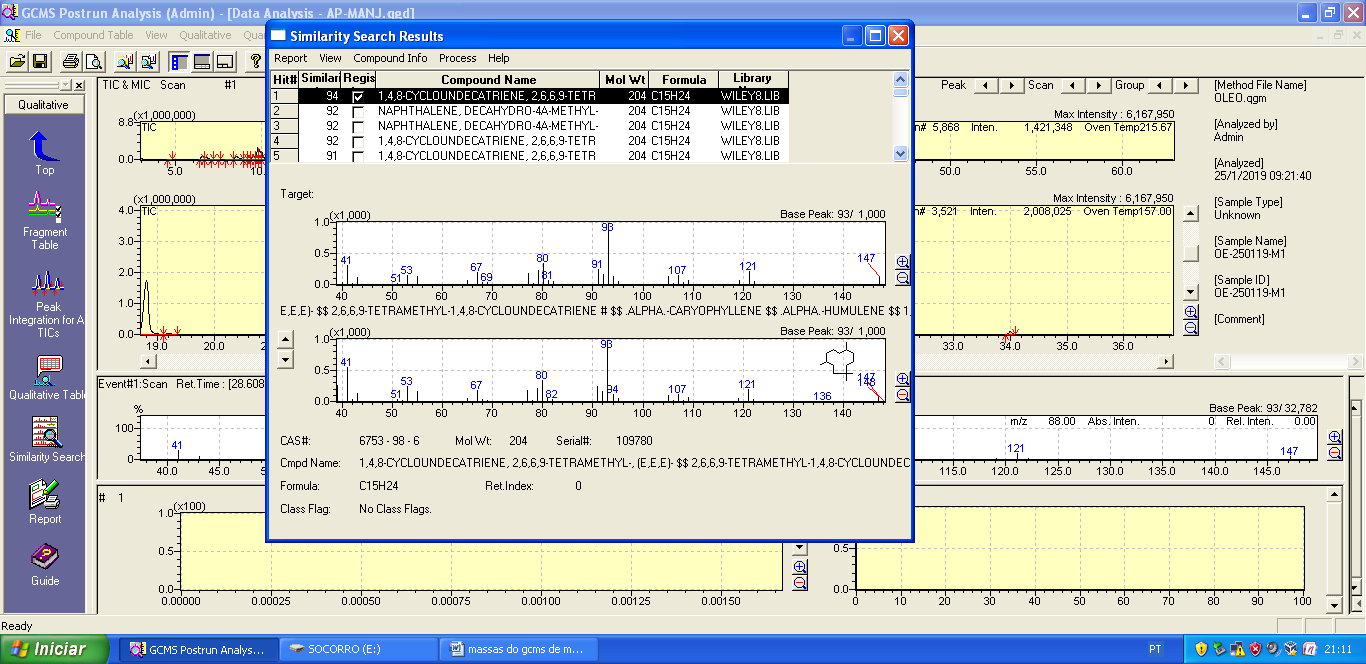


Mass spectrometer for libraries NIST05 e WILEY'S.


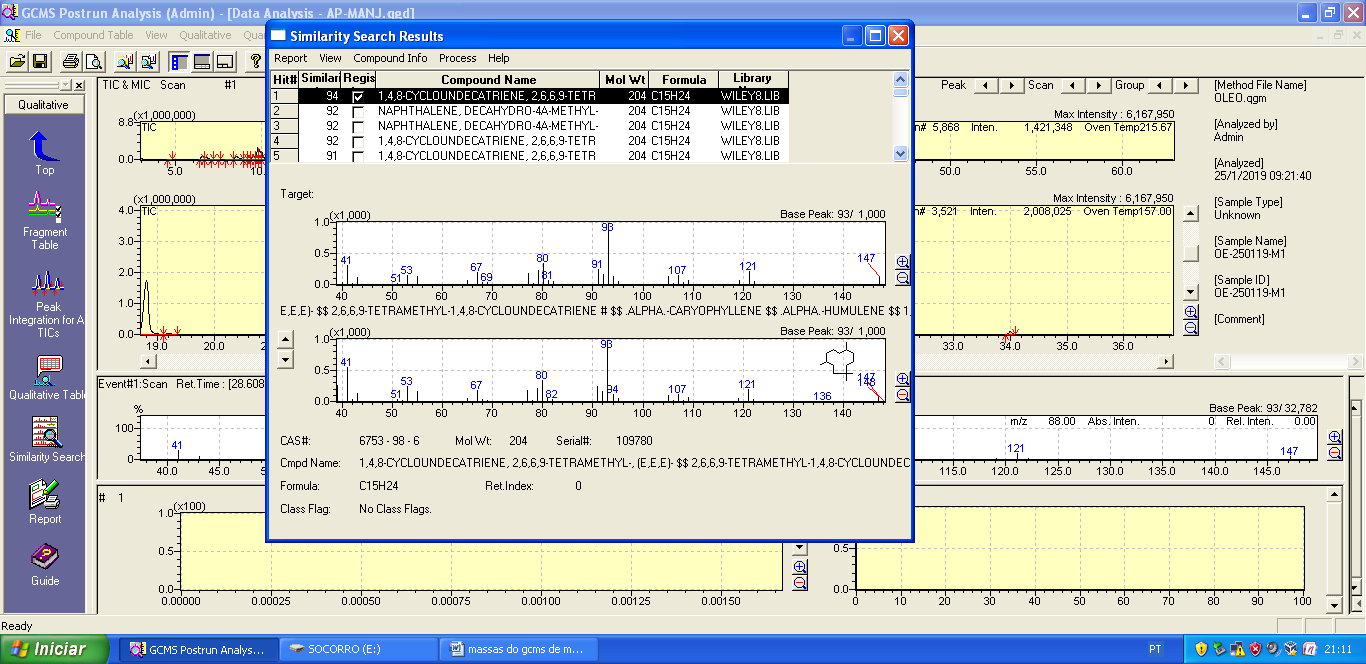


Mass spectrometer of Adams (2017).


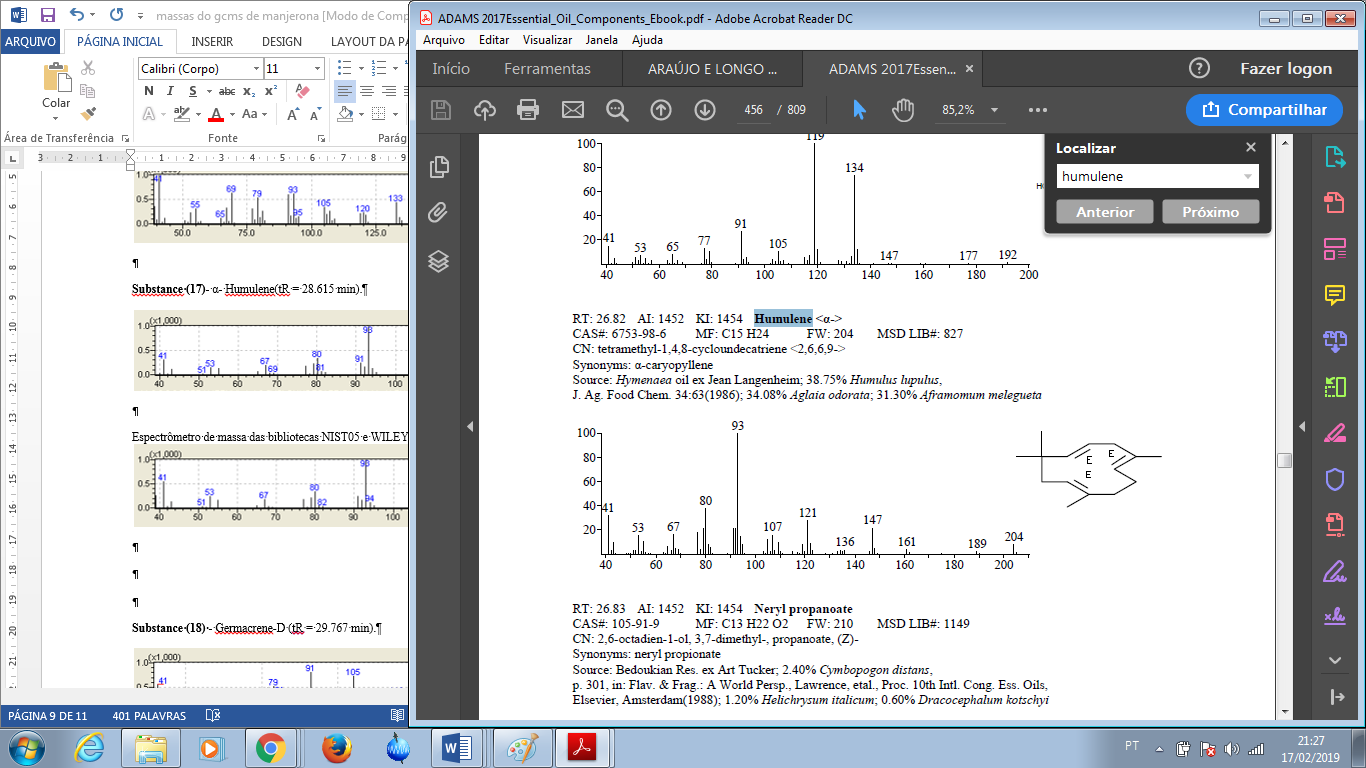


**Substance (18)** - Germacrene-D (tR = 29.767 min).


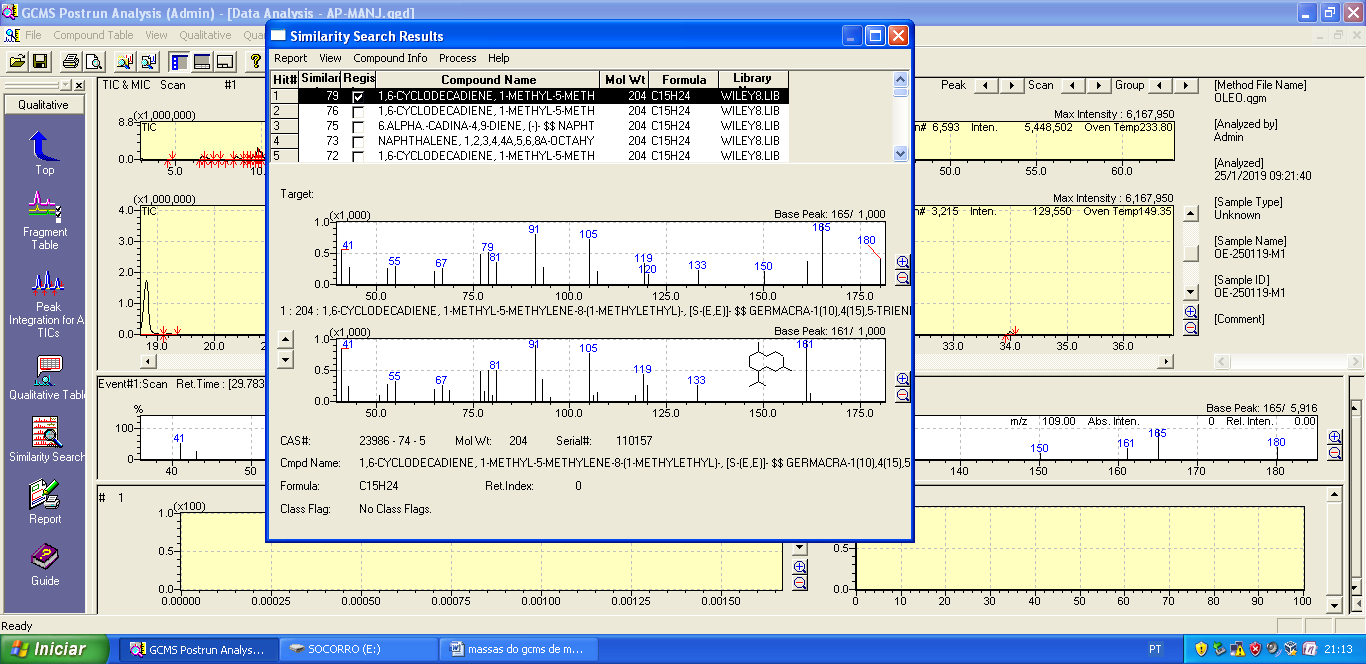


Mass spectrometer for libraries NIST05 e WILEY'S.


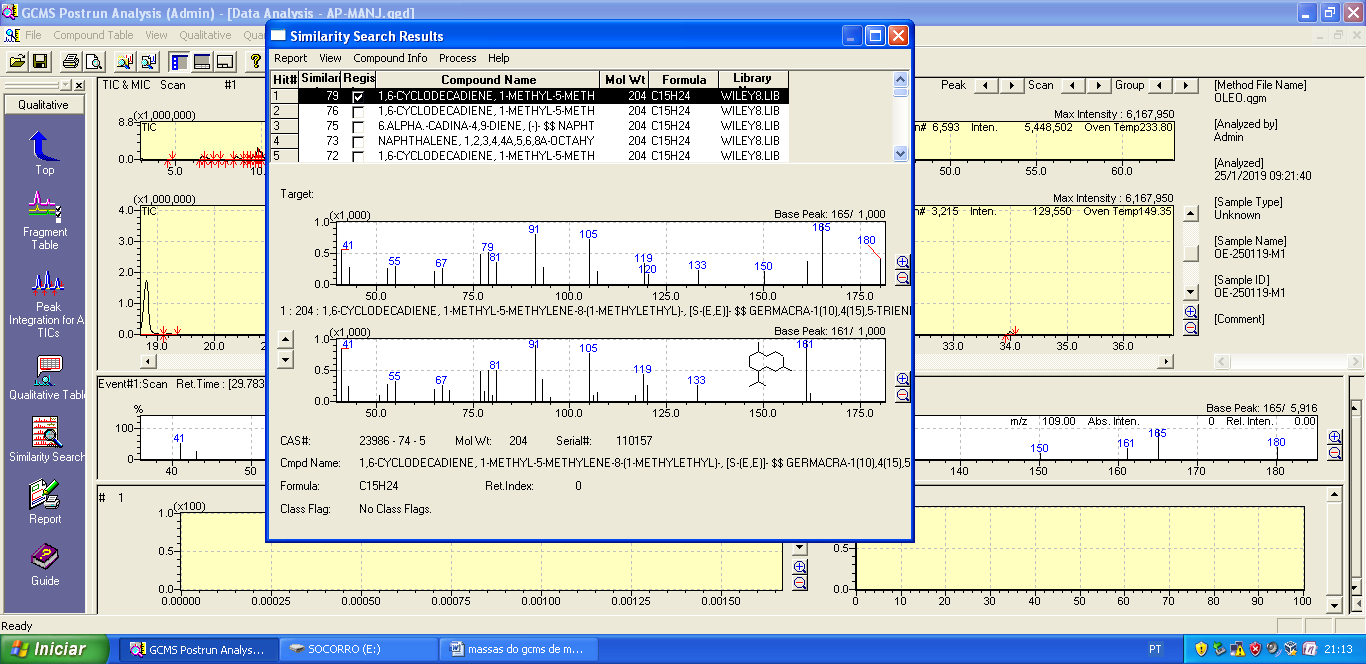


Mass spectrometer of Adams (2017).


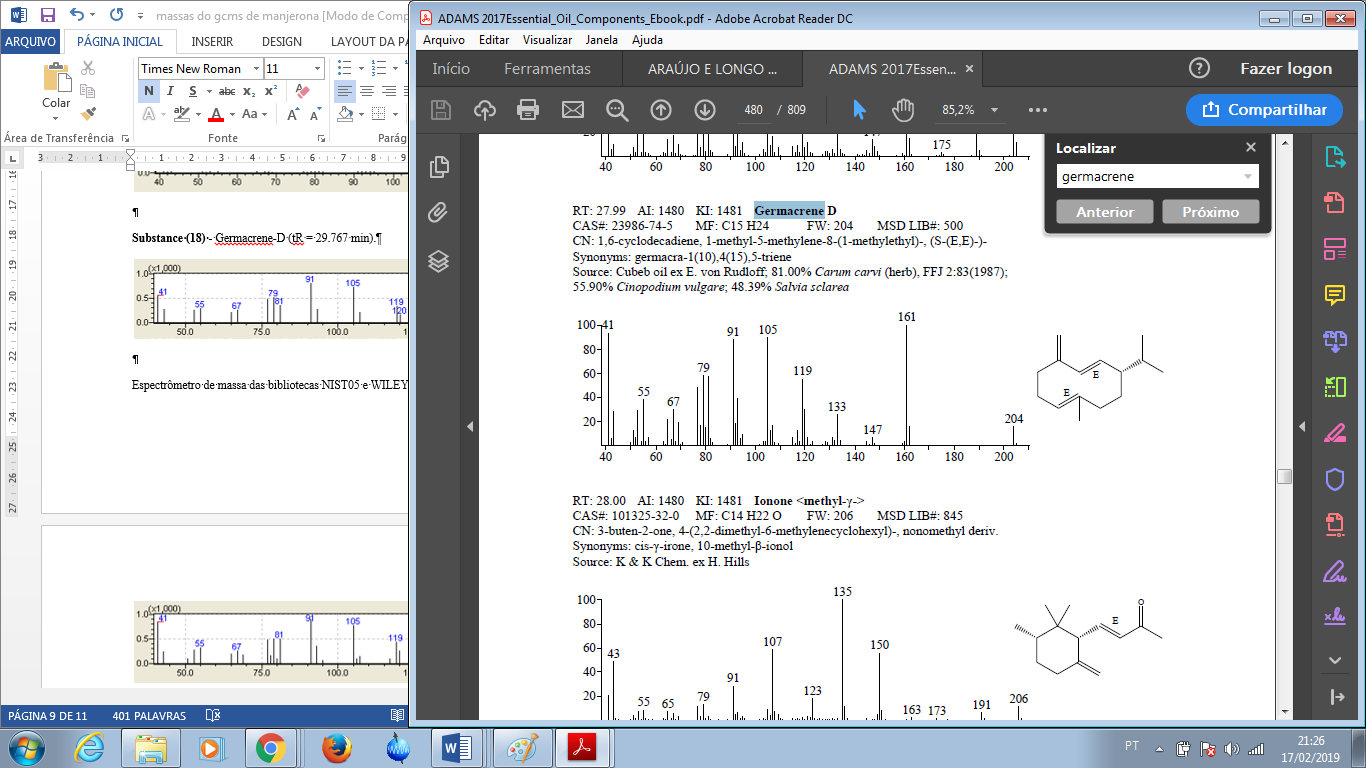


**Substance (19)** – Caryophyllene oxide (tR = 34.001 min).


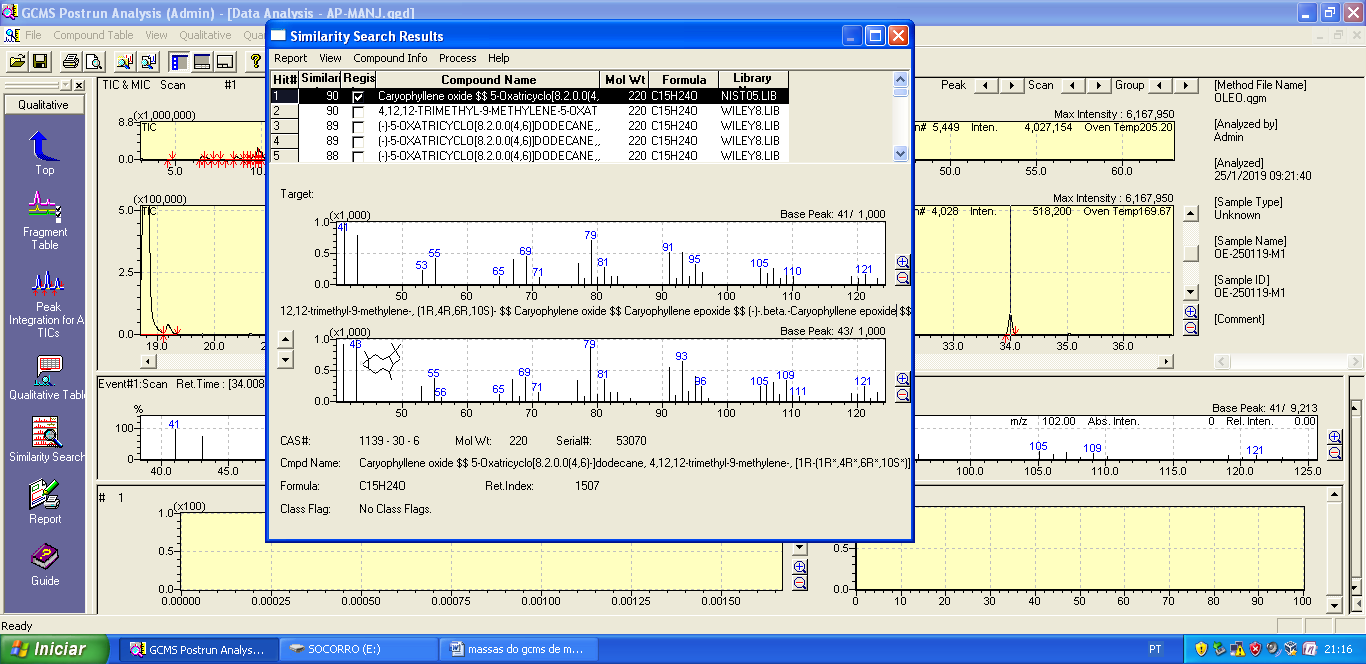


Mass spectrometer for libraries NIST05 e WILEY'S.


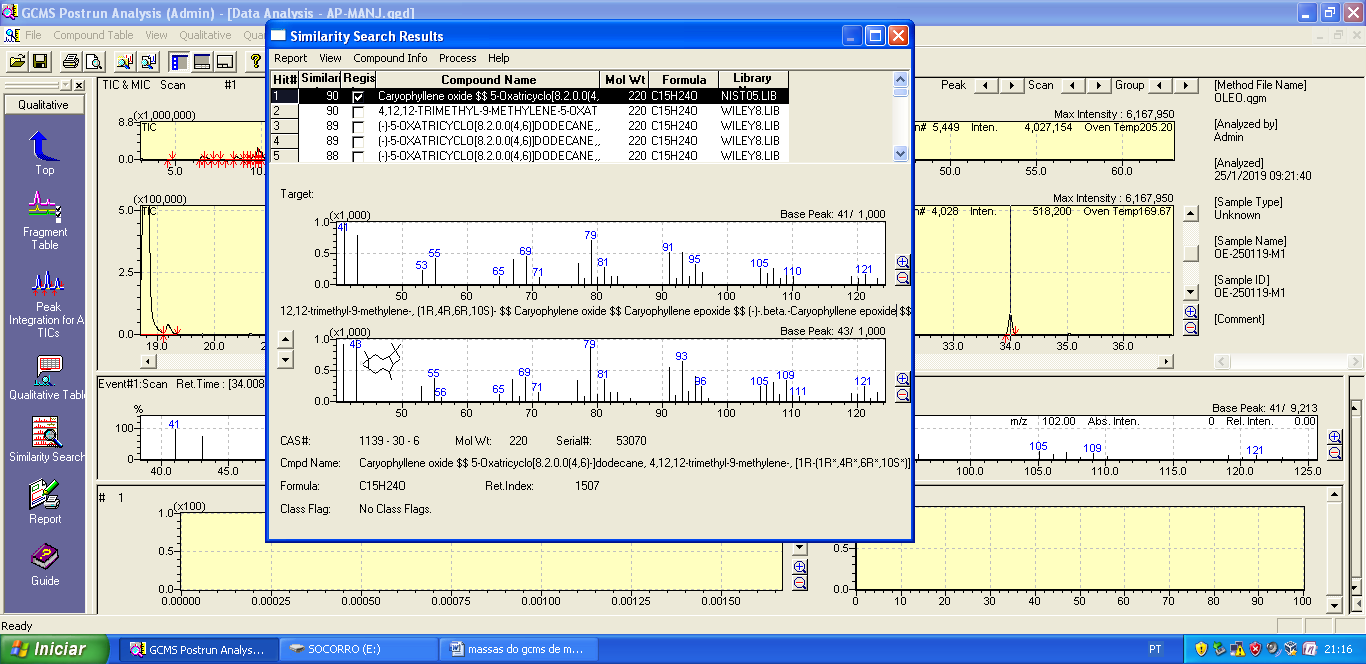


Mass spectrometer of Adams (2017).


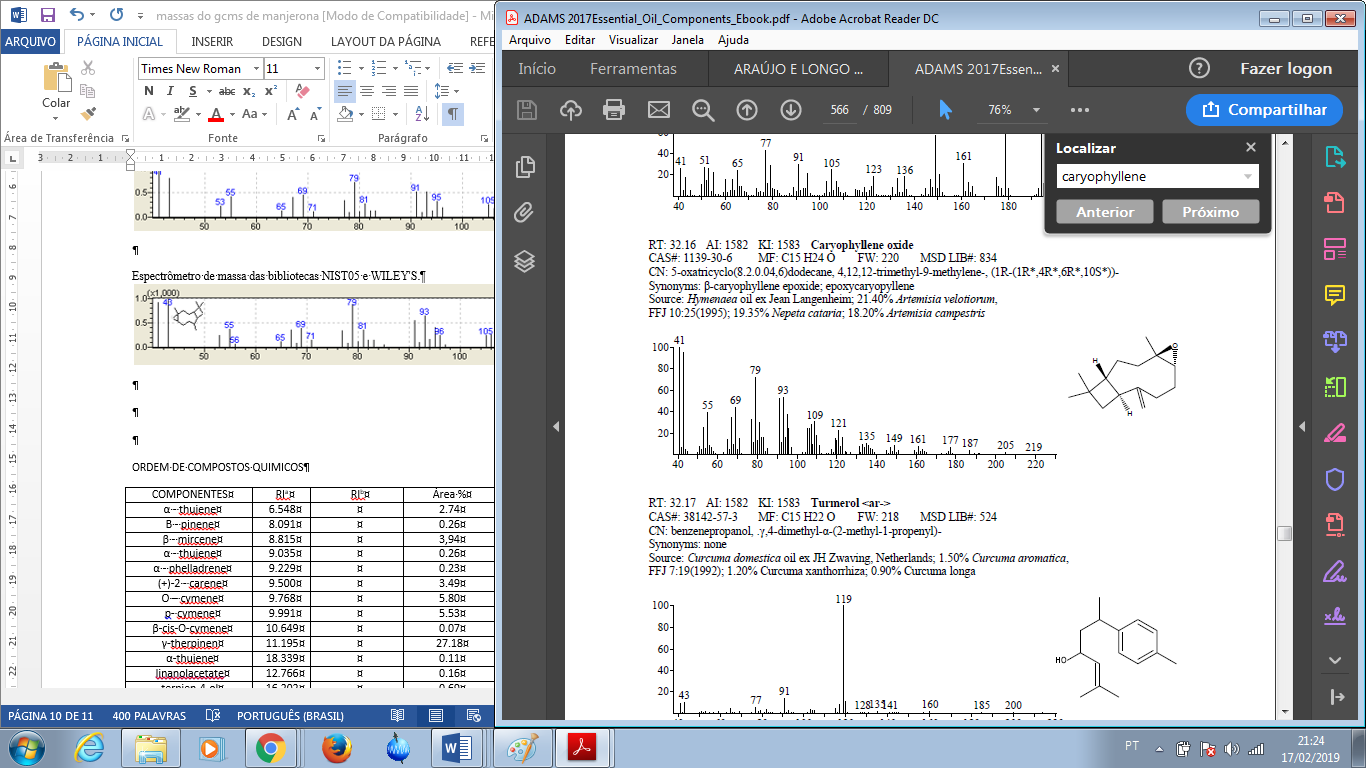

Supplement: S1 File — (DOCX) [file pone.0235740.s001.docx]
