# Supplementary material for: Evaluation of larvicidal potential against larvae of Aedes aegypti (Linnaeus, 1762) and of the antimicrobial activity of essential oil obtained from the leaves of Origanum majorana L
Source: PLoS One. 2020 Jul 17;15(7):e0235740. doi: 10.1371/journal.pone.0235740 (PMC7367459; doi:10.1371/journal.pone.0235740)
Supplement: Attachment — Submitted filename: REBUTTAL LETTER.docx [file pone.0235740.s002.docx]

REBUTTAL LETTER

**Publisher question**

PONE-D-19-08538
Larvicidal evaluation of the Origanum majorana L. Essential Oil against the larvae of the Aedes aegypti mosquito

PLOS ONE

Dear Dr de Almeida,

Thank you for submitting your manuscript to PLOS ONE. After careful consideration, we have decided that your manuscript does not meet our criteria for publication and must therefore be rejected.
After a preliminary assessment of our manuscript it has been found that your figures contain explanation in Portuguese. In addition, the discussion section should be re-written as it is not fluent. The addition of the antimicrobial activities does not fit the title of the manuscript.
You can make these changes and resubmit your manuscript as a new submission to be evaluated for peer-review again.

I am sorry that we cannot be more positive on this occasion, but hope that you appreciate the reasons for this decision.

Yours sincerely,

Horacio Bach

Academic Editor

PLOS ONE

**Authors' response**

The explanations in Portuguese of the firms have already been translated into English, as well as the rewriting of the discussion, by an expert in the field, to make it very fluent.

The title of the manuscript has been changed from: Larvicidal evaluation of the *Origanum majorana* L. Essential oil against the larvae of the *Aedes aegypti* mosquito

For: Evaluation of larvicidal potential against larvae of *Aedes aegypti* (Linnaeus, 1762) and of the antimicrobial activity of essential oil obtained from the leaves of *Origanum majorana* L.

Entered title antimicrobial perform in this search.
