# Supplementary material for: Evaluation of larvicidal potential against larvae of Aedes aegypti (Linnaeus, 1762) and of the antimicrobial activity of essential oil obtained from the leaves of Origanum majorana L
Source: PLoS One. 2020 Jul 17;15(7):e0235740. doi: 10.1371/journal.pone.0235740 (PMC7367459; doi:10.1371/journal.pone.0235740)
Supplement: Attachment — Submitted filename: Response to Revisores.docx [file pone.0235740.s003.docx]

**Response to Revisores**

**Reviewer #1**: Dear authors,

This manuscript deals with the chemical composition of *Origanum majorana* essential oil and its mosquitocidal, antimicrobial, cytotoxic and antioxidant potential. The results of this laboratory study are interesting in that they extend our knowledge about larvicidal potential against *Aedes aegypti* and antimicrobial activity of *Origanum majorana* essential oil. Further down you will find some suggestions of major importance that would probably benefit the manuscript.

Line 126: In the statistical analysis for the larvicidal bioassays it should be noted that the dose response data were analyzed by probit analysis and that LC_50_, LC_90_ values along with the confidential limits and the slope were generated.

**The requirement was met, as can be seen in the lines 158-162 and 163.**

Line 255: In the results concerning the larvicidal effect against mosquitoes, I propose to add a table with the LC_50_ and LC_90_ values with the confidential limits, along with the slope values and x^2^ and R^2^, for the treatment with the essential oil and the positive control at 24 and 48 hours in order for the reader to compare statistically the performance of the treatments.

**The requirement was met, as can be seen in the lines 305-312.**

Line 105: As a major drawback of the study design is that the major compounds, that were identified in the essential oil used, were note tested against *Aedes aegypti* larvae. The performance of the major compounds would help to interpret the toxicity of the essential oil and identify toxic active substances against *Aedes aeypti* larvae. Please comment.

**The requirement was met, as can be seen in the lines 132-134.**

Lines 273-274: The discussion concerning the larvicidal effect of the essential oil should be elaborated more presenting literature data for the larvicidal effect of *O. majorana* essential oil against other mosquito species.

**The requirement was met, as can be seen in the lines 315 – 328.**

Lines 279-282: The larvicidal effect of the major compounds of the essential oil used, namely pulegone, verbenone, trans-menthone, cis-menthone, piperitone, 3-octanol and isopulegol against primarily *Aedes aegypti* and other mosquito species from the literature should be discussed, in order to interpret the action of the current essential oil against *Aedes aegypti*.

**The requirement was met, as can be seen in the lines 293 – 302.**

**Reviewer #2**: The authors studied the larvicidal, antimicrobial, cytotoxic and antioxidant potential of *Origanum majorana* essential oil. Although the topic is relatively interesting and is suitable for publication in PONE, the MS shows some defects that prevent me from recommending the MS for publication. Major comments:

1**. In the introduction**, explain goals of the work better. Why did you choose plant - *Origanum majorana* and why EO and not need extracts - in terms of industrial use of plants. What is the practical impact of work? How does antioxidant capacity and bactericidal activity relate to insecticidal activity? Why other non-target organisms have not been selected.

**The requirement was met, as can be seen in the lines has 53–61 and 86-93.**

**2. Results**: The authors give lethal doses; however, if they want to find correct and relevant values, they should use the probit analysis and compare the CI_95_ intervals. Only in this way can they claim that a significant difference or progression in efficacy in time has been achieved.

**The requirement was met, as can be seen in the lines -310-317.**

The term Positive Control (Table 2) is not correct. This term is used to describe the control variant where a commercial insecticide has been used (to compare biological efficacy of a standard insecticide and EOs).

**The requirement was met, as can be seen in the lines 308 - 309.**

It is not clear if the percentages (eg Table 5) were transformed before statistical analysis.

**No. The percentages were calculated before statistical analysis. Statistical analyzes were performed based on percentages.**

The calculation of LC_50_ (Table 2) for 48 hours is inaccurate because the lowest mortality was 57%, and for 24 hours the mortality for the two different concentrations was the same (40%) - it is not clear which of the values was taken to calculate the lethal concentration.

**The statistical analysis was redone to understand the data more precisely. The requirement has been met, as can be seen on lines 303–305. The two 40% concentrations in Table 2 remain the same in 24 hours, as they are part of the larvae mortality control, but do not compromise the OE larvicidal activity, being more active in 24 h than in 48 h. Mortality decreased dramatically during 48 hours and in the static analysis it presented estimated values with very low and insignificant variation in larval mortality, but this result does not compromise the effective larvicidal activity of the EO.**

3. The discussion should be improved. Chemical composition is very important for the discussion and for understanding the mechanism of action. The authors should give a better comparison of their achieved efficacy to other authors who studied EOs with a majority content of pulegone (see e.g. DOI: 10.17221/48/2013 PPS; <https://doi.org/10.1016/j.indcrop.2015.06.050> ect.) or EOs obtained from *Origanum majorana* against mosquitoes (see e.g. DOI: 10.12980/APJTB.4.2014APJTB-2014-0392; Int J Pharm Pharm Sci, Vol 8, Issue 3, 372-376 ect.)

**The requirement was met, as can be seen in the lines 323-335 e 341–353.**

EO efficacy can be influenced by various external factors including the post-application temperature – this should be discussed (see e.g. DOI: 10.1016/j.indcrop.2018.01.021). The efficacy of sublethal doses or concentrations has been studied – and this should be discussed, as well. The implementation method is yet another aspect to be discussed.

**The requirement was met, as can be seen in the lines -354-377.**

**Tables:**

Line 429: Table 3 changed to **Table 4**

Line 435: Table 4 changed to **Table 5**

Line 4561: Table 4 changed to **Table 6**

**References**

**Line 511 correction**

3. Gonçalves NVS, Rebêlo JMM. [Epidemiological aspects of dengue in the municipality of São Luís, Maranhão, Brazil, 1997-2002]. Public Health Notebooks. 2004; 20: 1424-1431. [Doi](http://dx.doi): 10.1590/S0102-311X2004000500039.

**Line 516 correction**

4. Barbosa IR, Araújo LF, Carlota FC, Araújo RS, Maciel IJ. [Epidemiology of dengue fever in the State of Rio Grande do Norte, Brazil, 2000 to 2009]. Epidemiol Serv Health 2012; 21: 149-57. Doi:V10.5123/S1679-49742012000100015.

**Line 524: correction**

6. Vasconcelos PFC. Zika virus disease: a new emerging problem in the Americas? Rev. PanAmaz. Saúde. 2015; 6: 9-10. Doi: 10.5123/S2176-62232015000200001.

**Line 856: correction**

78. Santos JMP. [Adaptation and Cross-Adaptation of Listeria Spp. Essential Oils from Condensed Plants and Acid Stress]. [dissertation]. Postgraduate Program in Medicinal, Aromatic and Spice Plants: University of Lavras; 2018.

**Line 874: correction**

82. Rodrigues JSQ. [Infusions based on leaves of passifloras of the cerrado: phenolic compounds, antioxidant activity in vitro and sensorial profile]. [dissertation] Universidade de Brasília. 2012.

**Line 629: correction**

31. Anvisa. National Health Surveillance Agency. [Brazilian Pharmacopoeia], 5a ed.; Fiocruz: Brasília, Brasil 2010, pp. 1-545. Português.

**Line 655: correction.**

37. Andrade MA, Cardoso GM, Batista RL, Mallet TCA, Machado FMS. [Essential Oils of *Cymbopogon Nardus*, *Cinnamomum Zeylanicum* and *Zingiber Officinale*: composition, antioxidant and antibacterial activities]. J Agron Sci. 2012; 43 (2): 399-408. Doi: 10.1590/S1806-66902012000200025. Português.

**Line 532-567 Added in References.**

8. Zara, AL.; Santos, SM. dos; Oliveira-Fernandes, ES.; Carvalho, RG.; Coelho, GE. [Strategies for controlling *Aedes aegypti*: a review]. Epidemiol Serv Health. 2016; 25 (2): 391-404. Doi: 10.5123/s1679-49742016000200017.

9. Araújo VEM, Bezerra JMT, Frederico FA, Passos VMA, Carneiro M. [Increased burden of dengue in Brazil and federated units, 2000 and 2015: analysis of the *Global Burden of Disease Study* 2015]. Brazilian Journal of Epidemiology. 2017; 20: 205-216. Doi: 10.1590/1980-5497201700050017.

10. Wang X, Li JL ,Xing HJ, Xu SW. Review of the toxicology of atrazine and chlorpyrifos onfish. Journal Northeast Agric Univ, 2011, (18): 88-92.

11. Mohammed MP, Penmethsa KK. Assessment of pesticide residues in surface waters of Godavari delta, India. Journal Mater Environ Sci, 2014, (5): 33-6.

12. Djogbénou L. Vector controls methods against malaria and vector resistance to insecticides in Africa. Med Trop, 2009, (69): 160-4.

13. El Ouali Lalami A, El-Akhal F, El Amri N, Maniar S, Faraj C. State resistance of the mosquito *Culex pipiens* towards temephos central Morocco. Bull Soc Pathol Exot Ses Fil, 2014, (107): 194-8.

14. Azizi A, Yan F, Honermeier B. Herbage yield, essential oil content and composition of three oregano (*Origanum vulgare* L.) populations as affected by soil moisture regimes and nitrogen supply. Ind Crops Prod 2009, (29): 554-61.

15. Abdel-Massih RM, Abraham A. Extracts of *Rosmarinus officinalis, Rheum rhaponticum*, and *Origanum majorana* exhibit significant anti-staphylococcal activity*, International Journal of Pharmaceutical Sciences and Research*, 2014, (5): 819– 828.
16. Busatta C, Vidal RS, Popiolski AS. Application of *Origanum majorana* L. essential oil as an antimicrobial agent in sausage. Food Microbiology. 2008, 25 (1): 207–211.
17. Prakash B, Singh P, Kedia A, Dubey NK. Assessment of some essential oils as food preservatives based on antifungal, antiaflatoxin, antioxidant activities and *in vivo* efficacy in food system. Food Research International, 2012, 49.(1): 201–208.

**Line 588-616 Added in References.**

22. Dhaheri YAl, Attoub S, Arafat K. Anti-metastatic and anti-tumor growth effects of *Origanum majorana* on highly metastatic human breast cancer cells: inhibition of NF𝜅B signaling and reduction of nitric oxide production. PLoS ONE, 2013, 8, (7) Article IDe68808.

23. Rao S, Timsina B, Nadumane VK. Evaluation of the anticancer potentials of *Origanum marjorana* on fibrosarcoma (HT-1080) cell line. Asian Pacific Journal of Tropical Disease, 2014, 4 (1): S389–S394.

24. Ramadan G, El-Beih NM, Zahra MM. Egyptian sweet marjoram leaves protect against genotoxicity, immunosuppression and other complications induced by cyclophosphamide in albino rats. British Journal of Nutrition, 2012, 108,.(6): 1059– 1068.

25..Khan JA, Jalal J.A, Ioanndes C,. Moselhy SS. Impact of aqueous doash extract on urinary mutagenicity in rats exposed to heterocyclic amines. Toxicology and Industrial Health, 2013, 29, (2): 142–148.

26. V´agi E, Sim´andi B, Suhajda´ Aethelyi´ EH. Essential oil composition and antimicrobial activity of *Origanum majorana* L. extracts obtained with ethyl alcohol and supercritical carbon dioxide, Food Research International, 2005, 38 (1): 51–57.

27. Ebrahimi, M, Khosravi-Darani, K, Essential oils as natural food preservatives: antimicrobial and antioxidant applications. In: Doughari, James (Ed.), Antimicrobials from Nature: Effective Control Agents for Drug Resistant Pathogens. 2013, Transworld Research Network, Kerala, (2): 37-661, ISBN: 978-81-7895-603-9

28. Bassalé, JRN., Juliani, H.R., (2012). Essencial oils in combination and their antimicrobial properties. Molecules, 3989-4006.

30. Pereira ÁIS, Pereira AGS, Sobrinho OPL, Cantanhede EKP, Siqueira LFS. [Antimicrobial activity in the control of larvae of the mosquito *Aedes aegypti*: Homogenization of the essential oils of linalool and eugenol]. Chemical education 2014; 25 (4): 446-449. [Doi: 10.1016/S0187-893X(14)70065-5](https://doi.org/10.1016/S0187-893X(14)70065-5).

**Line 754-805 Added in References.**

56. El-Akhal F, Greche H, Ouazzani CF, Guemmouh R, El Ouali Lalami A. Chemical composition and larvicidal activity of Culex pipiens essential oil of Thymus vulgaris grown in Morocco. JMater Environ Sci 2015;1:214-9.

57. El-Akhal F, El Ouali Lalami A, Ez Zoubi Y, Greche H, Guemmouh R. Chemical composition and larvicidal activity of essential oil of Origanum majorana (Lamiaceae) cultivated in Morocco against Culex pipiens (Diptera: Culicidae). Asian Pac J Trop Biomed2014;4:746-50.

58. Szczepanik M, Zawitowska B, Szumny A. Insecticidal activities of Thymus vulgaris essential oil and its components (thymol and carvacrol) against larvae of lesser mealworm, Alphitobius diaperinus Panzer (Coleoptera Tenebrionidae). Allelopathy J2012;30:129-42.

59. Cavalcanti ESB, de Morais SM, Lima MAA, Santana EWP. Larvicidal activity of essential oils from Brazilian plants against Aedes aegypti L. Mem Inst Oswaldo Cruz 2004;99:54 1-4.

60. Pavela R, Kaffková K, Kumsta M. Chemical composition and larvicidal activity of essential oils from different *Mentha* L. and *Pulegium* species against *Culex quinquefasciatus* Say (Diptera: Culicidae). Plant Protect. Sci. 2014; (50): 36–42. [DOI: 10.17221/48/2013-PPS](https://doi.org/10.17221/48/2013-PPS)

61. Koliopoulos G., Pitarokili D., Kioulos E., Michaelakis A., Tzakou O. (2010): Chemical composition and larvicidal evaluation of Mentha, Salvia and Melissa essential oils against the West Nile virus mosquito Culex pipiens. Parasitology Research, 107: 327-375.

62. Lucia A, Zerba E, Masuh H. Knockdown and larvicidal activity of six monoterpenes against Aedes aegypti (Diptera: Culicidae) and their structure-activity relationships. Parasitol Research. 2013, (112): 4267-4272. DOI: 10.1007/s00436-013-3618-6.

63. El-Akhal F, Guemmouh R, Maniar S, Taghzouti K, Lalami A E O, Larvicidal Activity of Essential Oils of Thymus Vulgaris and Origanum Majorana (Lamiaceae) Against of the Malaria Vector Anopheles Labranchiae (Diptera: Culicidae). 2016, International Journal of Pharmacy and Pharmaceutical Sciences. (8); 372-376, issue 3, DOI: 10,12980 / APJTB.4.2014APJTB-2014-0392

64. Cavalcanti ESB, de Morais SM, Lima MAA, Santana EWP. Larvicidal activity of essential oils from Brazilian plantsagainst *Aedes aegypti* L. Mem Inst Oswaldo Cruz2004;99:541-4.

65. Pavela R, Sedlák P. Post-application temperature as a factor influencing the insecticidal activity of essential oil from Thymus vulgaris. Industrial Crops e Products. 2018, (113); 46-49. DOI: 10.1016 / j.indcrop.2018.01.021

66. Pavela R. History, presence and perspective of using plant extracts as commercial botanical insecticides and farm products for protection against insects –a review. Plant Prot. Sci. 2016, (52): 229–241.

67. Dias CN, Moraes DFC. Essential oils and their compounds as *Aedes aegypti* L. (Diptera: Culicidae) larvicides: review. Parasitology Research. 2014, 113 (1): 565- 592.
