# Supplementary material for: Evaluation of larvicidal potential against larvae of Aedes aegypti (Linnaeus, 1762) and of the antimicrobial activity of essential oil obtained from the leaves of Origanum majorana L
Source: PLoS One. 2020 Jul 17;15(7):e0235740. doi: 10.1371/journal.pone.0235740 (PMC7367459; doi:10.1371/journal.pone.0235740)
Supplement: Attachment — Submitted filename: Response to Reviewers-1.docx [file pone.0235740.s004.docx]

**Response to Reviewers**

**1. Please amend your list of authors on the manuscript to ensure that each author is linked to an affiliation.**

We note that you have included affiliation symbols '&' and '¶' beside author names, however there are no affiliations linked to these symbols.

Please amend to link an affiliation 4 to each or remove if added in error.

**The requirement was met, as can be seen in the lines 5-8.**

**Renata do Socorro Barbosa Chaves^1^, Rosany Lopes Martins^1^, Alex Bruno Lobato Rodrigues^1^, Érica de Menezes Rabelo^1^, Ana Luzia Ferreira Farias^1^, Lethicia Barreto Brandão^1^, Lizandra Lima Santos^1^, Camila Mendes da Conceição Vieira Araújo^2^, Talita Fernandes Sobral^2^, Allan Kardec Ribeiro Galardo^2^, Sheylla Susan Moreira da Silva de Almeida^1*^**

**2. Thank you for including your ethics statement: 'N/A' and thank you for stating in your methods section that:**

'The leaves of *O. majorana* L. were collected in the district of Fazendinha (00 "36'955" S and 51 "11'03'77" W) in the Municipality of Macapá, Amapá. Five samples of the plant species were deposited at the Amapaense Herbarium (HAMAB) of the Institute of Scientific Research and Technology of Amapá (IEPA).'

To comply with PLOS ONE submissions requirements for field studies, please provide the following information in the Methods section of the manuscript and in the “Ethics Statement” field of the submission form (via “Edit Submission”):

a) Provide the name of the authority who issued the permission for each location (for example, the authority responsible for a national park or other protected area of land or sea, the relevant regulatory body concerned with protection of wildlife, etc.). If the study was carried out on private land, please confirm that the owner of the land gave permission to conduct the study on this site.

**The requirement was met, as can be seen in the lines 103.**

**a) The study was carried out on private land with permission from the owner.**

b) For any locations/activities for which specific permission was not required, please

- i. state clearly that no specific permissions were required for these locations/activities, and provide details on why this is the case

- ii. confirm that the field studies did not involve endangered or protected species

**The requirement was met, as can be seen in the lines 104-106.**

**i. There was no need for specific permissions for the study site/activity, as it is a private property where the owner has given authorization to collect the plant material.**

**ii. The study did not involve threatened or endangered species.**
