# Supplementary material for: Evaluation of larvicidal potential against larvae of Aedes aegypti (Linnaeus, 1762) and of the antimicrobial activity of essential oil obtained from the leaves of Origanum majorana L
Source: PLoS One. 2020 Jul 17;15(7):e0235740. doi: 10.1371/journal.pone.0235740 (PMC7367459; doi:10.1371/journal.pone.0235740)
Supplement: Attachment — Submitted filename: Response to reviewers (1).docx [file pone.0235740.s005.docx]

**Response to reviewers**

**Comments to the Author**

6. Review Comments to the Author

Please use the space provided to explain your answers to the questions above. You may also include additional comments for the author, including concerns about dual publication, research ethics, or publication ethics. (Please upload your review as an attachment if it exceeds 20,000 characters)

Reviewer #1: Dear authors,

My comments were partially addressed in the manuscript not affecting, however, the robustness of the study. As a final comment, please check if the LC_50_ values for the positive control (esbiothrin) against mosquito larvae were indeed the same for 24 and 48 records of mortality.

**The requirement was met, as can be seen in the lines 298-300 and in the table 305-306.**

Dear reviewer, analyzing **Table (in the lines 312-313)**, it was necessary to **use bold** in the title of table 3; **remove the spacing** that existed between the title and table 3; **add the parenthesis** after the LC_50_ value 124.17 (103.23; 171.63**)** in 24 h, **apply the superscript and subscript** of the LC**_50_** values (IC**_95_**) µg.mL**^-1^**, CL**_90_** (IC**_95_**) µg.mL**^-1^**, R**^2^**, X**^2^** and apply italic in ***X^2^***. In addition, it was necessary **to indent the paragraph** after table 3 **(in the lines 314-315)**.

**Regarding the corrections in the references, the word "Portuguese" was removed on lines 357, 625, 656, 857, 874, 892 and 905.**
